# Transcription factor-based transdifferentiation of human embryonic to trophoblast stem cells

**DOI:** 10.1242/dev.202778

**Published:** 2024-09-10

**Authors:** Paula A. Balestrini, Ahmed Abdelbaki, Afshan McCarthy, Liani Devito, Claire E. Senner, Alice E. Chen, Prabhakaran Munusamy, Paul Blakeley, Kay Elder, Phil Snell, Leila Christie, Paul Serhal, Rabi A. Odia, Mahesh Sangrithi, Kathy K. Niakan, Norah M. E. Fogarty

**Affiliations:** ^1^ Centre for Gene Therapy and Regenerative Medicine, King's College London, London SE1 9RT, UK; ^2^ Human Embryo and Stem Cell Laboratory, The Francis Crick Institute, 1 Midland Road, London NW1 1AT, UK; ^3^ The Centre for Trophoblast Research, Department of Physiology, Development and Neuroscience, University of Cambridge, Cambridge CB2 3EG, UK; ^4^ Department of Zoology, Faculty of Science, Zagazig University, Zagazig 44519, Egypt; ^5^ Human Embryo and Stem Cell Unit, The Francis Crick Institute, 1 Midland Road, London NW1 1AT, UK; ^6^ Trestle Biotherapeutics, Centre for Novel Therapeutics, 9310 Athena Circle, La Jolla, CA 92037, USA; ^7^ KK Women's and Children's Hospital, Division of Obstetrics and Gynecology, 100 Bukit Timah Road, Singapore 229899, Singapore; ^8^ Department of Surgery, School of Clinical Medicine, The University of Hong Kong, Hong Kong SAR, China; ^9^ Bourn Hall Clinic, Bourn, Cambridge CB23 2TN, UK; ^10^ The Centre for Reproductive & Genetic Health, 230–232 Great Portland Street, London W1W 5QS, UK; ^11^ Duke-NUS Graduate Medical School, Cancer Stem Cell Biology/OBGYN ACP, 8 College Road, Singapore 169857, Singapore; ^12^ Wellcome Trust – Medical Research Council Stem Cell Institute, University of Cambridge, Jeffrey Cheah Biomedical Centre, Puddicombe Way, Cambridge CB2 0AW, UK; ^13^ Epigenetics Programme, Babraham Institute, Cambridge CB22 3AT, UK

**Keywords:** Trophectoderm, Embryonic stem cells, Trophoblast stem cells, Human embryo, Transdifferentiation

## Abstract

During the first week of development, human embryos form a blastocyst composed of an inner cell mass and trophectoderm (TE) cells, the latter of which are progenitors of placental trophoblast. Here, we investigated the expression of transcripts in the human TE from early to late blastocyst stages. We identified enrichment of the transcription factors *GATA2*, *GATA3*, *TFAP2C* and *KLF5* and characterised their protein expression dynamics across TE development. By inducible overexpression and mRNA transfection, we determined that these factors, together with MYC, are sufficient to establish induced trophoblast stem cells (iTSCs) from primed human embryonic stem cells. These iTSCs self-renew and recapitulate morphological characteristics, gene expression profiles, and directed differentiation potential, similar to existing human TSCs. Systematic omission of each, or combinations of factors, revealed the crucial importance of GATA2 and GATA3 for iTSC transdifferentiation. Altogether, these findings provide insights into the transcription factor network that may be operational in the human TE and broaden the methods for establishing cellular models of early human placental progenitor cells, which may be useful in the future to model placental-associated diseases.

## Teaser

**Summary:** Transcriptional analysis of human blastocysts reveals transcription factors sufficient to derive induced trophoblast stem cells from primed human embryonic stem cells.

## INTRODUCTION

The correct functioning of the human placenta is crucial to ensure a healthy pregnancy outcome. However, despite this essential role, it is one of the least understood organs. After fertilisation, the zygote undergoes a series of cleavage cell divisions to form a tight ball of cells called a morula. The outer cells of the morula become polarised, which leads to inactivation of the Hippo signalling pathway and allows the translocation of YAP1 into the nucleus where, together with TEAD4, it drives transcriptional activation of the trophectoderm (TE) programme (Gerri et al., 2020). The human blastocyst implants into the uterine lining approximately 7-9 days after fertilisation. The TE generates the first trophoblast lineages: the mononuclear cytotrophoblast (CTB) cells and the multinucleated primitive syncytiotrophoblast (STB) that together contribute to the fetal portion of the placenta. The primitive STB is the initial invading interface and enzymatically degrades the uterine lining allowing for implantation of the conceptus (Hertig et al., 1956). CTB cells are the precursor for trophoblast cell types in the placenta. CTB cells sustain the STB across gestation by dividing asymmetrically, with one daughter cell fusing with the overlying STB and the other daughter remaining in the proliferative pool (Baczyk et al., 2006). The STB performs key functions, including providing a physical and immunological barrier to pathogens, transporting nutrients and gaseous exchange, as well as producing and secreting hormones required to adapt the maternal physiology to the pregnancy (Bansal et al., 2012; Costa, 2016). CTB cells are also the precursor of extravillous trophoblast cells (EVTs), which invade the decidua to interact with maternal immune cells and remodel spiral arteries to establish blood supply to the placenta (Pijnenborg et al., 1980).

Human trophoblast stem cells (hTSCs) derived from blastocysts and first trimester placental villous tissue provide a paradigm for elucidating molecular mechanisms regulating trophoblast development (Okae et al., 2018). hTSCs show expression of well-established markers of trophoblast, including TP63 (Okae et al., 2018), GATA3 (Chiu and Chen, 2016) and TEAD4 (Soncin et al., 2018), and can be directed to differentiate into both EVT and STB, as identified by the expression of the markers HLA-G (Apps et al., 2008) and SDC1 (Jokimaa et al., 1998), respectively. In addition, the establishment of a trophoblast organoid culture system has allowed hTSCs to be cultured in 3D, somewhat resembling the architecture of villous CTB cells and STB of the placenta (Haider et al., 2018; Turco et al., 2018). Most recently, induced trophoblast stem cells (iTSCs), which resemble primary tissue-derived hTSCs, have been captured during the process of reprogramming fibroblasts to human embryonic stem cells (hESCs) in naïve cell culture conditions (Castel et al., 2020; Naama et al., 2023) as well as by culturing naïve hESCs directly in hTSC conditions (Cinkornpumin et al., 2020; Dong et al., 2020). In addition, established naïve ESCs cultured in conditions that inhibit ERK and Nodal signalling also yield iTSCs (Guo et al., 2021). These *in vitro* models can be utilised in studies of trophoblast biology and have the potential to further inform our understanding of molecular mechanisms, transcription factors and signalling pathways regulating human trophoblast biology.

Studies of mouse pre-implantation embryogenesis have elucidated mechanisms regulating lineage specification and maintenance of the TE in this species (Hemberger et al., 2020). In turn, this knowledge has led to the development of strategies to capture their *in vitro* counterparts. Blastocyst-derived TSCs can be isolated from the polar TE of blastocyst outgrowths or post-implantation extra-embryonic ectoderm cultured in TSC media supplemented with FGF4, heparin, TGFβ and activin, which recapitulates the signalling environment of the *in vivo* TSC compartment at the post-implantation stage (Natale et al., 2009; Tanaka et al., 1998). Identification of key transcription factors regulating mouse TE lineage specification and maintenance has also informed transdifferentiation strategies. Single-factor overexpression of *Cdx2*, *Eomes*, *Tcfap2c*, *Tead4* or *Gata3* in mouse ESCs in TSC media is sufficient to induce transdifferentiation to trophoblast-like cells (Kuckenberg et al., 2010; Nishioka et al., 2009; Niwa et al., 2005), but although the resultant cells upregulate key trophoblast marker genes, the levels remain far lower than the expression levels in mTSCs derived from the blastocyst and colony morphology cannot be maintained (Cambuli et al., 2014). Transient ectopic expression of a combination of the TE-associated transcription factors *Gata3*, *Eomes* and *Tcfap2c*, with either *Ets2* or *Myc*, has been shown to induce TSC fate in mouse fibroblasts (Benchetrit et al., 2015; Kubaczka et al., 2015). These alternatively derived cells recapitulate the morphology, epigenomes, and gene expression patterns and levels of blastocyst-derived TSCs and contribute exclusively to the placenta following blastocyst injection.

Here, we explored time-course RNA-sequencing (RNA-seq) analysis of human TE development to identify putative transdifferentiation transcription factors. We identified and confirmed enriched expression of four transcription factors (*GATA2*, *GATA3*, *TFAP2C* and *KLF5*) in the TE lineage of human blastocysts. Similar to strategies used in the mouse, we demonstrate using both a doxycycline-inducible approach and modified mRNA transfection that the overexpression of these factors transdifferentiates primed hESCs to iTSCs. iTSCs can be maintained stably in culture and recapitulate hTSC and trophoblast marker expression (Lee et al., 2016; Okae et al., 2018). RNA-seq analysis reveals that iTSCs are transcriptionally similar to existing hTSCs and primary CTB cells. When subjected to directed differentiation protocols, iTSCs successfully yield both STBs and EVT-like cells. Altogether, this work reveals a novel strategy for the establishment of iTSCs and significantly expands the repertoire of cells to model placenta biology *in vitro*.

## RESULTS

### Identification and validation of candidate transcription factors

**Fig. 1.**
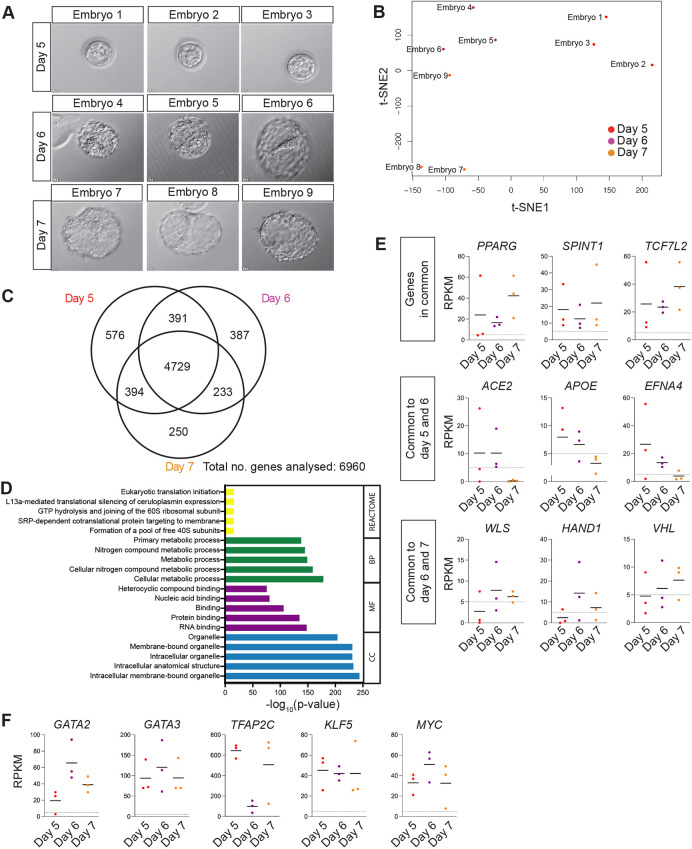
**Transcriptome analysis of TE in human blastocysts.** (A) Brightfield images of human blastocysts microdissected for TE isolation ahead of RNA-sequencing analysis. (B) t-distributed stochastic neighbour embedding (t-SNE) plot based on VST-normalisation of the top 6000 most variable expressed genes. A colour-coded sample key is provided. (C) Venn diagram indicating the number of transcripts with overlap or unique expression at day 5, day 6 and day 7 in the mural TE (RPKM>5). (D) The genes identified as expressed in common for day 5, 6 and 7 were used to perform Gene Ontology and REACTOME functional enrichment analyses; the most enriched terms for the categories biological process (BP), cellular component (CC), and molecular function (MF) as well as REACTOME pathways are shown. (E) Scatterplots showing selected genes that are expressed in common at all stages analysed, expressed in common at days 5 and 6, or expressed in common at days 6 and 7. Horizontal line denotes the mean RPKM value of each time point; dotted line shows RPKM value=5. (F) Scatterplots showing expression of *GATA2*, *GATA3*, *TFAP2C*, *KLF5* and *MYC* at days 5, 6 and 7. Horizontal line denotes the mean RPKM value of each time point; dotted line shows RPKM value=5.

In this study, we sought to devise a transdifferentiation strategy to generate iTSCs informed by the expression of transcription factors that are enriched in the TE. We initially sought to determine which genes overlap in their expression in the TE across pre-implantation stages of human development, reasoning that commonly expressed transcripts may be required for the establishment and maintenance of the TE and thereby may facilitate iTSC transdifferentiation. We cultured human embryos from embryonic day 5, when the blastocyst is first established and TE cells are discernible, until embryonic day 7, which is the latest stage we can culture pre-implantation blastocysts to *in vitro* (Fig. 1A). We performed RNA-seq analysis to determine the gene expression profile for each developmental stage (Table S1). Bulk RNA-seq analysis allowed us to identify lowly expressed genes, including transcription factors, which often drop out from single cell RNA-seq data (Kharchenko et al., 2014), and had the additional benefit of minimising the numbers of embryos used in this study. We did not observe expression of molecular markers associated with the epiblast (EPI) or primitive endoderm (PE), such as *NANOG* or *SOX17*, in TE samples, indicating that we did not have contamination from inner cell mass (ICM) cells (Table S1). t-distributed stochastic neighbour embedding (t-SNE) dimensionality reduction analysis indicated that plotting the first two dimensions of the t-SNE separates TE samples into three groups corresponding to the developmental time points analysed (Fig. 1B). This was confirmed by a principal component analysis (PCA), which showed that when the first five principal components (PCs) are plotted against each other, PC1 separates TE samples into the three developmental time points (Fig. S1A). We compared the global gene expression patterns at these stages of development to identify the transcripts that are commonly expressed and those unique to each stage (Table S2). We found that most transcripts, 4729 genes out of a total of 6960 analysed, were expressed in common at the stages analysed compared with 576, 387 and 250 genes uniquely expressed at day 5, 6 and 7, respectively (Fig. 1C). This suggests that, once initiated, the TE programme remains transcriptionally consistent across pre-implantation development. Functional enrichment analysis using Gene Ontology (Ashburner et al., 2000; Carbon et al., 2021; Mi et al., 2019) and Reactome databases (Jassal et al., 2020) revealed enrichment in metabolic processes, translational activities and molecule binding, which is supportive of the rapid expansion of the TE (Fig. 1D). Upon further inspection of the gene lists associated with these terms, we found an enrichment in genes that are required for efficient oxidative phosphorylation, including genes encoding complexes of the electron transport chain [*MT-ND1*, *SDHD*, *CYC1*, *UQCRQ, UQCRC1*, *ATP5E* (*ATP5F1E*), *ATP5I* (*ATP5IF1*) and *ATP5L* (*ATP5MG*)]. In addition, we observed an enrichment of genes involved in mitochondrial morphology. Interestingly, although we noted enrichment of genes regulating mitochondrial fusion (*MFN1* and *OPA1*), we did not detect expression of genes regulating mitochondrial fission (*DNM1L*). In all, this suggests that mitochondrial fusion in the TE may be essential for the electron transport chain, favouring oxidative phosphorylation. This is in agreement with the observation that oxidative phosphorylation activity increases in developing mammalian blastocysts and correlates with the capacity of the embryo to develop to term following implantation (Gardner et al., 1998; Houghton, 2006; Leese et al., 2008). The genes expressed in common included *PPARG*, which is expressed in CTBs and regulates differentiation (Murthi et al., 2013), the CTB marker *SPINT1* (Kohama et al., 2012) and *TCF7L1*, a key transcription factor of the WNT signalling pathway that is implicated in trophoblast proliferation (Meinhardt et al., 2014) (Fig. 1E). *ACE2*, *APOE* and the receptor protein-tyrosine kinase *EFNA4* were detected at the earlier time points analysed, whereas *WLS*, a core component of the WNT secretion pathway (Yu et al., 2014), *VHL*, a regulator of placenta vasculogenesis (Genbacev et al., 2001), and *HAND1*, a transcription factor involved in branching morphogenesis during mouse placentation (Riley et al., 1998), were enriched at later time points.

We collected a comprehensive transcription factor annotation from the Human Transcription Factor Database (HumanTFDB; http://bioinfo.life.hust.edu.cn/HumanTFDB#!/; Hu et al., 2019) and cross-referenced this against the list of transcripts detected in common at all stages analysed. This identified 232 transcription factors expressed in the TE (Table S3). We similarly mined our previously published single-cell RNA-seq datasets to identify transcription factors that were significantly enriched in the TE compared with the EPI and PE cells (Blakeley et al., 2015). We further refined these lists of TE-associated transcription factors by screening for transcription factors with a known role in mouse TE development (Kuckenberg et al., 2010; Ralston et al., 2010) or a suggested role in the human placenta. In all, from these analyses we identified four transcription factors for further investigation as candidate transdifferentiation factors: *GATA2*, *GATA3*, *TFAP2C* and *KLF5* (Fig. 1F, Table S4).

From our analysis of single-cell RNA-seq data using our previously published Shiny App programme (Blakeley et al., 2015; Wamaitha et al., 2020), we found that *GATA2*, *GATA3* and *TFAP2C* share a common pattern of expression, with an onset of embryonic transcription shortly prior to TE initiation at the 8-cell stage (Gerri et al., 2020) and high expression maintained as development proceeds. By contrast, *KLF5* is expressed in human zygotes and its expression increases from the 8-cell stage. Analysis of lineage-specific gene expression patterns has shown that *GATA2* and *GATA3* are enriched specifically in the TE (Home et al., 2009, 2017). Although *TFAP2C* is detected in the TE, it is also detected in the EPI, which we previously confirmed at the protein level (Blakeley et al., 2015). *KLF5* is detected in all three lineages, with the most abundant expression in the TE (Blakeley et al., 2015).

**Fig. 2.**
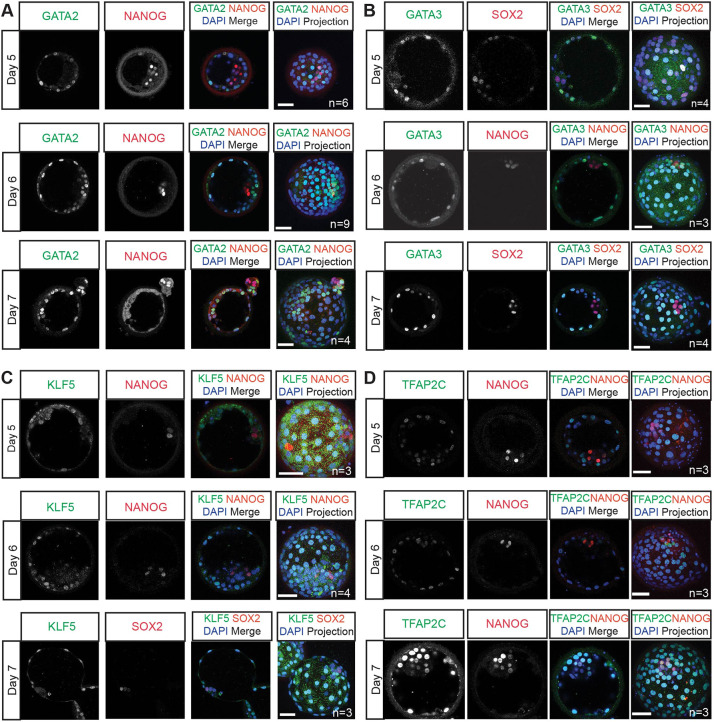
**Identification of TE-associated transcription factors in the human blastocyst.** (A-D) Representative images of immunofluorescence analysis of GATA2 (A), GATA3 (B), KLF5 (C) and TFAP2C (D) (green) and DAPI nuclear staining (blue) in human blastocysts cultured to day 6 post-fertilisation. ICM cells are detected by either the expression of NANOG or SOX2 (red). *n*=number of embryos analysed. Scale bars: 50 µm.

We next performed immunofluorescence analysis of these transcription factors in human embryos cultured from embryonic day 5 to 7. At all stages analysed, GATA2 protein expression was detected in the nuclei in TE cells, which were identified by both their position within the blastocyst and the absence of NANOG expression (Fig. 2A). Similarly, GATA3 protein was detected in TE cells across the stages analysed (Fig. 2B). The pattern of GATA2 and GATA3 expression recapitulates what has been reported in mouse embryos (Home et al., 2017) and is consistent with our previous analysis of GATA3 expression (Gerri et al., 2020). At all stages analysed, nuclear KLF5 was detected in the TE, whereas the ICM cells showed some cytoplasmic expression (Fig. 2C). This pattern is similar to what has been described in the mouse, where KLF5 is expressed in all cells of pre-implantation embryos with lower expression in the ICM compared with the TE (Lin et al., 2010). By contrast, TFAP2C was detected at similar levels in both TE cells and NANOG-positive EPI cells throughout all the blastocyst stages analysed. As we, and others, reported previously, this contrasts with the expression pattern in the mouse where the homologue TCFAP2C is exclusively expressed in the TE (Fig. 2D) (Blakeley et al., 2015; Kuckenberg et al., 2010). Altogether, the enriched expression of GATA2, GATA3, TFAP2C and KLF5 in the TE suggests that these transcription factors may have a functional role within the TE transcriptional network.

### Generation of 5F-iTSCs from hESCs using a lentiviral doxycycline-inducible system

**Fig. 3.**
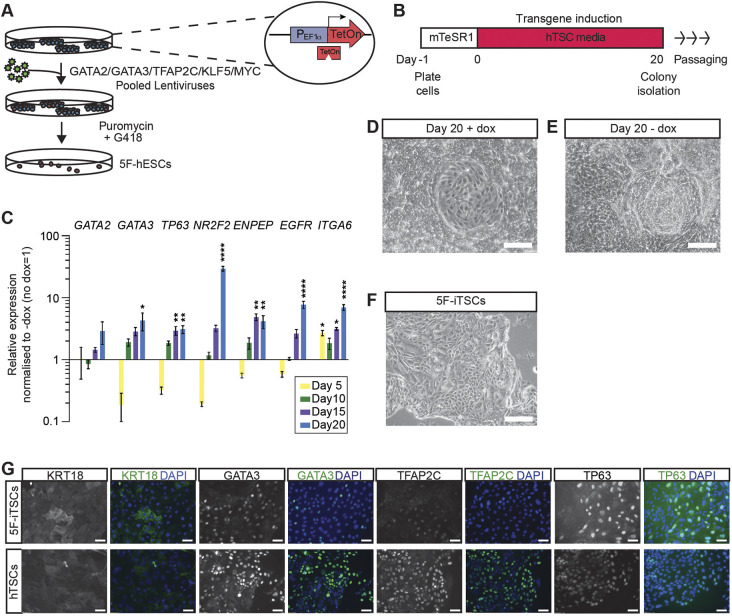
**Induced expression of GATA2, GATA3, TFAP2C and KLF5 for 20 days programs hESCs to hTSC-like cells.** (A) Schematic of the generation of GATA2, GATA3, TFAP2C, KLF5 and MYC inducible hESCs by lentiviral transduction. (B) Schematic of the strategy for transdifferentiation of 5F-hESCs to hTSCs. 5F-hESCs were plated in mTeSR1 media for 24 h, after which media was replaced with hTSC media. Doxycycline was administered daily for 20 days in hTSC media for transgene induction. (C) Time-course RT-qPCR analysis for the detection of endogenous *GATA2*, *GATA3*, *TP63*, *NR2F2*, *ENPEP*, *EGFR* and *ITGA6* in 5F-hESCs across 20 days of doxycycline induction in hTSC media. Relative expression is reflected as fold change over uninduced 5F-hESCs cultured in hTSC media normalized to *GAPDH*. Data are mean±s.e.m. of *n*=3 biological replicates analysed with a one-way ANOVA with Dunnett's post-hoc test (*P<0.05, **P<0.01, ****P<0.001). (D) Brightfield images of 5F-hESCs cultured in hESCs cultured in hTSC media in the presence of doxycycline for 20 days (+ dox). (E) Brightfield images of 5F-hESCs cultured in hTSC media in the absence of doxycycline for 20 days (− dox). (F) Brightfield images of stable 5F-iTSC lines derived from transgene overexpression grown for 15 passages. (G) Immunofluorescence analysis for the detection of KRT18, GATA3, TFAP2C and TP63 (green) and DAPI nuclear staining (blue) in stable 5F-iTSCs and previously established control hTSCs. Scale bars: 200 µm (D-F); 50 µm (G).

We next evaluated whether the expression of these transcription factors was sufficient to facilitate the transdifferentiation of hESCs cultured in primed conditions directly to iTSCs without a requirement to start from established naïve hESCs. We engineered doxycycline-inducible hESCs and confirmed overexpression of exogenous *GATA2*, *GATA3*, *TFAP2C*, *KLF5* and *MYC* (Fig. 3A, Fig. S1B,C). We also induced the expression of *MYC* because we identified high expression of the transcript across the stages of TE development analysed (Table S4), and it has been shown to enhance the efficiency of cellular reprogramming in other contexts (Nakagawa et al., 2008). These cells are henceforth referred to as five-factor (5F) hESCs (5F-hESCs).

To initiate the transdifferentiation process, transgenes were induced by exposing 5F-hESCs to doxycycline in the presence of hTSC media for 20 days (Fig. 3B). This duration of transgene expression has been previously described for transcription factor-based transdifferentiation of mouse fibroblasts to TSCs (Benchetrit et al., 2015). To gain insight into the effect of 5F induction during this time period, we performed a time-course quantitative RT-PCR (RT-qPCR) analysis for the detection of endogenous trophoblast-associated transcripts in induced 5F-hESCs compared with uninduced controls. *TP63*, *ENPEP* and *ITGA6* expression was significantly upregulated after 15 days of transgene induction, whereas *GATA3*, *NR2F2* and *EGFR* were subsequently significantly upregulated by day 20 in comparison with uninduced 5F-hESCs cultured in hTSC media alone (Fig. 3C, Fig. S1D). These observations validate the duration of transgene induction for transdifferentiation. After 20 days, epithelial colonies with polygonal hTSC-like morphology were apparent within a heterogenous population as is similar to induced pluripotent stem cell (iPSC) derivation and naïve programmming of hTSCs (Liu et al., 2020; Takahashi et al., 2007) (+dox; Fig. 3D). As a control, 5F-hESCs were cultured in hTSC media in the absence of doxycycline. In these conditions wide-spread cell proliferation and differentiation was observed (−dox; Fig. 3E). Colonies were manually selected and expanded upon subsequent passages. Colonies continued to maintain their morphology and proliferate in the absence of doxycycline. These cells are henceforth referred to as 5F-iTSCs (Fig. 3F). Stable 5F-iTSC lines were generated and maintained in culture for over 20 passages. By contrast, uninduced 5F-hESCs did not survive the first passage and underwent cell death. Immunofluorescence analysis of stable 5F-iTSCs indicated widespread expression of TE-associated keratin, KRT18, GATA3, TFAP2C and TP63, similar to existing hTSCs (Fig. 3G). By contrast, 5F-hESCs cultured in conventional primed conditions only showed some upregulation of KRT18 (Fig. S1D).

### Generation of 5F-iTSCs from hESCs using non-integrating modified mRNAs

**Fig. 4.**
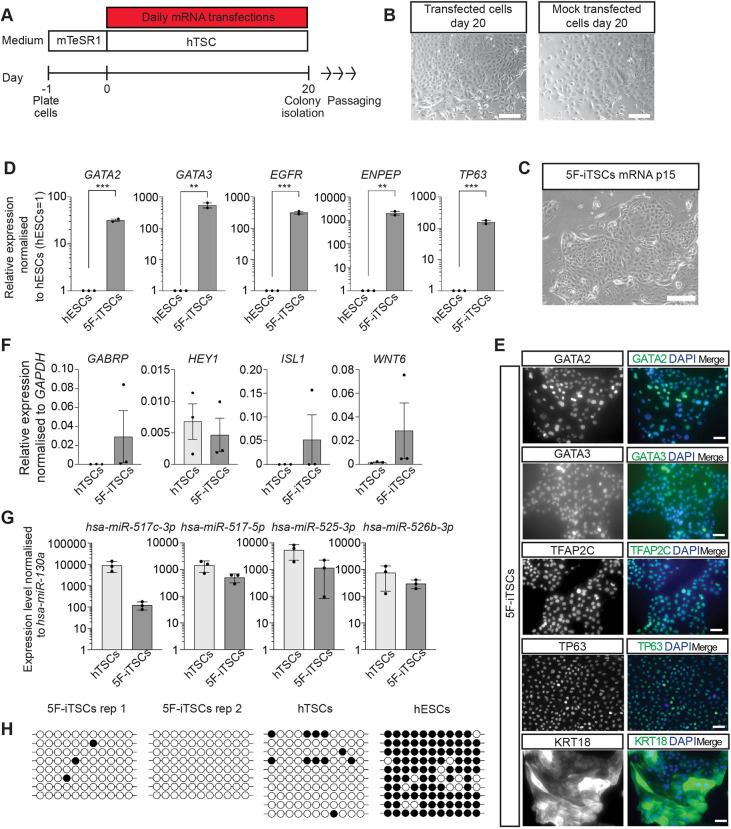
**Characterisation of 5F-iTSCs programmed from primed hESCs using modified mRNAs.** (A) Schematic of the strategy for transdifferentiation of hESCs to 5F-iTSCs using modified mRNAs. hESCs were plated in mTeSR1 media for 24 h after which media was replaced with hTSC media. mRNAs encoding *GATA2*, *GATA3*, *TFAP2C*, *KLF5* and *MYC* were administered daily for 20 days by lipofection-mediated transfection. (B) Brightfield images of 5F mRNA-cocktail transfected hESCs and mock-transfected cells on day 20. (C) Brightfield image of stable 5F-iTSCs derived from mRNA transdifferentiation grown for 15 passages. (D) RT-qPCR analysis for the detection of *GATA2*, *GATA3*, *ENPEP*, *EGFR* and *TP63* in stable 5F-iTSCs. Relative expression is reflected as fold change over hESCs cultured in mTeSR media normalised to *GAPDH.* Data are mean±s.e.m. *n*=2-3 biological replicates analysed with an unpaired one-tailed *t*-test (**P<0.01, ***P<0.005). (E) Immunofluorescence analysis for the detection of the selected trophoblast markers GATA2, GATA3, TFAP2C, TP63 and KRT18 (green) and DAPI nuclear staining (blue) in stable 5F-iTSCs. (F) RT-qPCR for the detection of the amnion-associated transcripts *GABRP*, *HEY1*, *ISL1* and *WNT6* in existing hTSCs and 5F-iTSCs. Relative expression is shown as ΔΔCt values normalised to *GADPH*. Data are mean±s.e.m. *n*=3 biological replicates analysed with an unpaired two-tailed *t*-test (not significant). (G) RT-qPCR for the detection of C19MC miRNAs in established hTSCs and 5F-iTSCs. Expression is normalised to *hsa-miR-130a*. Data are mean±s.e.m. *n*=3 biological replicates analysed with an unpaired two-tailed *t*-test (not significant). (H) Bisulphite sequencing analysis of the *ELF5* promoter region. Filled circles indicate methylated cytosine residues. *ELF5* promoter is hypomethylated in 5F-iTSCs (*n*=2 biological replicates) and hTSCs and methylated in hESCs. Scale bars: 200 µm (B,C); 50 µm (E).

Reprogramming via lentiviral transduction is reportedly an inefficient process (ranging from 0.01% to 0.1%) (Wernig et al., 2008) and integrated constructs can be spontaneously silenced and reactivated during cell culture and differentiation (Ellis, 2005; Herbst et al., 2012). As an alternative approach to lentiviral transduction, we employed a strategy of transcription factor overexpression using chemically modified mRNAs, which has been used previously to generate transgene-free human iPSCs (Mandal and Rossi, 2013; Warren et al., 2010). Individual mRNAs encoding GATA2, GATA3, TFAP2C, KLF5 and MYC were synthesised by *in vitro* transcription. Uridine and cytidine were substituted with the modified nucleotides pseudo-uridine and 5′methylcytidine to prevent cellular immune responses, and mRNA was capped with a modified 5′guanine cap to improve mRNA half-life. A cocktail of mRNA was made by pooling individual mRNAs in equal molar ratios and this was delivered into hESCs by lipofection every day for 20 days (Fig. 4A). Similar to what was observed with the doxycycline-inducible system, transfected cells underwent a morphological change towards a polygonal shape within distinct colonies, whereas mock-transfected cells underwent widespread differentiation (Fig. 4B). After 20 days of transfection, colonies were manually selected and stable iTSC lines were generated that could be maintained in culture for over 20 passages (Fig. 4C). We next performed an in-depth analysis of mRNA-generated 5F-iTSCs. RT-qPCR analysis of 5F-iTSCs confirmed significant upregulation of *GATA2*, *GATA3*, *EGFR*, *ENPEP* and *TP63* compared with the starting population of hESCs (Fig. 4D). Immunofluorescence analysis of 5F-iTSCs confirmed widespread expression of trophoblast markers GATA2, GATA3, TFAP2C, TP63 and KRT18 (Fig. 4E). Previous studies have shown that strategies to derive TSCs from primed hESCs using growth factor modulation instead yields amnion-like cells (Guo et al., 2021; Io et al., 2021; Ohgushi et al., 2022). Here, we performed RT-qPCR analysis for the detection of the amnion-associated transcripts *GABRP*, *HEY1*, *ISL1* and *WNT6*. Although we detected low levels of expression of these transcripts, we found that levels of expression were equivalent to those detected in established hTSCs derived from blastocyst and placenta (Fig. 4F). RT-qPCR analysis of expression of the trophoblast-specific chromosome 19 microRNA (miRNA) cluster confirmed that 5F-iTSCs express miRNAs at levels comparable to established hTSCs (Fig. 4G). Methylation analysis of the *ELF5* promoter showed hypomethylation of the region in 5F-iTSCs and hTSCs, whereas the starting population of hESCs displayed hypermethylation (Fig. 4H). Altogether, these data suggest that the 5F-iTSCs exhibit key characteristics of bona fide hTSCs (Lee et al., 2016).

### 5F-iTSCs are transcriptionally similar to previously established hTSCs and primary CTBs

**Fig. 5.**
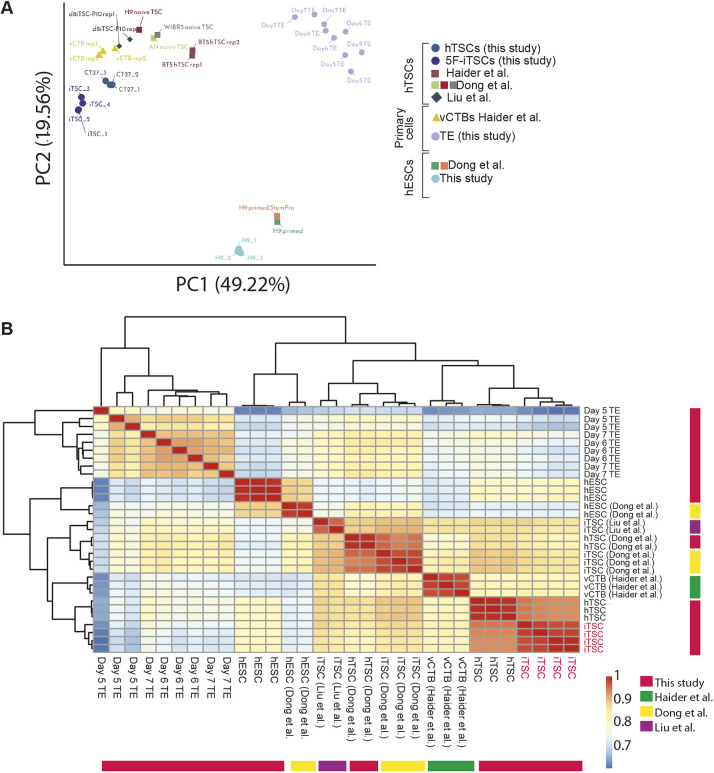
**Global transcriptional analysis of 5F-iTSCs and similarity to previously established TSCs.** Analysis of mRNA-derived 5F-iTSCs (this study), previously established hTSCs (CT27, BTS5) (Okae et al., 2018), iTSCs derived from naïve transdifferentiation experiments (Liu et al., 2020; Dong et al., 2020), primary placental CTBs (Haider et al., 2018) and primed hESCs (this study) (Dong et al., 2020). (A) PCA using the top 500 most variable expressed genes. (B) Unsupervised hierarchical clustering of the samples. Spearman's rank correlation coefficient was plotted on a high-to-low scale (red-yellow-blue). 5F-iTSCs generated in this study are labelled in red on the outer bars.

We next compared global gene expression of mRNA-generated 5F-iTSCs to alternatively derived TSCs by integrating previously published datasets (Dong et al., 2020; Liu et al., 2020; Okae et al., 2018) as well as TE samples generated in this study, primary CTB cells (Haider et al., 2018) and primed hESCs (Dong et al., 2020). After adjusting for batch effects, we used the top 500 most variably expressed genes to perform dimensionality reduction analysis. A PCA plotting PC1 against PC2 separated samples into three groups representing TE, hESCs and iTSCs together with previously established hTSCs and primary CTBs. PC1 accounted for 49.22% of the variance (Fig. 5A). To determine which samples were most similar with respect to genes with significant expression changes, we performed unsupervised hierarchical clustering. Consistent with the PCA, three major clusters were observed: TE; hESCs; and 5F-iTSCs, *in vitro* hTSCs and primary CTBs. Significantly, this analysis showed that the 5F-iTSCs generated in this study were most closely related to hTSCs generated by Okae et al. (2018), followed by primary CTBs (Fig. 5B). We confirmed that 5F-iTSCs and all hTSCs analysed are transcriptionally similar to CTBs and distinct from the TE, suggesting that further refinement of hTSC medium is required to capture a developmentally earlier state. Together, these findings indicate that our 5F-iTSCs share a common transcriptomic profile with existing hTSCs and their *in vivo* cellular counterparts.

### 5F-iTSCs differentiate into both STBs and EVTs

**Fig. 6.**
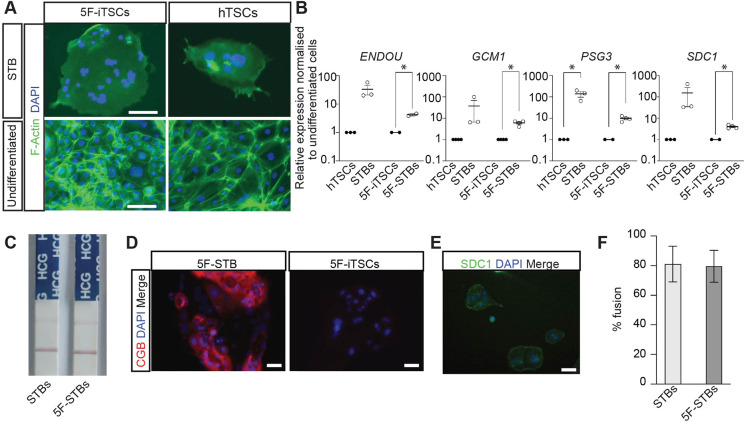
**5F-iTSCs can be directed to differentiate to STB.** (A) Immunostaining analysis of F-actin (green) and DAPI nuclear staining (blue) in STB-differentiated and undifferentiated 5F-iTSCs and hTSCs. (B) RT-qPCR analysis for the detection of STB markers *ENDOU*, *GCM1*, *PSG3* and *SDC1* in STBs differentiated from control hTSCs (STBs) and 5F-iTSCs (5F-STBs). Relative expression is shown as fold change over the undifferentiated starting cell population normalised to *GAPDH* (undifferentiated cells=1). Data are mean±s.e.m. of *n*=2-3 biological replicates analysed with a multiple *t*-test (*P<0.05). (C) Detection of hCG in spent culture media from control STBs and 5F-STBs using an over-the-counter pregnancy test. Data are representative images of *n*=3 biological replicates. (D) Immunofluorescence analysis for the detection of CGB (red) and DAPI nuclear staining (blue) in 5F-STBs and undifferentiated 5F-iTSCs. (E) Immunofluorescence analysis for the detection of SDC1 (green) and DAPI nuclear staining (blue) in 5F-STBs. (F) Fusion efficiency of control STBs (light grey bars) and 5F-STBs (dark grey bars). Data are mean±s.e.m. and analysed with an unpaired two-tailed *t*-test (n.s). Scale bars: 50 µm.

We next examined the differentiation capacity of 5F-iTSCs. We utilised a method to generate terminally differentiated STB-like cells as previously described (Okae et al., 2018). Accordingly, in both 5F-iTSCs and existing hTSCs, we observed formation of multinucleated syncytia, which exhibited a reduction in filamentous actin (F-actin) expression, indicating reorganisation of the actin cytoskeleton (Fig. 6A). RT-qPCR of STB-like cells generated from 5F-iTSCs showed significant upregulation of STB-associated genes, *ENDOU*, *GCM1*, *PSG3* and *SDC1*, compared with undifferentiated 5F-iTSCs, as observed in STBs differentiated from control hTSCs (Fig. 6B). Human chorionic gonadotropin (hCG) is one of the first peptide hormones that is produced by the STB (Kliman et al., 1986). Spent culture media from the iTSC-derived STB-like cells was collected after 6 days of differentiation and subjected to an over-the-counter pregnancy test kit, which showed detectable hCG expression, similar to STBs derived from hTSCs (Fig. 6C). Production of hCG protein was confirmed by immunofluorescence analysis, which detected expression of the β subunit of hCG in 5F-STBs and not in undifferentiated 5F-iTSCs (Fig. 6D). Immunofluorescence analysis confirmed protein expression of SDC1 in 5F STB-like cells (Fig. 6E). The fusion index revealed that approximately 80% of the 5F-iTSCs nuclei were contained within syncytia, comparable to what was observed with hTSCs (Fig. 6F).

**Fig. 7.**
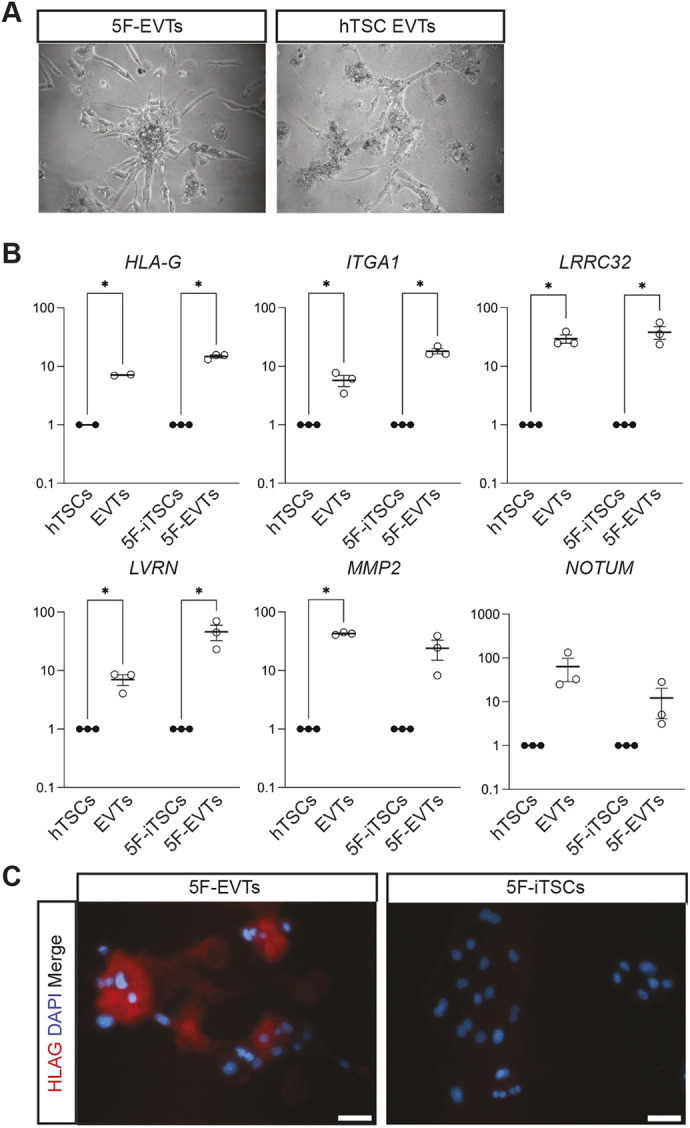
**5F-iTSCs can be directed to differentiate to extravillous trophoblast.** (A) Brightfield imaging of EVTs differentiated from 5F-iTSCs and hTSCs. (B) RT-qPCR analysis for the detection of the selected EVT markers *HLA-G*, *ITGA1*, *LRRC32*, *LVRN*, *MMP2* and *NOTUM*. Relative expression is shown as fold change over undifferentiated 5F-iTSCs normalised to *GAPDH*. Data are mean±s.e.m. of *n*=3 biological replicates analysed with an unpaired one-tailed *t*-test (*P<0.05). (C) Immunofluorescence analysis for the detection of HLA-G (red) and DAPI nuclear staining (blue) in 5F-EVTs and 5F-iTSCs. Scale bars: 50 μm.

5F-iTSCs were also differentiated into EVT-like cells using previously published protocols (Okae et al., 2018). After 6 days, we observed morphological changes whereby cells acquired an elongated spindle-like appearance reminiscent of EVTs (Fig. 7A). RT-qPCR analysis of EVT-like cells revealed significant upregulation of the EVT-associated transcripts *HLA-G*, *ITGA1*, *LRRC32*, *LVRN* and *MMP2* compared with undifferentiated 5F-iTSCs, similar to what was observed in EVTs differentiated from hTSCs (Fig. 7B). HLA-G is a marker of EVTs throughout all stages of gestation, and we confirmed its specific expression in 5F-EVTs (Fig. 7C). Altogether, these data indicate that 5F-iTSCs have the capacity for differentiation to both STBs and EVTs, and therefore exhibit all hallmarks of hTSCs (Lee et al., 2016; Okae et al., 2018).

### Narrowing down transcription factors essential to generate iTSCs

To determine which transcription factors are required for the induction of iTSCs, we examined the effect of withdrawing individual transcription factors from the pool of factors on the formation of 5F-iTSCs. *MYC* was kept constant, reasoning that, based on its reprogramming effect in other contexts, it functions to generally enhance reprogramming/transdifferentiation rather than being essential for iTSC generation (Nakagawa et al., 2010). To allow us to directly compare the success and efficiency of the iTSC programme induction, we maintained an equivalent total amount mRNA in each cocktail by replacing the omitted factors with an equivalent amount of mRNA encoding GFP.

**Fig. 8.**
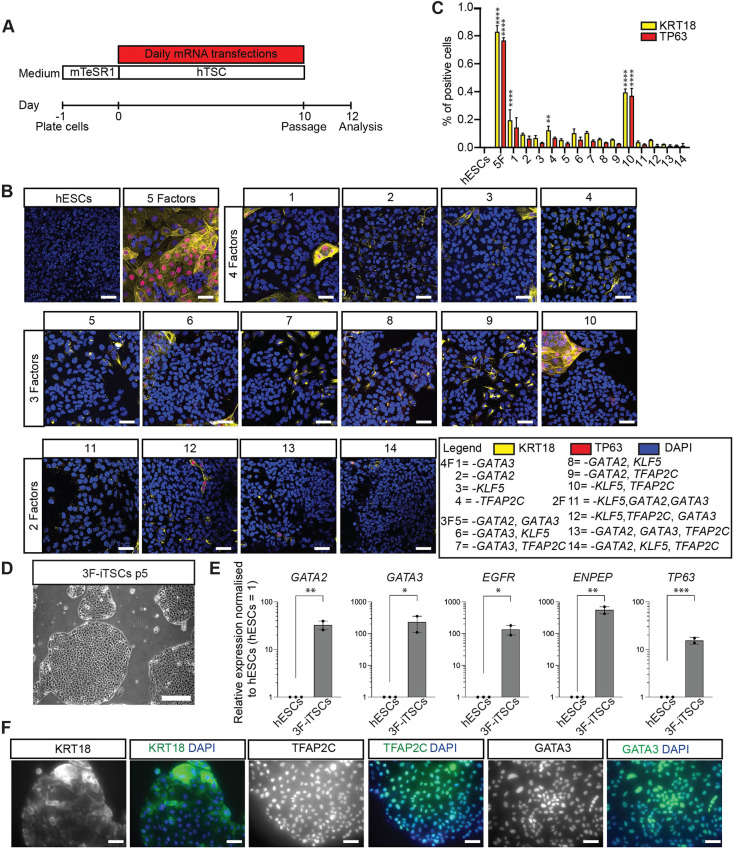
**GATA2 and GATA3 are required and sufficient for induction of the hTSC programme.** (A) Schematic of the strategy for testing factors required for iTSC transdifferentiation. hESCs were transfected with cocktails of modified mRNAs in which omitted factors were replaced with equivalent amount of mRNA encoding GFP to keep the molar ratio the same. Cells were transfected daily for 10 days, at which point they were fixed for immunofluorescence analysis. (B) Immunofluorescence analysis for the detection of TP63 (red) and KRT18 (yellow) and DAPI nuclear staining (blue) in transfected cells. Merged images are shown. Key for mRNA cocktail combinations: 1, *GATA2*, *TFAP2C*, *KLF5* and *MYC* (*GATA3* omitted); 2, *GATA3*, *TFAP2C*, *KLF5* and *MYC* (*GATA2* omitted); 3, *GATA2*, GAT*A3*, *TFAP2C* and *MYC* (*KLF5* omitted); 4, *GATA2*, *GATA3*, *KLF5* and *MYC* (*TFAP2C* omitted); 5, *TFAP2C*, *KLF5* and *MYC* (*GATA2* and *GATA3* omitted); 6, *GATA2*, *TFAP2C* and *MYC* (*GATA3* and *KLF5* omitted); 7, *GATA2*, *KLF5* and *MYC* (*GATA3* and *TFAP2C* omitted); 8, *GATA3*, *TFAP2C* and *MYC* (*GATA2* and *KLF5* omitted); 9, *GATA3*, *KLF5* and *MYC* (*GATA2* and *TFAP2C* omitted); 10, *GATA2*, *GATA3* and *MYC* (*TFAP2C* and *KLF5* omitted); 11, *TFAP2C* and *MYC* (*KLF5*, *GATA2* and *GATA3* omitted); 12, *GATA2* and *MYC* (*GATA3*, *TFAP2C* and *KLF5*); 13, *KLF5* and *MYC* (*GATA2*, *GATA3* and *TFAP2C* omitted); 14, *GATA3* and *MYC* (*GATA2*, *KLF5* and *TFAP2C* omitted). (C) Quantification of KRT18- and TP63-positive cells. Data are mean±s.e.m. analysed with an unpaired *t*-test (*P<0.05, **P<0.01, ****P<0.001). (D) Brightfield imaging of the 3F-iTSC line derived and maintained in culture for five passages. (E) RT-qPCR analysis for the detection of *GATA2*, *GATA3*, *ENPEP*, *EGFR* and *TP63* in 3F-iTSCs. Relative expression is reflected as fold change over hESCs cultured in mTeSR media normalized to *GAPDH.* Data are mean±s.e.m., *n*=2-3 biological replicates analysed with an unpaired one-tailed *t*-test (*P<0.05, **P<0.01, ***P<0.005). (F) Immunofluorescence analysis for the detection of the selected trophoblast markers KRT18, TFAP2C and GATA3 (green) and DAPI nuclear staining (blue) in 3F-iTSCs. Scale bars: 50 µm.

After 10 days of lipofection, immunofluorescence analysis was performed for the expression of two markers of hTSCs: KRT18 and TP63 (Fig. 8A,B, Fig. S1E) (Meistermann et al., 2021). Induction of the iTSC programme was first determined by the presence of cells co-expressing nuclear TP63 and filamentous KRT18 expression extending from the nucleus to the cell membrane, as we observed in cells treated with the 5F cocktail (Fig. 8B, Fig. S2). Immunofluorescence analysis for the detection of the additional markers TEAD4 and KRT7 further confirmed the identity of 5F-iTSCs (Fig. S3A). Quantification of immunofluorescence analyses revealed that approximately 80% of cells were KRT18 or TP63 positive when treated with the 5F cocktail (Fig. 8C). We observed that only the combination of *GATA2*, *GATA3* and *MYC* produced iTSC colonies, albeit at lower proportions compared with the 5F cocktail, with approximately 35% of cells showing co-expression of TP63 and KRT18 (Fig. 8B,10; Fig. 8C). This three-factor (3F) combination led to expression of KRT7 and nuclear TEAD4 (Fig. S3K, yellow arrowheads), similar to those generated in the control 5F combination (Fig. S3A). By contrast, although the other conditions showed some upregulation of KRT7 and KRT18, the expression pattern of the latter was patchy and disorganised, and the cells did not co-express TP63 or exhibit nuclear TEAD4 (Fig. 8B, Fig. S3). Next, we sought to determine whether stable iTSCs could be generated from these three factors. We transfected hESCs for 20 days with the *GATA2*, *GATA3* and *MYC* cocktail and observed appearance of hTSC-like colonies, similar to the 5F transdifferentiation. These colonies were manually selected and a homogenous 3F-iTSC line was successfully generated (Fig. 8D). RT-qPCR analysis revealed significant upregulation of the markers *GATA2*, *GATA3*, *EGFR*, *ENPEP* and *TP63* in 3F-iTSCs compared with the hESC starting population (Fig. 8E). Immunofluorescence confirmed widespread expression of KRT18, TFAP2C and GATA3 (Fig. 8F). However, these 3F-iTSCs could not be maintained long-term in culture, with cell degeneration, loss of colony integrity and loss of proliferation apparent after five passages. This suggests that *GATA2* and *GATA3* are required for induction of the trophoblast network, but additional factors are required to maintain the cells in long-term culture.

## DISCUSSION

Defective TE specification, trophoblast differentiation and maturation leading to abnormal placentation underlies miscarriage and pre-eclampsia (Burton and Jauniaux, 2017; Fisher, 2015). hTSCs present a paradigm for modelling placenta development and disease *in vitro*. However, a caveat of existing cytotrophoblast- and blastocyst-derived hTSCs is that it cannot be ascertained whether the starting population of cells would have given rise to a normal or a disease-affected placenta (Okae et al., 2018). This provides the motivation for the development of strategies to generate hTSCs from alternative starting cell populations. In this study, we investigate the sufficiency of TE-associated transcription factors to transdifferentiate hESCs to iTSCs.

In the mouse, CDX2, GATA3, TCFAP2C and EOMES were identified as key regulators of the TE lineage (Kuckenberg et al., 2010; Ralston et al., 2010; Russ et al., 2000; Strumpf et al., 2005). These factors are highly enriched in mouse TE and mTSCs and have been shown to be capable of programming fibroblasts towards mouse iTSCs (Benchetrit et al., 2015; Kubaczka et al., 2015). In the human, overexpression of the trophoblast-associated factors *TFAP2C*, *TEAD4*, *CDX2*, *ELF5* and *ETS2* in term cytotrophoblast cells generates iTS-like cells. However, in this study the cells were not assessed for hallmarks of trophoblast and the cells remain distinct from the primary term CTB cells from which they were derived (Bai et al., 2021). Here, we identified GATA2, GATA3, TFAP2C and KLF5 as factors that are enriched in the human TE. Together with MYC, these transcription factors were capable of transdifferentiating primed hESCs to 5F-iTSCs that were transcriptionally equivalent to existing hTSCs. This is in contrast to what was observed in the mouse, where the overexpression of mouse TE-associated transcription factors was not able to transdifferentiate mouse ESCs into TSCs (Kubaczka et al., 2015).

In support of GATA3 as a candidate hTSC transdifferentiation factor is the recent work implicating GATA3 in the initiation of the TE lineage in human embryogenesis (Gerri et al., 2020). GATA2 is exclusively expressed in the TE of mouse, cow and human embryos, and regulates some trophoblast-related genes in mouse and cow (Bai et al., 2011; Gerri et al., 2020; Ma et al., 1997). The mouse homologue of *TFAP2C*, *Tcfap2c*, is required post-implantation, with knockout mice dying at embryonic day 7.5 as a result of proliferation and differentiation defects in the TE compartment (Auman et al., 2002; Werling and Schorle, 2002). In mouse TSCs, TCFAP2C is detected at the promoters of key genes, including *Elf5*, *Gata3*, *Hand1*, *Id2* and *Tead4* (Kidder and Palmer, 2010). By contrast, the role of *TFAP2C* in human trophoblast biology is not fully understood. It is known, however, that TFAP2C is a defining marker of CTBs across gestation (Lee et al., 2016) and it stimulates human placenta lactogen and hCG production (Richardson et al., 2001). Functional analysis of KLF5 in human TE and trophoblast has not yet been performed, but in the mouse this transcription factor is required for the establishment of the ICM and TE. *Klf5^−/−^* embryos arrest at the blastocyst stage owing to defects in TE development resulting in a failure to hatch (Lin et al., 2010). The expression patterns of these factors during human embryogenesis, together with their associated roles in TE and trophoblast biology, implicate *GATA2*, *GATA3*, *TFAP2C* and *KLF5* as candidate regulators of a human TE programme. Indeed, although GATA2, GATA3 and TFAP2C, along with TFAP2A, have been previously reported to differentiate hESCs to trophectoderm-like cells, these studies were not successful in establishing stable TSC lines (Krendl et al., 2017).

Previous studies have derived hTSC-like cells from different states of pluripotency. Primed pluripotent stem cells correspond to the EPI cells that diverge from the TE at the first lineage-specification event during embryogenesis. Naïve ESCs represent an earlier, less-fixed developmental state that reflect gene expression and epigenetic profiles similar to those of the early EPI or late morula (Takashima et al., 2014; Theunissen et al., 2014). Modulating the signalling environment of primed hESCs with BMP4 gives rise to a cell type that exhibits some characteristics of trophoblast, but the true identity of these cells is unclear (Bernardo et al., 2011; Roberts et al., 2018; Xu et al., 2002). More recently, it has been shown that primed hESCs treated with BMP4, A83-01 and PD173074 acquire amnion-like properties, in contrast to naïve cells, which in the same conditions generate hTSCs (Io et al., 2021). Another study demonstrated that although a 48 h pretreatment of primed hESCs with BMP4 before culturing in hTSC media generates hTSC-like cells, these cells cannot be maintained in culture or be directed to differentiate to STB and EVT (Kobayashi et al., 2022). Previous studies have shown that primed hESCs cultured in hTSC media had elevated cell death and lost proliferative capacity, consistent with our observations in this study (Castel et al., 2020; Cinkornpumin et al., 2020; Dong et al., 2020; Liu et al., 2020). Significantly, here we demonstrate the first successful generation of iTSCs from primed hESCs that exhibit transcriptional properties consistent with hTSCs and that can be maintained long-term in culture with bipotent differentiation capabilities.

Studies of naïve hESCs have shown that, when cultured in hTSC media, they could be converted into hTSCs (Castel et al., 2020; Cinkornpumin et al., 2020; Dong et al., 2020; Liu et al., 2020). Alternatively, naïve hESCs treated with either small molecule inhibitors of ERK/mitogen-activated protein kinase (MAPK) and Nodal signalling for 3-5 days (Guo et al., 2021), or for 3 days with transient addition of BMP4 and a JAK1 inhibitor (Io et al., 2021), differentiate to hTSCs when placed in conventional hTSC media. This is in contrast to primed hESCs, which, in the same conditions, generated amnion-like cells, leading to the hypothesis that primed hESCs have lost the developmental potential to make TE (Guo et al., 2021). Global transcriptome and chromatin landscape analysis of primed and naïve hESCs revealed that the latter more closely resembled hTSCs and exhibited open chromatin regions that are associated with the trophoblast lineage (Dong et al., 2020). Moreover, Guo and collaborators demonstrated that EPI cells from a day 6 blastocyst can give rise to TE-like cells when treated with the same conditions that generate hTSCs from naïve hESCs (Guo et al., 2021). This suggests that the human pluripotent state may have extended plasticity *in vivo*, with the pre-implantation EPI retaining the ability to contribute to the TE. This is in contrast to the mouse, in which the ICM loses TE potency after it has segregated into EPI and PE (Posfai et al., 2017).

A feature of naïve pluripotency is the expression of TFAP2C, which plays a crucial role during primed to naïve reversion by facilitating the opening of naïve-specific enhancers, as well as regulating the expression of the pluripotency factors OCT4 (also known as POU5F1) (Pastor et al., 2018) and KLF4 (Chen et al., 2018). Indeed, CRISPR/Cas9-mediated knockout of *TFAP2C* in naïve hESCs significantly affected the efficiency of transdifferentiation to hTSCs (Guo et al., 2021). This suggests a dual functionality of TFAP2C in human embryogenesis and stem cell counterparts in terms of maintaining naïvety and regulating the TE transcriptional network. Indeed, TFAP2C is suggested to both activate and repress target genes (Eloranta and Hurst, 2002; Pastor et al., 2018). Future molecular analysis of hTSCs revealing the TE-specific repertoire of TFAP2C gene targets may provide further insight into the molecular mechanisms underpinning the TE transcriptional network.

hTSC induction from a starting population of fibroblasts has recently been described. During reprogramming using forced overexpression of OCT4, SOX2, KLF4 and MYC (OSKM), a side-population of cells can be propagated as hTSC-like when transferred to hTSC culture conditions (Castel et al., 2020; Liu et al., 2020). Further investigation will be required to determine the identity of this side-population. It is unclear whether fibroblasts are required to transition through a state of pluripotency before acquiring trophoblast identity, or whether direct lineage conversion takes place. An alternative combination of factors GATA3, OCT4, KLF4 and MYC (GOKM) drives fibroblasts to hTSCs without this transition through the pluripotent state (Naama et al., 2023). Analysis of chromatin accessibility profiles during hTSC conversion found that, whereas OSKM activated loci shared by both hESCs and hTSCs, GOKM activated hTSC-specific loci. Moreover, transcriptional analysis found that iTSCs generated by OSKM reprogramming lacked expression of a class of genes involved in oestrogen response, which would likely be required for trophoblast function (Naama et al., 2023).

Currently, all *in vitro*-derived TSCs, including the 5F-iTSCs generated in this study, more closely resemble the post-implantation CTB cells rather than the pre-implantation TE. This suggests that the *in vitro* conditions currently used diverge from the pre-implantation *in vivo* niche. Elucidating signalling pathways active in the EPI during human embryogenesis has led to the refinement of more physiological culture conditions and established hESC lines more closely resembling their *in vivo* counterparts (Wamaitha et al., 2020). Further investigations of human TE and systematic evaluation of culture conditions may similarly lead to the derivation of TSC lines that more closely resemble this earlier TE developmental time point.

The combinatorial transdifferentiation experiments provided further insights in the roles of TE-associated transcription factors. Our 3F experiments suggest that both *GATA2* and *GATA3* are essential for upregulation of the hTSC programme. The GATA family of transcription factors are implicated in establishing cell fate during development and often show functional redundancy between members (Fujiwara et al., 2004; Peterkin et al., 2005). However, it is unclear whether GATA2 and GATA3 play different roles during transdifferentiation, or if there is functional redundancy between the two. Interestingly in our time-course analysis of transdifferentiation, the timing of onset of endogenous *GATA2*, as well as the levels to which it is upregulated, appear to lag that of *GATA3* expression. This may indicate that, although both are required, GATA3 acts upstream of GATA2. Future experiments, including epistatic functional studies and investigation of transcription factor binding and chromatin occupancy, to distinguish the roles of these factors would elucidate these mechanisms. Surprisingly, and in contrast to the mouse, *TFAP2C* appears dispensable for transdifferentiation. Although our transdifferentiation strategies were successful in establishing stable lines, in the future the efficiency of hTSC transdifferentiation may be further enhanced by either equivalently increasing the amount of each factor or by altering the stoichiometry of transcription factors, as has been demonstrated for iPSC and cardiomyocyte reprogramming (Carey et al., 2011; Muraoka and Ieda, 2015) The mRNA-based strategy would be advantageous in this context as it more easily allows for the generation of bespoke combinations of transcription factors. In addition, functional examination of these factors in knockout hTSCs may further refine the combination of essential factors required for the maintenance of these cell types.

There is a lack of understanding of the genetic basis of placental-related diseases and so we cannot currently apply CRISPR/Cas9-mediated genome editing to create mutations for disease modelling in existing hTSCs. Instead, we propose that the mRNA transdifferentiation strategy presented here could be applied and further refined to generate iTSCs from fibroblasts derived from individuals with placental-related disorders, or fibroblasts or mesenchymal stromal cells isolated from disease-affected placentas (Pelekanos et al., 2016). This strategy could facilitate the generation of individual-specific human TSCs. Indeed, we have attempted to apply the 5F strategy described here to fibroblasts, but our pilot studies were unsuccessful. This may suggest that additional transcription factors are required for fibroblasts to make the conversion. Indeed, Naama et al. have demonstrated that GATA3, in combination with classical reprogramming factors including OCT4, can induce hTSCs from fibroblasts (Naama et al., 2023). As OCT4 has been shown to be functionally required for human TE development (Fogarty et al., 2017), and is endogenously expressed in human ESCs (Reubinoff et al., 2000), we hypothesise that OCT4 may also be required alongside the five factors. Our application of mRNA transfection would have the benefit of controlling transcription factor stoichiometry, reducing variability of protein expression, and avoiding random integration of transgenes that can result in genomic modification and tumorigenicity (Warren et al., 2010). In all, this strategy could allow for the generation of a catalogue of clinically normal and disease-associated hTSCs that would provide a tool for basic research into trophoblast biology as well as a powerful tool for understanding placental defects, including recurrent miscarriage, pre-eclampsia, intrauterine growth restriction and stillbirth, as well as a future drug screening platform.

## MATERIALS AND METHODS

### Human embryo thaw and culture conditions

Human embryos that were surplus to family-building requirements were donated to The Francis Crick Institute for use in research projects under the UK Human Fertilisation and Embryology Authority License number R0162 and the Health Research Authority's Research Ethics Committee (Cambridge Central reference number 16/EE/0067). Slow-frozen blastocysts (day 5 and day 6) were thawed using the BlastThaw kit (Origio, 10542010A) following the manufacturer's instructions. Vitrified blastocysts (day 5 and day 6) were thawed using a vitrification thaw kit (Irvine Scientific, 90137-SO) following the manufacturer's instructions. Human embryos were cultured in pre-equilibrated Global Media supplemented with 5 mg/ml Life Global HSA (both LifeGlobal, LGG-020 and LGPS-605) and overlaid with mineral oil (Origio, ART-4008-5P) and incubated in an Embryoscope+ time-lapse incubator (Vitrolife). Embryos were grouped into day 5, 6 or 7 based on data provided from the clinic. For the collection of day 5 samples, embryos were fixed approximately 2 h after thawing to allow them to recover. To collect day 6 and 7 samples, day 5 or 6 embryos were cultured for the appropriate time before fixation.

### Microdissection of TE from human blastocysts

Embryos were placed in drops of G-MOPS solution (Vitrolife, 10129) on a Petri dish overlaid with mineral oil. The plate was placed on a microscope stage (Olympus IX70) and the embryos were held with an opposing holding pipette and blastomere biopsy pipette (Research Instruments) using micromanipulators (Narishige). The biopsy mode of a Saturn 5 laser (Research Instruments) was used to separate the mural TE from the ICM and polar TE. The separated mural TE was transferred to individual low-bind RNase-free tube containing 0.25 μl RNase inhibitor, 4.75 μl dilution buffer (SMARTer Ultra Low Input RNA kit; Clontech, 634820) and 5 μl nuclease-free water on a pre-chilled CoolRack (Biocision). Samples were stored at −80°C until processing.

### cDNA synthesis and library preparation of TE samples

cDNA was synthesised using the SMARTer Ultra Low Input RNA for Illumina Sequencing-HV kit (Clontech Laboratories, 634820) according to the manufacturer's instructions and as previously published (Blakeley et al., 2015; Hyslop et al., 2016). cDNA was sheared using a Covaris S2 instrument with the modified settings 10% duty, intensity 5, burst cycle 200 for 2 min. Libraries were prepared using a Low Input Library Prep Kit (Clontech Laboratories, 634900) according to the manufacturer's instructions. Library quality was assessed with an Agilent 2100 BioAnalyzer and concentration measured by QuBit broad range assay (Thermo Fisher Scientific, Q32850). Prepared libraries were submitted for 50-bp paired-end sequencing on an Illumina HiSeq 2000 sequencing system.

### cDNA synthesis and library preparation of bulk cell lines

For bulk RNA-seq of cell lines, RNA was isolated using TRI reagent (Sigma-Aldrich, T9424) and treated with DNase I (Ambion, AM2222). Libraries were prepared using the polyA KAPA mRNA HyperPrep Kit (Roche, 8098115702). The quality of submitted RNA samples and the resulting cDNA libraries was determined by ScreenTape Assay on a 4200 TapeStation (Agilent). Prepared libraries were submitted for single-end 75 bp sequencing on an Illumina HiSeq 4000 (Illumina).

### RNA-seq analysis of TE samples

RNA-seq data from human TE was analysed as previously described (Blakeley et al., 2015; Hyslop et al., 2016). Briefly, the reference human genome sequence was obtained from Ensembl, along with the gene annotation file (GTF). The reference sequence was indexed using the ‘bowtie2-build’ command. Reads were aligned to the reference human genome sequence using TopHat2 (Kim et al., 2019), with gene annotations to obtain BAM files for each sample. BAM files were then sorted by read coordinates and converted into SAM files using SAMtools. The process of mapping and processing BAM files was automated using a custom Perl script. The number of reads mapping to each gene were counted using the program HTSeq-count (Version 0.6.1; Anders et al., 2015). The resulting count files for each sample were used as input for differential expression analysis using DESeq2 (Anders and Huber, 2010). First, the functions ‘estimateSizeFactors’ and ‘estimateDispersions’ were used to estimate biological variability and calculate normalised relative expression values across the different blastocyst samples. Initially, this was performed without sample labels (option: method=‘blind’) to allow unsupervised clustering of the blastocyst samples using PCA and hierarchical clustering. The dispersion estimates were recalculated with the sample labels included and with the option: method=‘pooled’. The function ‘nbinomTest’ was then used to calculate *P*-values to identify genes showing significant differences in expression between different developmental stages.

An RPKM >5 threshold was applied to generate the lists of genes expressed at each stage analysed. Overlap between the gene lists was determined using the online web application GeneVenn (Pirooznia et al., 2007). Gene lists were used to perform a Gene Ontology and Reactome functional enrichment analysis (Ashburner et al., 2000; Fabregat et al., 2018) to identify over-represented categories using a significance threshold of *P*≤0.05.

### RNA-seq data analysis of induced trophoblast stem cells (5F-iTSCs) and related lines

#### Data from this study

Sequencing data from sample replicates comprising 5F-iTSCs (*n*=4), H1 ESCs (*n*=3) and primary CT27 cells (*n*=3) were first checked using the FastQC package (https://www.bioinformatics.babraham.ac.uk/projects/fastqc/). Adapter removal was performed using Trimgalore v0.6.6 (https://github.com/FelixKrueger/TrimGalore), and the trimmed read data were re-checked for conformity and quality with FastQC.

### Accessory datasets

Primary CTB gene expression data were downloaded from Gene Expression Omnibus (GEO) with accession number GSE109976 (Haider et al., 2018). Additional primed hESCs gene expression data were downloaded from GEO with accession numbers GSM4116153 and GSM4116151 (Dong et al., 2020). TSCs derived from human blastocysts gene expression data were downloaded from GSE138762 (Dong et al., 2020). TSCs derived from fibroblast reprogramming gene expression data were downloaded from GSE150616 (Liu et al., 2020) and GSE138762 (Dong et al., 2020).

Next, sequences were aligned to the reference genome (Homo_sapiens.GRCh38) using HISAT2 v2.2.1 (Kim et al., 2019), with trimming of 5′ and 3′ bases performed based on the QC of each of each of these samples. Counts were generated using FeatureCounts v2.0.1, and the count matrix was analysed further using DESeq2 (Love et al., 2014; Liao et al., 2019). Genes with fewer than ten counts detected across all 22 samples were excluded, leaving 17,498 genes for further processing. A variance stabilizing transformation (VST) was applied and batch-related effects in combining these different datasets were corrected for using the limma package (i.e. *limma::*removeBatchEffect) (Law et al., 2016). Sample-to-sample distances were computed and plotted as a heatmap. PCA was performed using the top 500 most highly variable genes.

### Generation of inducible system

Doxycycline-inducible overexpression of transcription factors was achieved using the Lenti-X Tet-On 3G Inducible Expression System (Clontech, 631363) following the manufacturer's protocol and as previously described (Wamaitha et al., 2015). Coding sequences were sub-cloned from the template plasmids into the pLVX-TRE3G vector to generate individual pLVX-TRE3G-Gene of Interest (GOI) vectors (plasmids available upon request). Lentiviral packaging was achieved using 7 μg of pLVX-TRE3G-GOI and the Lenti-X Packaging Single Shot reagents in HEK293T cells. Transfection media was replaced after 6 h with fresh MEF media. Lentiviral supernatants were subsequently collected after transfection of HEK293T cells with either the pLVX-TRE3G-GOI or the pLVX-Tet3G vector and concentrated using X-fect reagent (Clontech, 631317). Supernatants were concentrated by ultracentrifugation (25,000 ***g*** for 90 min at 4°C). Equal volumes of lentivirus supernatant encoding *GATA2*, *GATA3*, *TFAP2C*, *MYC* and *KLF5* were pooled to generate a 5F cocktail, which was aliquoted into single-use 10 µl volumes. hESCs were grown to 70% confluency and transduced with the pLVX-tet3G lentivirus followed by selection with G418 (250 μg/ml) for 1 week. G418-resistant cells were selected for at least two passages and then transduced with the pLVX-TRE3G-GOI lentivirus pool, followed by selection with puromycin (0.5 μg/ml) for 4 days. For the induction of GOI expression, doxycycline was added to mTeSR1 media (STEMCELL Technologies, 85850) at a concentration of 1 μg/ml. Clonal lines were generated and screened for transgene integration. Transgene integration was confirmed by culturing clonal lines in the presence of doxycycline in mTeSR for 48 h and transgene expression was detected by RT-qPCR using primers distinguishing exogenous expression from endogenous expression; TFAP2C expression was assessed by immunofluorescence analysis owing to difficulties in designing primers to distinguish between exogenous and endogenous transcripts. Primer sequences used to detect transgene expression are detailed in Table S5.

### Generation of templates for lentivirus production and *in vitro* transcription

The vector design strategy was informed from previously published reports (Mandal and Rossi, 2013). Briefly, nucleotide sequences of the open reading frames for the canonical isoforms of transdifferentiation factors were identified from the Ensembl genome browser, and the sequences were verified against the corresponding amino acid sequences in UniProt. Individual *in vitro* transcription template constructs for *GATA2*, *GATA3*, *TFAP2C* and *KLF5* consisting of a T7 promoter-5′UTR-Open Reading Frame-3′UTR-T7 terminator cassette cloned into a pUC57 backbone were custom made (GENEWIZ UK Ltd.). 5′UTR and 3′UTRs were added to maximize stability of mRNA transcripts and to increase protein translation. The open reading frames of *MYC* and *GFP* were templated from plasmids bearing human *MYC* and *GFP* and ligated into the pUC57 backbone flanked by the 5′UTR and 3′UTR sequences. Annotated sequence files of all constructs are provided in Table S7.

### Generation of modified mRNAs by *in vitro* synthesis

The mRNA synthesis protocol has been described previously (Mandal and Rossi, 2013). Briefly, dsDNA templates were linearised from cDNA clones in pLVX vectors for *GATA2*, *GATA3*, *TFAP2C*, *KLF5* and *MYC*. A small amount of digestion mix was run on a gel to confirm complete digestion. Linearised plasmid was purified using a PCR purification kit (QIAGEN, 28104). A NanoDrop spectrophotometer was used to confirm the purity of the eluted product according to the 260/280 ratios. Poly(A) tail was added using KAPA PCR ready mix (2×), Xu-F1 and Xu-T120 primers (Integrated DNA Technologies) and digested plasmid adjusted to 10 ng/μl. Tail PCR was run for 32 cycles and purified using the PCR purification kit. *In vitro* transcription was performed using MEGAscript T7 kit (Thermo Fisher Scientific, AMB13345): custom NTP mix was prepared with 3′-O-Me-m7G cap analogue (60 mM, NEB), GTP (75 mM, MEGAscript T7 kit), ATP (75 mM, MEGAscript T7 kit), Me-CTP (100 mM; TriLink Biotechnologies, N-1014-1) and pseudo-UTP (100 mM; TriLink Biotechnologies, O-0263). The reaction mixture was heated at 37°C for 2 h then 2 μl of Turbo DNase (Thermo Fisher Scientific, AM2238) was added and incubated at 37°C for 15 min. The DNase-treated reaction mix was purified using an RNeasy kit (QIAGEN, 74104) according to the manufacturer's instructions. RNA was phosphatase-treated using Antarctic phosphatase (New England BioLabs, M0289S) and purified using a MEGAclear kit (Thermo Fisher Scientific, AM1909). Concentration and quality were measured using a NanoDrop spectrophotometer and adjusted to 100 ng/μl. For transdifferentiation experiments, a modified mRNA cocktail was prepared by mixing the five factors in an equal molar ratio. The cocktail was aliquoted into single-use aliquots containing a total mRNA amount of 1 µg and stored at −80°C until needed. For transcription factor combinatorial experiments, cocktails were made up to a total amount of 500 ng mRNA with the relevant quantity of mRNA encoding GFP replacing the omitted factors.

### Cell culture

H1 hESCs (WiCell) cells were routinely cultured in mTeSR1 (STEMCELL Technologies, 85850) and growth factor-reduced Matrigel (BD Biosciences, 356321). For hTSC transdifferentiation experiments, H1 cells were plated in mTeSR1 media on Matrigel-coated cell culture dishes. Media was changed for hTSC media the next day. hTSC media was prepared as described previously (Okae et al., 2018) (CHIR99021, BioTechne, 4423/10; EGF, Tebu Bio Ltd., 167AF-100-15-a; ITS-X supplement, Fisher Scientific, 10524233; L-ascorbic acid, Tocris, 4055/50; A83-01, Sigma-Aldrich, SML0788/5MG; SB-431542, Cambridge Bioscience, SM33-2; valproic acid, Sigma-Aldrich, V-006-1ML; Y27632, STEMCELL Technologies, 72302). For transgene induction, doxycycline was added daily at a concentration of 1 μg/ml over 20 days for transgene induction. hTSCs were passaged weekly by dissociation with TrypLE Express (Thermo Fisher Scientific, 12604013) for 15 min at 37°C and passaged en masse initially onto collagen IV-coated (5 µg/ml; Corning, 354233) 10 cm plates and subsequently onto 6-well plates and maintained in hTSC media. CT27 and BTS5 hTSCs (Riken BRC) were routinely cultured in hTSC media as described above on collagen IV-coated plates. Cells were routinely tested for *Mycoplasma* contamination using PCR-based detection assays.

### Lipofection of hESCs

For one well of a 6-well plate, 1 µg (1×) of mRNA was mixed with 86 µl of OptiMEM reduced serum media in an Eppendorf tube. In a second tube, 0.5× volume of Lipofectamine RNAiMAX (Thermo Fisher Scientific, 13778100) was mixed with 93 µl OptiMEM. Tubes were incubated at room temperature for 5 min. The tubes were mixed together and incubated at room temperature for 20 min. The lipofection mix was added to the well in a dropwise manner and mixed well by rocking the plate from side to side. The cells were incubated at 37°C in 20% O_2_ for 4 h. The media was then replaced with hTSC media containing 200 ng/ml recombinant B18R to prevent gamma-interferon response (STEMCELL Technologies, 78075). For transdifferentiation experiments, lipofection was performed at the same time every day for 20 days. Mock transfection was performed using an equivalent amount of *GFP* mRNA for 20 days. For transcription factor combinatorial experiments, hESCs were lipofected daily for 10 days with an mRNA transfection cocktail totalling 500 ng, with individual factors being replaced with an equivalent amount of *GFP* mRNA.

### Directed differentiation

For the induction of STB, 0.4×10^5^ 5F-iTSCs or hTSCs were seeded in hTSC media in a 12-well plate pre-coated with 2.5 mg/ml collagen IV. Cells were allowed to adhere for a minimum of 6 h before media was changed to STB media comprising DMEM/F12 supplemented with 0.1 mM 2-mercaptoethanol, 0.5% penicillin-streptomycin, 0.3% bovine serum albumin, 1% ITS-X supplement, 2.5 mM Y27632, 2 mM forskolin and 4% KSR. The media was replaced every 2 days, and resultant cells were analysed at day 6.

For induction of EVTs, 0.8×10^5^ 5F-iTSCs and hTSCs were seeded in hTSC media in 12-well plates pre-coated with 1 mg/ml collagen IV. Cells were allowed to adhere for a minimum of 6 h before media was changed to EVT1 media comprising DMEM/F12 with 0.1 mM 2-mercaptoethanol, 0.5% penicillin-streptomycin, 0.3% bovine serum albumin, 1% ITS-X supplement, 100 ng/ml NRG1, 7.5 μM A83-01, 2.5 μM Y27632 and 4% KSR. Cold growth factor reduced Matrigel was added to yield a final concentration of 2%. After 72 h, media was replaced with EVT2 media, which lacks NRG1, and Matrigel was added to yield a final concentration of 0.5%.

### Immunostaining

Embryos were fixed with 4% paraformaldehyde in PBS for 1 h at 4°C. Immunofluorescence staining was performed as described previously (Fogarty et al., 2017). The primary antibodies used are listed in Table S6. Embryos were placed on µ-Slide 8-well coverslip dishes for confocal imaging (Ibidi, 80826). Imaging was performed using a Leica SP5 confocal microscope and 3-μm-thick optical sections were collected. hESC, iTSC and hTSC lines were fixed with PBS 4% paraformaldehyde for 1 h at 4°C, then washed three times with PBS (Life Technologies, 14190-094). Blocking was achieved by incubation in PBS with 10% donkey serum (Sigma-Aldrich, D9663) and 0.1% Triton X-100 (Sigma-Aldrich, T8787) for 30 mins at room temperature. Permeabilization was performed by incubation in PBS with 0.5% Triton X-100. Primary antibodies were diluted in blocking solution and each incubated overnight at 4°C. Following each incubation, the cells underwent three 5 min washes with PBS with 0.1% Triton X-100. Secondary antibodies were diluted 1:300 in PBS with 0.1% Triton X-100 for 1 h at room temperature. After three 5 min washes in PBS with 0.1% Triton X-100, 5 μg/ml DAPI (Sigma-Aldrich, D9542) was added for 2 min during the final wash to stain nuclei. Confocal imaging analysis of embryos was performed using a Leica SP5 confocal microscope. Fluorescent imaging of cells was performed using an Olympus IX73 inverted microscope using CellSens software (Olympus) and a Zeiss Meta LSM510 confocal microscope with Zen Pro 2102 software. For quantification of immunostaining analysis of 2D cells, an average of 250 (Fig. 8) or 150 (Fig. S3) cells from five random frames of view were counted from two independent experiments.

### RT-qPCR

RNA was isolated using TRIzol Reagent (Sigma-Aldrich) and DNaseI treatment (Ambion). cDNA was synthesised using a Maxima First Strand cDNA Synthesis Kit (Fermentas). RT-qPCR was performed using Sensimix SYBR Low-Rox kit (Bioline, QT625) on a Bio-Rad CFX96 Touch Real-Time PCR Detection System. Primer pairs (listed in Table S5) were either previously published, as referenced, or designed using Primer3 software. RT-qPCR for the detection of miRNAs was performed using primers and protocols as previously published (Lee et al., 2016). cDNA was synthesised using the TaqMan™ MicroRNA Reverse Transcription Kit (Applied Biosystems, 4366597). RT-qPCR was performed on a CFX384 Touch Real-Time PCR Detection System (Bio-Rad, 1855484) and CFX Maestro software. Statistical analysis was performed using Prism 10 (GraphPad).

### *ELF5* promoter methylation analysis

Bisulfite sequencing of the *ELF5* promoter was carried out as described previously (Bernardo et al., 2011). Briefly, bisulphite conversion was carried out on 400 ng genomic DNA using the EpiTect Bisulfite kit (QIAGEN) following the manufacturer's instructions. Nested PCR was carried out on 10% of the eluted DNA to analyse −432/−3 bp relative to the *ELF5* transcriptional start site. Purified PCR products were cloned using the pGEM-T Easy Vector System (Promega) and sequenced.

### Fusion index calculation

STBs differentiated from control hTSCs and 5F-iTSCs were subjected to immunofluorescence analysis using phalloidin to stain filamentous actin and DAPI to stain the nuclei. The number of DAPI-stained nuclei and syncytia were counted in 20 random fields of view imaged at 10×. Syncytia were defined as regions containing at least three nuclei. The fusion index was calculated as following: (number nuclei in the syncytia – number of syncytia)/total number of nuclei×100.

## Supplementary Material



10.1242/develop.202778_sup1 Supplementary information

Table S1. Bulk RNA-sequencing analysis of TE across human embryo development. RNA-seq data is normalised using the RPKM method.

Table S2. Genes expressed in common or unique to a specific stage of TE development. Genes were considered to be expressed if RPKM>5.

Table S3. Transcription factors detected in human TE by RNA-seq analysis.
