# Supplementary material for: Transcription factor-based transdifferentiation of human embryonic to trophoblast stem cells
Source: Development. 2024 Sep 10;151(17):dev202778. doi: 10.1242/dev.202778 (PMC11556314; doi:10.1242/dev.202778)
Supplement: Supplementary information [file develop-151-202778-s1.pdf]

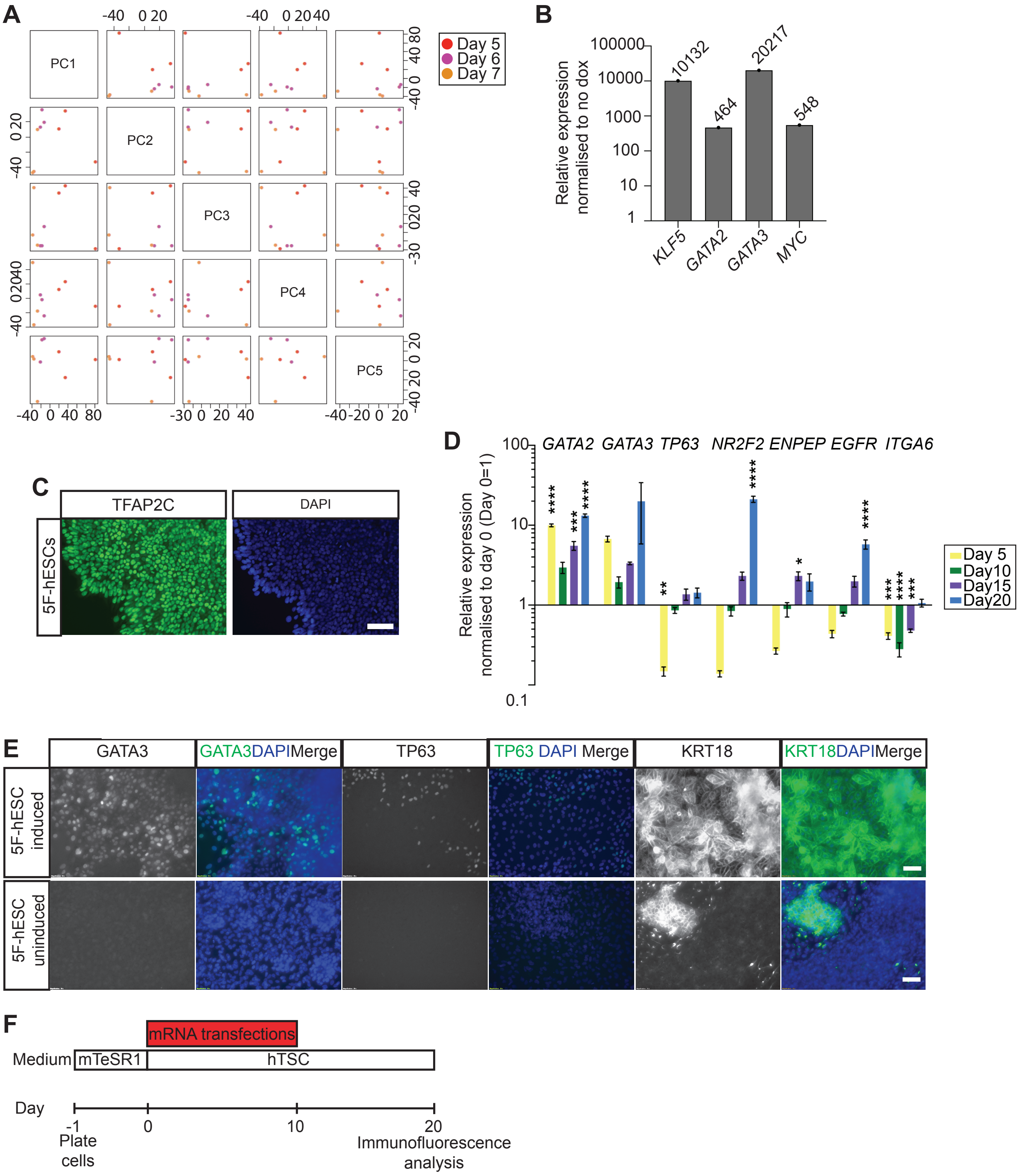

Fig. S1. Principal component analysis matrix using the first five principal components for the top 12,000 most variable expressed genes in day 5, 6 and 7 TE samples. (B) RT-qPCR analysis for the detection of exogenous *KLF5*, *GATA2*, *GATA3* and *MYC* in 5F-hESCs after 48 h of doxycycline treatment. Relative expression is reflected as fold change over uninduced 5F-hESCs cultured normalized to *GAPDH.* (C) Immunofluorescence analysis for the detection of TFAP2C in 5F-hESCs after 48 h of doxycycline. (D) Time-course qRT-PCR analysis for the detection of endogenous *GATA2*, *GATA3*, *TP63*, *NR2F2*, *ENPEP*, *EGFR* and *ITGA6* in 5F-hESCs across 20 days of dox-induction in hTSC media. Relative expression is reflected as fold change over 5F-hESCs at day 0 plated in mTSeR pluripotency media, normalized to *GAPDH*. (E) Immunofluorescence analysis for the detection of GATA3, TP63 and KRT18 (green) and DAPI nuclear expression (blue) in doxycycline-induced and uninduced 5F-hESCs on day 20. Scale bars: 50 μm. (F) Schematic representation of the strategy for combinatorial transdifferentiation experiments using modified mRNAs.

Development • Supplementary information

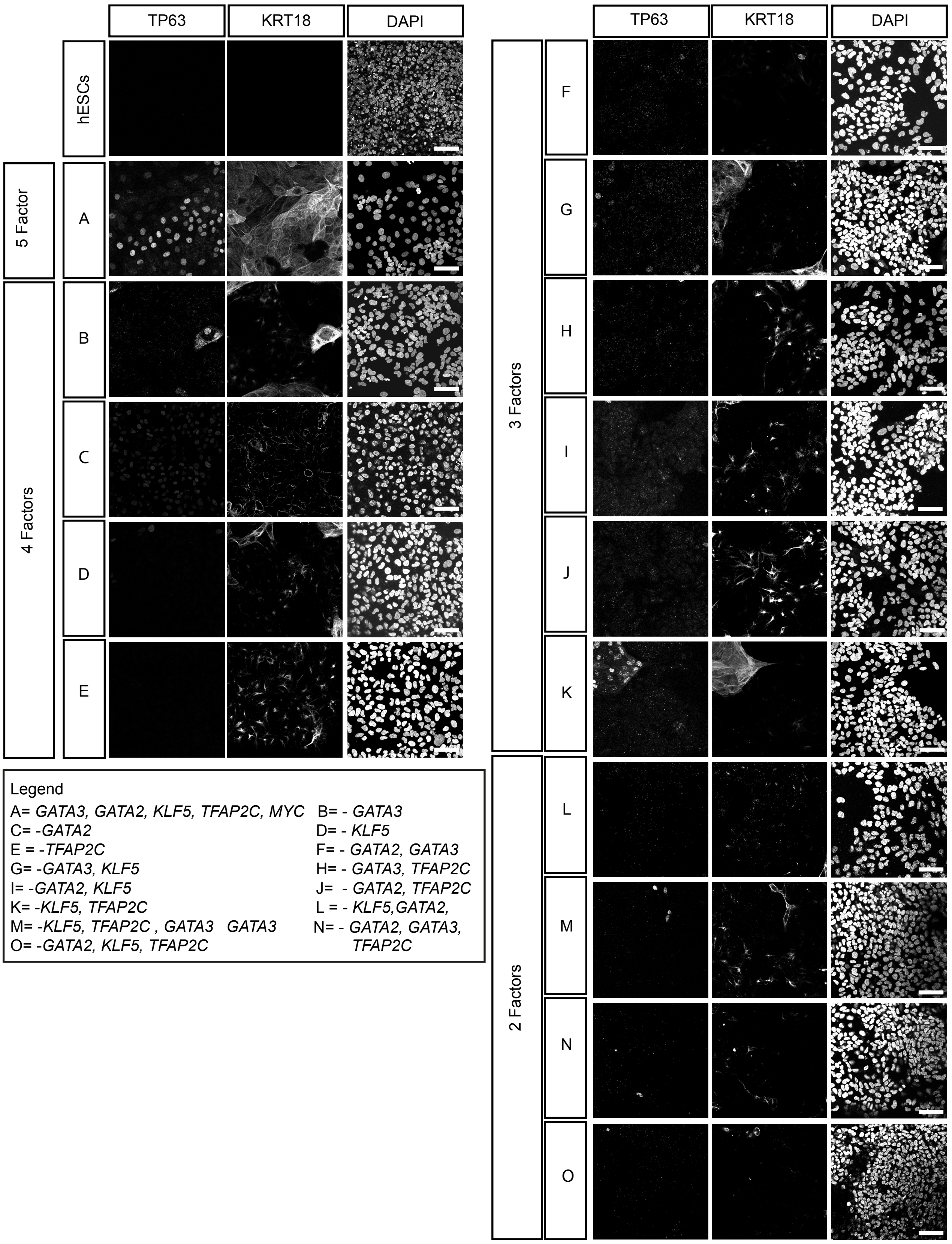

Fig. S2. Extended immunofluorescence analysis for the detection of TP63, KRT18 and DAPI nuclear expression in transfected cells. Key for mRNA cocktail combinations: A, Five factors positive controls; B, *GATA2*, *TFAP2C*, *KLF5* and *MYC* (*GATA3* omitted); C, *GATA3*, *TFAP2C*, *KLF5* and *MYC* (*GATA2* omitted); D, *GATA2*, *GATA3*, *TFAP2C* and *MYC* (*KLF5* omitted); E, *GATA2*, *GATA3*, *KLF5* and *MYC* (*TFAP2C* omitted); F, *TFAP2C*, *KLF5* and *MYC* (*GATA2* and *GATA3* omitted); G, *GATA2*, *TFAP2C* and *MYC* (*GATA3* and *KLF5* omitted); H, *GATA2*, *KLF5* and *MYC* (*GATA3* and *TFAP2C* omitted); I, *GATA3*, *TFAP2C* and *MYC* (*GATA2* and *KLF5* omitted); J, *GATA3*, *KLF5* and *MYC* (*GATA2* and *TFAP2C* omitted); K, *GATA2*, *GATA3* and *MYC* (*TFAP2C* and *KLF5* omitted); L, *TFAP2C*, *KLF5* and *MYC* (*KLF5*, *GATA2* and *GATA3* omitted); M, *GATA2* and *MYC* (*GATA3*, *TFAP2C* and *KLF5*); N, *KLF5* and *MYC* (*GATA2*, *GATA3* and *TFAP2C* omitted). hESCs cultured in mTeSR1 media are included as a negative control. Scale bars: 50 µm.

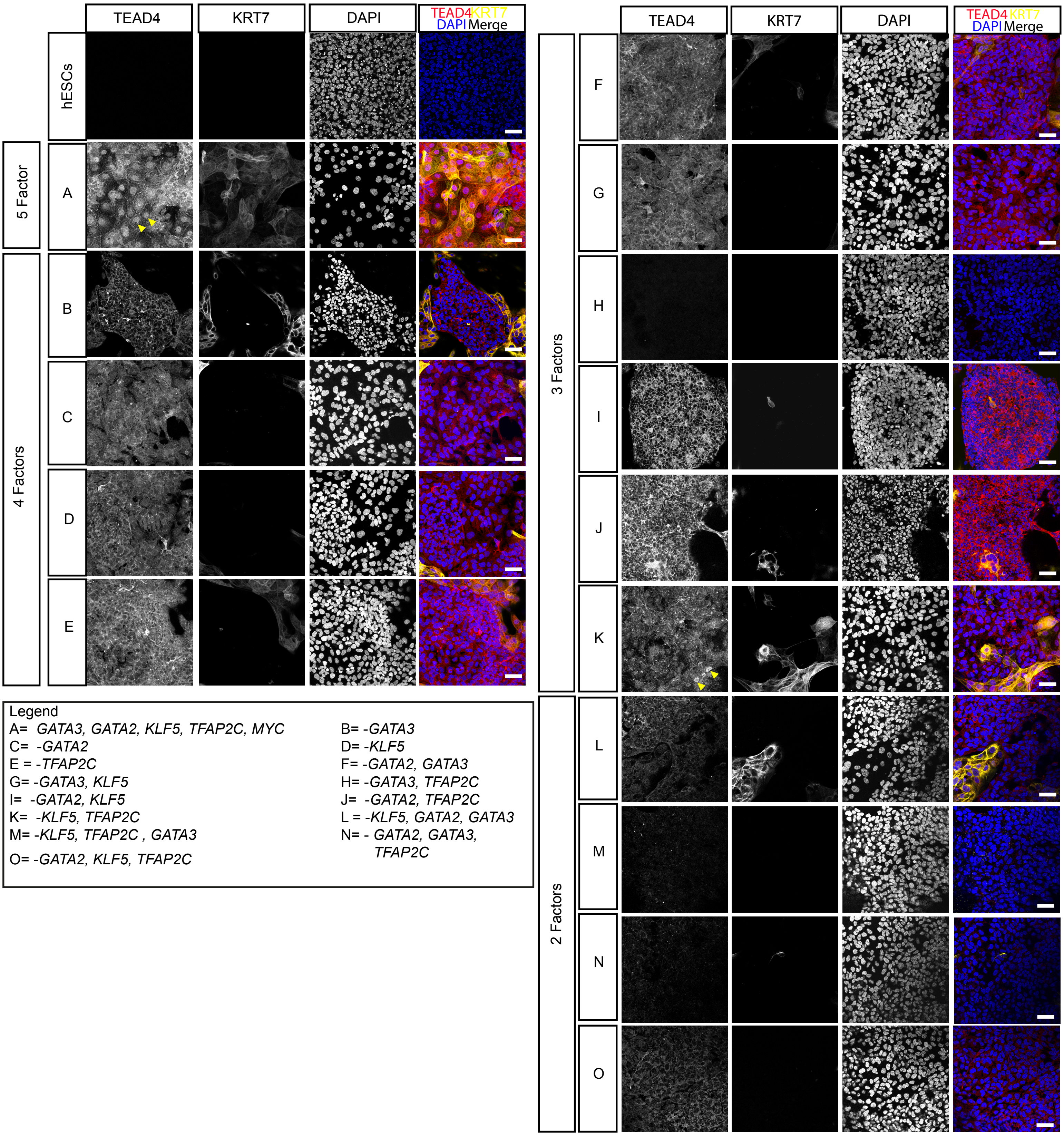

Development • Supplementary information

Fig. S3. Immunofluorescence analysis for the detection of TEAD4 (red), KRT7 (yellow) and DAPI (blue) nuclear expression in transfected cells. Key for mRNA cocktail combinations: A, Five factors positive controls; B, *GATA2*, *TFAP2C*, *KLF5* and *MYC* (*GATA3* omitted); C, *GATA3*, *TFAP2C*, *KLF5* and *MYC* (*GATA2* omitted); D, *GATA2*, *GATA3*, *TFAP2C* and *MYC* (*KLF5* omitted); E, *GATA2*, *GATA3*, *KLF5* and *MYC* (*TFAP2C* omitted); F, *TFAP2C*, *KLF5* and *MYC* (*GATA2* and *GATA3* omitted); G, *GATA2*, *TFAP2C* and *MYC* (*GATA3* and *KLF5* omitted); H, *GATA2*, *KLF5* and *MYC* (*GATA3* and *TFAP2C* omitted); I, *GATA3*, *TFAP2C* and *MYC* (*GATA2* and *KLF5* omitted); J, *GATA3*, *KLF5* and *MYC* (*GATA2* and *TFAP2C* omitted); K, *GATA2*, *GATA3* and *MYC* (*TFAP2C* and *KLF5* omitted); L, *TFAP2C*, *KLF5* and *MYC* (*KLF5*, *GATA2* and *GATA3* omitted); M, *GATA2* and *MYC* (*GA*TA3, *TFAP2C* and *KLF5*); N, *KLF5* and *MYC* (*GATA2*, *GATA3* and *TFAP2C* omitted). hESCs cultured in in mTeSR1 media are included as a negative control.

Table S1. Bulk RNA-sequencing analysis of TE across human embryo development. RNA-seq data is normalised using the RPKM method.

Available for download at
https://journals.biologists.com/dev/article-lookup/doi/10.1242/dev.202778#supplementary-data

Table S2. Genes expressed in common or unique to a specific stage of TE development. Genes were considered to be expressed if RPKM>5.

Available for download at
https://journals.biologists.com/dev/article-lookup/doi/10.1242/dev.202778#supplementary-data

Table S3. Transcription factors detected in human TE by RNA-seq analysis.

Available for download at
https://journals.biologists.com/dev/article-lookup/doi/10.1242/dev.202778#supplementary-data

Development • Supplementary information

Table S4. DESeq2 analysis was used to calculate the probability of differential expression between human TE and EPI. RPKM values and log2-fold change difference in expression are noted.

DESeq2 analysis

	TE mean	EPI mean	Log2FC	p-value
GATA2	4528.6	16.1	8.1	2.20E-19
GATA3	8457.3	255.1	5	4.06E-13
KLF5	4609.4	1931.3	2.4	0.08
TFAP2C	1525.7	1135.1	0.4	0.9
MYC	3830.4	1405.5	1.4	0.6

RPKM values at time-points analysed

	Day 5	Day 6	Day 7
GATA2	19.3	65.6	52.5
GATA3	93.8	120.4	96.8
KLF5	45.2	45.2	46.7
TFAP2C	644.2	98.8	393.7
MYC	32.9	50.9	39.5

Development • Supplementary information

Table S5. Oligonucleotide sequences used for qRT-PCR analysis.

Oligo ID	Sequence	Reference	Oligo ID	Sequence
GAPDH _F	GATGACATCAAGAAGGTGGTG		*GATA3_3UTR* _F	GGTGTCTGTGTTCCAACCAC
GAPDH _R	GTCTACATGGCAACTGTGAGG		*GATA3_3UTR* _R	GTGGCCAGTGAAAGGAAACA
TP63 _F	CTGGAAAACAATGCCCAGA	Soncin et al. 2018	*GATA3* _F	CCGCCCTACTACGGAAACTC
TP63 _R	AGAGAGCATCGAAGGTGGAG	Soncin et al. 2018	*GATA3* _R	TCTTGGAGAAGGGGCTGAGA
EGFR _F	CTAAGATCCCGTCCATCGCC	Soncin et al. 2018	*GATA2_3UTR* _F	AGGCCACTGACCATGAAGAA
EGFR _R	GGAGCCCAGCACTTTGATCT	Soncin et al. 2018	*GATA2_3UTR* _R	CGACGTCCATCTGTTCCCTA
ITGA6 _F	GGCGGTGTTATGTCCTGAGTC		*GATA2* _F	GACTACAGCAGCGGACTCTT
ITGA6 _R	AATCGCCCATCACAAAAGCTC		*GATA2* _R	GTTGTCGTCTGACAATTTGC
ENPEP _F	AATTTATGTCCAGCCAGAGC			
ENPEP _R	GTGATGAGTCCCCAGTTCTC			
NR2F2 _F	GCCATAGTCCTGTTCACCTCA	Io et al. 2021		
NR2F2 _R	AATCTCGTCGGCTGGTTG	Io et al. 2021		
ENDOU _F	ACAGGGCAGACACCAACAAA			
ENDOU _R	AGGAGGTTGATGAAGGCTGC			
GCM1 _F	TGCTGTCTGCTTCTCCGTAA			
GCM1 _R	CACCTATTCGACTCCCCTCA			
PSG3 _F	TCGTAAAGCGAGGTGATGGG	Io et al. 2021		
PSG3 _R	AAGCTCACAGCCTCCATGTC	Io et al. 2021		
SDC1 _F	CTTCACACTCCCCACACAGA	Io et al. 2021		
SDC1 _R	GTATTCTCCCCCGAGGTTTC	Io et al. 2021		
HLAG _F	CCACCACCCTGTCTTTGACTAT	Io et al. 2021		
HLAG _R	ACGTCCTGGGTCTGGTCCT	Io et al. 2021		
LRRC32 _F	GCTGCACAACACCAAGACAAA	Io et al. 2021		
LRRC32 _R	GATCAAGGGTCTCAGTGTCTGG	Io et al. 2021		
LVRN _F	GGGAGGGACTCTTCCTCAAC	Io et al. 2021		
LVRN _R	GGGAAAACATACCTGGCAAA	Io et al. 2021		
MMP2 _F	CCCTGTGTCTTCCCCTTCAC			
MMP2 _R	ATCGTAGTTGGCTGTGGTCG			
NOTUM _F	TTTGGCTACAAGGTCTACCCG	Io et al. 2021		
NOTUM _R	TCAAACAGCCACTGCACCAC	Io et al. 2021		
GABRP _F	GCCCTAACAGAGCCTCAACA	Rostovkaya et al. 2022		
GABRP _R	CCCTGGATGCACATCCTCTC	Rostovkaya et al. 2022		
HEY1 _F	GCTGGTACCCAGTGCTTTTGAG	Io et al. 2021		
HEY1 _R	CAAGGGCGTGCGCGTCAAAGTA	Io et al. 2021		
ISL1 _F	TCTCCGGATTTGGAATGGCA	Yang et al. 2021		
ISL1 _R	CCTTGCACCGCTTGTTTTGA	Yang et al. 2021		
WNT6 _F	CAGCCCCTTGGTTATGGACC			
WNT6 _R	TCTCCCGAATGTCCTGTTGC			
MYC_3UTR _F	ACCCTTCGCTATCATGCCTT			
MYC_3UTR _R	TCTTGGGCATGTGGATGAGT			
MYC _F	CGTCCTCGGATTCTCTGCTC			
MYC _R	GCTGCGTAGTTGTGCTGATG			
KLF5_3UTR_F	GGGCTCCCTCAAATGACAGA			
KLF5_3UTR _R	CCACCCCTTACCCATGTTGA			
KLF5 _F	CCACCACCCTGCCAGTTAAC	Takeda et al. 2022		
KLF5 _R	TAAACTTTTGTGCAACCAGGGTAA	Takeda et al. 2022		

Table S6. Antibodies used for immunofluorescence analysis.

Antibody	Species	Supplier	Catalogue Number
OCT4	Mouse IgG2b	Santa Cruz	SC-5279
NANOG	Goat	R&D	AF1997
GATA3	Goat	R&D	AF2605
TFAP2C	Goat	R&D	AF5059
GATA2	Rabbit	Santa Cruz	SC-9008
NANOG	Rabbit	Abcam	ab21624
KLF5	Rabbit	Abcam	ab137676
CYTOKERATIN18	Mouse IgG1	Abcam	ab668
TP63	Mouse	Biocare	3066
GCM1	Rabbit	Sigma	HPA011343
HAND1	Rabbit	Abcam	ab196622
TBX3	Goat	Santa Cruz	SC-17871
SOX2	Rat	eBioscience	14-9811-82
TEAD4	Mouse IgG2a	Abcam	ab58310
CYTOKERATIN7	Mouse	Agilent Dako	GA61961-2
Alexa Fluor 488 Phalloidin		ThermoFischer	A12381

Table S7. Annotated sequence files of constructs used to generate modified mRNAs. T7 promoters and T7 terminators are in bold font and underlined. 5’UTR and 3’UTR sequences are in bold font.

GATA2 isoform 1
TAATACGACTCACTATAGGG**AAATAAGAGAGAAAAGAAGAGTAAGAAGAAATATAAGAGCCACC**ATGGAGGTGGC
GCCCGAGCAGCCGCGCTGGATGGCGCACCCGGCCGTGCTGAATGCGCAGCACCCCGACTCACACCACCCGGGCCT
GGCGCACAACTACATGGAACCCGCGCAGCTGCTGCCTCCAGACGAGGTGGACGTCTTCTTCAATCACCTCGACTC
GCAGGGCAACCCCTACTATGCCAACCCCGCTCACGCGCGGGCGCGCGTCTCCTACAGCCCCGCGCACGCCCGCCT
GACCGGAGGCCAGATGTGCCGCCCACACTTGTTGCACAGCCCGGGTTTGCCCTGGCTGGACGGGGGCAAAGCAGC
CCTCTCTGCCGCTGCGGCCCACCACCACAACCCCTGGACCGTGAGCCCCTTCTCCAAGACGCCACTGCACCCCTC
AGCTGCTGGAGGCCCTGGAGGCCCACTCTCTGTGTACCCAGGGGCTGGGGGTGGGAGCGGGGGAGGCAGCGGGAG
CTCAGTGGCCTCCCTCACCCCTACAGCAGCCCACTCTGGCTCCCACCTTTTCGGCTTCCCACCCACGCCACCCAA
AGAAGTGTCTCCTGACCCTAGCACCACGGGGGCTGCGTCTCCAGCCTCATCTTCCGCGGGGGGTAGTGCAGCCCG
AGGAGAGGACAAGGACGGCGTCAAGTACCAGGTGTCACTGACGGAGAGCATGAAGATGGAAAGTGGCAGTCCCCT
GCGCCCAGGCCTAGCTACTATGGGCACCCAGCCTGCTACACACCACCCCATCCCCACCTACCCCTCCTATGTGCC
GGCGGCTGCCCACGACTACAGCAGCGGACTCTTCCACCCCGGAGGCTTCCTGGGGGGACCGGCCTCCAGCTTCAC
CCCTAAGCAGCGCAGCAAGGCTCGTTCCTGTTCAGAAGGCCGGGAGTGTGTCAACTGTGGGGCCACAGCCACCCC
TCTCTGGCGGCGGGACGGCACCGGCCACTACCTGTGCAATGCCTGTGGCCTCTACCACAAGATGAATGGGCAGAA
CCGACCACTCATCAAGCCCAAGCGAAGACTGTCGGCCGCCAGAAGAGCCGGCACCTGTTGTGCAAATTGTCAGAC
GACAACCACCACCTTATGGCGCCGAAACGCCAACGGGGACCCTGTCTGCAACGCCTGTGGCCTCTACTACAAGCT
GCACAATGTTAACAGGCCACTGACCATGAAGAAGGAAGGGATCCAGACTCGGAACCGGAAGATGTCCAACAAGTC
CAAGAAGAGCAAGAAAGGGGCGGAGTGCTTCGAGGAGCTGTCAAAGTGCATGCAGGAGAAGTCATCCCCCTTCAG
TGCAGCTGCCCTGGCTGGACACATGGCACCTGTGGGCCACCTCCCGCCCTTCAGCCACTCCGGACACATCCTGCC
CACTCCGACGCCCATCCACCCCTCCTCCAGCCTCTCCTTCGGCCACCCCCACCCGTCCAGCATGGTGACCGCCAT
GGGCTAG**GCTGCCTTCTGCGGGGCTTGCCTTCTGGCCATGCCCTTCTTCTCTCCCTTGCACCTGTACCTCTTGGT**
CTTTGAATAAAGCCTGAGTAGGAAGTAGCATAACCCCTTGGGGCCTCTAAACGGGTCTTGAGGGGTTTTTTG

GATA3 isoform 1
TAATACGACTCACTATAGGG**AAATAAGAGAGAAAAGAAGAGTAAGAAGAAATATAAGAGCCACC**ATGGAGGTGAC
GGCGGACCAGCCGCGCTGGGTGAGCCACCACCACCCCGCCGTGCTCAACGGGCAGCACCCGGACACGCACCACCC
GGGCCTCAGCCACTCCTACATGGACGCGGCGCAGTACCCGCTGCCGGAGGAGGTGGATGTGCTTTTTAACATCGA
CGGTCAAGGCAACCACGTCCCGCCCTACTACGGAAACTCGGTCAGGGCCACGGTGCAGAGGTACCCTCCGACCCA
CCACGGGAGCCAGGTGTGCCGCCCGCCTCTGCTTCATGGATCCCTACCCTGGCTGGACGGCGGCAAAGCCCTGGG
CAGCCACCACACCGCCTCCCCCTGGAATCTCAGCCCCTTCTCCAAGACGTCCATCCACCACGGCTCCCCGGGGCC
CCTCTCCGTCTACCCCCCGGCCTCGTCCTCCTCCTTGTCGGGGGGCCACGCCAGCCCGCACCTCTTCACCTTCCC
GCCCACCCCGCCGAAGGACGTCTCCCCGGACCCATCGCTGTCCACCCCAGGCTCGGCCGGCTCGGCCCGGCAGGA
CGAGAAAGAGTGCCTCAAGTACCAGGTGCCCCTGCCCGACAGCATGAAGCTGGAGTCGTCCCACTCCCGTGGCAG
CATGACCGCCCTGGGTGGAGCCTCCTCGTCGACCCACCACCCCATCACCACCTACCCGCCCTACGTGCCCGAGTA
CAGCTCCGGACTCTTCCCCCCCAGCAGCCTGCTGGGCGGCTCCCCCACCGGCTTCGGATGCAAGTCCAGGCCCAA
GGCCCGGTCCAGCACAGAAGGCAGGGAGTGTGTGAACTGTGGGGCAACCTCGACCCCACTGTGGCGGCGAGATGG
CACGGGACACTACCTGTGCAACGCCTGCGGGCTCTATCACAAAATGAACGGACAGAACCGGCCCCTCATTAAGCC
CAAGCGAAGGCTGTCTGCAGCCAGGAGAGCAGGGACGTCCTGTGCGAACTGTCAGACCACCACAACCACACTCTG
GAGGAGGAATGCCAATGGGGACCCTGTCTGCAATGCCTGTGGGCTCTACTACAAGCTTCACAATATTAACAGACC
CCTGACTATGAAGAAGGAAGGCATCCAGACCAGAAACCGAAAAATGTCTAGCAAATCCAAAAAGTGCAAAAAAGT
GCATGACTCACTGGAGGACTTCCCCAAGAACAGCTCGTTTAACCCGGCCGCCCTCTCCAGACACATGTCCTCCCT
GAGCCACATCTCGCCCTTCAGCCACTCCAGCCACATGCTGACCACGCCCACGCCGATGCACCCGCCATCCAGCCT
GTCCTTTGGACCACACCACCCCTCCAGCATGGTCACCGCCATGGGTTAG**GCTGCCTTCTGCGGGGCTTGCCTTCT**
GGCCATGCCCTTCTTCTCTCCCTTGCACCTGTACCTCTTGGTCTTTGAATAAAGCCTGAGTAGGAAGTAGCATAA
CCCCTTGGGGCCTCTAAACGGGTCTTGAGGGGTTTTTTG

KLF5 isoform 1
TAATACGACTCACTATAGGG**AAATAAGAGAGAAAAGAAGAGTAAGAAGAAATATAAGAGCCACC**ATGGCTACAAG
GGTGCTGAGCATGAGCGCCCGCCTGGGACCCGTGCCCCAGCCGCCGGCGCCGCAGGACGAGCCGGTGTTCGCGCA
GCTCAAGCCGGTGCTGGGCGCCGCGAATCCGGCCCGCGACGCGGCGCTCTTCCCCGGCGAGGAGCTGAAGCACGC
GCACCACCGCCCGCAGGCGCAGCCCGCGCCCGCGCAGGCCCCGCAGCCGGCCCAGCCGCCCGCCACCGGCCCGCG
GCTGCCTCCAGAGGACCTGGTCCAGACAAGATGTGAAATGGAGAAGTATCTGACACCTCAGCTTCCTCCAGTTCC
TATAATTCCAGAGCATAAAAAGTATAGACGAGACAGTGCCTCAGTCGTAGACCAGTTCTTCACTGACACTGAAGG
GTTACCTTACAGTATCAACATGAACGTCTTCCTCCCTGACATCACTCACCTGAGAACTGGCCTCTACAAATCCCA
GAGACCGTGCGTAACACACATCAAGACAGAACCTGTTGCCATTTTCAGCCACCAGAGTGAAACGACTGCCCCTCC

Development • Supplementary information

TCCGGCCCCGACCCAGGCCCTCCCTGAGTTCACCAGTATATTCAGCTCACACCAGACCGCAGCTCCAGAGGTGAA
CAATATTTTCATCAAACAAGAACTTCCTACACCAGATCTTCATCTTTCTGTCCCTACCCAGCAGGGCCACCTGTA
CCAGCTACTGAATACACCGGATCTAGATATGCCCAGTTCTACAAATCAGACAGCAGCAATGGACACTCTTAATGT
TTCTATGTCAGCTGCCATGGCAGGCCTTAACACACACACCTCTGCTGTTCCGCAGACTGCAGTGAAACAATTCCA
GGGCATGCCCCCTTGCACATACACAATGCCAAGTCAGTTTCTTCCACAACAGGCCACTTACTTTCCCCCGTCACC
ACCAAGCTCAGAGCCTGGAAGTCCAGATAGACAAGCAGAGATGCTCCAGAATTTAACCCCACCTCCATCCTATGC
TGCTACAATTGCTTCTAAACTGGCAATTCACAATCCAAATTTACCCACCACCCTGCCAGTTAACTCACAAAACAT
CCAACCTGTCAGATACAATAGAAGGAGTAACCCCGATTTGGAGAAACGACGCATCCACTACTGCGATTACCCTGG
TTGCACAAAAGTTTATACCAAGTCTTCTCATTTAAAAGCTCACCTGAGGACTCACACTGGTGAAAAGCCATACAA
GTGTACCTGGGAAGGCTGCGACTGGAGGTTCGCGCGATCGGATGAGCTGACCCGCCACTACCGGAAGCACACAGG
CGCCAAGCCCTTCCAGTGCGGGGTGTGCAACCGCAGCTTCTCGCGCTCTGACCACCTGGCCCTGCATATGAAGAG
GCACCAGAACTGA**GCTGCCTTCTGCGGGGCTTGCCTTCTGGCCATGCCCTTCTTCTCTCCCTTGCACCTGTACCT**
CTTGGTCTTTGAATAAAGCCTGAGTAGGAAGTAGCATAACCCCTTGGGGCCTCTAAACGGGTCTTGAGGGGTTTT
TTG

MYC
TAATACGACTCACTATAGGG**AAATAAGAGAGAAAAGAAGAGTAAGAAGAAATATAAGAGCCACC**atgcccctcaa
cgttagcttcaccaacaggaactatgacctcgactacgactcggtgcagccgtatttctactgcgacgaggagga
gaacttctaccagcagcagcagcagagcgagctgcagcccccggcgcccagcgaggatatctggaagaaattcga
gctgctgcccaccccgcccctgtcccctagccgccgctccgggctctgctcgccctcctacgttgcggtcacacc
cttctcccttcggggagacaacgacggcggtggcgggagcttctccacggccgaccagctggagatggtgaccga
gctgctgggaggagacatggtgaaccagagtttcatctgcgacccggacgacgagaccttcatcaaaaacatcat
catccaggactgtatgtggagcggcttctcggccgccgccaagctcgtctcagagaagctggcctcctaccaggc
tgcgcgcaaagacagcggcagcccgaaccccgcccgcggccacagcgtctgctccacctccagcttgtacctgca
ggatctgagcgccgccgcctcagagtgcatcgacccctcggtggtcttcccctaccctctcaacgacagcagctc
gcccaagtcctgcgcctcgcaagactccagcgccttctctccgtcctcggattctctgctctcctcgacggagtc
ctccccgcagggcagccccgagcccctggtgctccatgaggagacaccgcccaccaccagcagcgactctgagga
ggaacaagaagatgaggaagaaatcgatgttgtttctgtggaaaagaggcaggctcctggcaaaaggtcagagtc
tggatcaccttctgctggaggccacagcaaacctcctcacagcccactggtcctcaagaggtgccacgtctccac
acatcagcacaactacgcagcgcctccctccactcggaaggactatcctgctgccaagagggtcaagttggacag
tgtcagagtcctgagacagatcagcaacaaccgaaaatgcaccagccccaggtcctcggacaccgaggagaatgt
caagaggcgaacacacaacgtcttggagcgccagaggaggaacgagctaaaacggagcttttttgccctgcgtga
ccagatcccggagttggaaaacaatgaaaaggcccccaaggtagttatccttaaaaaagccacagcatacatcct
gtccgtccaagcagaggagcaaaagctcatttctgaagaggacttgttgcggaaacgacgagaacagttgaaaca
caaacttgaacagctacggaactcttgtgcgtaa**GCTGCCTTCTGCGGGGCTTGCCTTCTGGCCATGCCCTTCTT**
CTCTCCCTTGCACCTGTACCTCTTGGTCTTTGAATAAAGCCTGAGTAGGAAGTAGCATAACCCCTTGGGGCCTCT
AAACGGGTCTTGAGGGGTTTTTTG

TFAP2C isoform 1
TAATACGACTCACTATAGGG**AAATAAGAGAGAAAAGAAGAGTAAGAAGAAATATAAGAGCCACC**ATGTTGTGGAAA
ATAACCGATAATGTCAAGTACGAAGAGGACTGCGAGGATCGCCACGACGGGAGCAGCAATGGGAATCCGCGGGTCCCCCACCT
CTCCTCCGCCGGGCAGCACCTCTACAGCCCCGCGCCACCCCTCTCCCACACTGGAGTCGCCGAATATCAGCCGCCACCCTACT
TTCCCCCTCCCTACCAGCAGCTGGCCTACTCCCAGTCGGCCGACCCCTACTCGCATCTGGGGGAAGCGTACGCCGCCGCCATC
AACCCCCTGCACCAGCCGGCGCCCACAGGCAGCCAGCAGCAGGCCTGGCCCGGCCGCCAGAGCCAGGAGGGAGCGGGGCTGCC
CTCGCACCACGGGCGCCCGGCCGGCCTACTGCCCCACCTCTCCGGGCTGGAGGCGGGCGCGGTGAGCGCCCGCAGGGATGCCT
ACCGCCGCTCCGACCTGCTGCTGCCCCACGCACACGCCCTGGATGCCGCGGGCCTGGCCGAGAACCTGGGGCTCCACGACATG
CCTCACCAGATGGACGAGGTGCAGAATGTCGACGACCAGCACCTGTTGCTGCACGATCAGACAGTCATTCGCAAAGGTCCCAT
TTCCATGACCAAGAACCCTCTGAACCTCCCCTGTCAGAAGGAGCTGGTGGGGGCCGTAATGAACCCCACTGAGGTCTTCTGCT
CAGTCCCTGGAAGATTGTCGCTCCTCAGCTCTACGTCTAAATACAAAGTGACAGTGGCTGAAGTACAGAGGCGACTGTCCCCA
CCTGAATGCTTAAATGCCTCGTTACTGGGAGGTGTTCTCAGAAGAGCCAAATCGAAAAATGGAGGCCGGTCCTTGCGGGAGAA
GTTGGACAAGATTGGGTTGAATCTTCCGGCCGGGAGGCGGAAAGCCGCTCATGTGACTCTCCTGACATCCTTAGTAGAAGGTG
AAGCTGTTCATTTGGCTAGGGACTTTGCCTATGTCTGTGAAGCCGAATTTCCTAGTAAACCAGTGGCAGAATATTTAACCAGA
CCTCATCTTGGAGGACGAAATGAGATGGCAGCTAGGAAGAACATGCTATTGGCGGCCCAGCAACTGTGTAAAGAATTCACAGA
ACTTCTCAGCCAAGACCGGACACCCCATGGGACCAGCAGGCTCGCCCCAGTCTTGGAGACGAACATACAGAACTGCTTGTCTC
ATTTCAGCCTGATTACCCACGGGTTTGGCAGCCAGGCCATCTGTGCCGCGGTGTCTGCCCTGCAGAACTACATCAAAGAAGCC
CTGATTGTCATAGACAAATCCTACATGAACCCTGGAGACCAGAGTCCAGCTGATTCTAACAAAACCCTGGAGAAAATGGAGAA
ACACAGGAAATAA**GCTGCCTTCTGCGGGGCTTGCCTTCTGGCCATGCCCTTCTTCTCTCCCTTGCACCTGTACCTC**
TTGGTCTTTGAATAAAGCCTGAGTAGGAAGTAGCATAACCCCTTGGGGCCTCTAAACGGGTCTTGAGGGGTTTTT
TG

Development • Supplementary information
