# Supplementary material for: Transcription factor-based transdifferentiation of human embryonic to trophoblast stem cells
Source: Development. 2024 Sep 10;151(17):dev202778. doi: 10.1242/dev.202778 (PMC11556314; doi:10.1242/dev.202778)
Supplement: Supplementary file 1 [file develop-151-202778_review_history.pdf]

Transcription factor-based transdifferentiation of human embryonic to trophoblast stem cells

Paula A. Balestrini, Ahmed Abdelbaki, Afshan McCarthy, Liani Devito, Claire E. Senner, Alice E. Chen, Prabhakaran Munusamy, Paul Blakeley, Kay Elder, Phil Snell, Leila Christie, Paul Serhal, Rabi A. Odia, Mahesh Sangrithi, Kathy K. Niakan and Norah M. E. Fogarty
DOI: 10.1242/dev.202778

Editor: Liz Robertson

Review timeline

Original submission:	19 August 2021
Editorial decision:	16 September 2021
Resubmission:	26 February 2024
Editorial decision:	8 April 2024
First revision received:	21 June 2024
Editorial decision:	30 July 2024
Second revision received:	31 July 2024
Accepted:	5 August 2024

Original submission

First decision letter

MS ID#: DEVELOP/2021/200062

MS TITLE: Direct reprogramming of human embryonic to trophoblast stem cells

AUTHORS: Norah ME Fogarty, Ahmed Abdelbaki, Afshan McCarthy, Liani Devito, Alice E Chen, Prabhakaran Munusamy, Paul Blakeley, Kay Elder, Phil Snell, Leila Christie, Paul Serhal, Rabi A Odia, Mahesh Sangrithi, and Kathy K Niakan

I have now received all the referees' reports on the above manuscript, and have reached a decision. The referees' comments are appended below, or you can access them online: please go to BenchPress and click on the 'Manuscripts with Decisions' queue in the Author Area.

As you will see, the referees express interest in your work of deriving TSCs from the PSCs, but have some significant criticisms on the depth of characterization of the induced TSCs and the trophoblast lineage propensity, benchmarking of the induced TSCs against other TSCs and attribute of the inducing factors to the efficiency of reprogramming/trans-differentiation. On these considerations, a substantial revision of your manuscript is recommended before we can consider publication. If you are able to revise the manuscript along the lines suggested, which may involve further experiments, I will be happy receive a revised version of the manuscript. Your revised paper will be re-reviewed by one or more of the original referees, and acceptance of your manuscript will depend on your addressing satisfactorily the reviewers' major concerns. Please also note that Development will normally permit only one round of major revision.

We are aware that you may be experiencing disruption to the normal running of your lab that make experimental revisions challenging. If it would be helpful, we encourage you to contact us to discuss your revision in greater detail. Please send us a point-by-point response indicating where you are able to address concerns raised (either experimentally or by changes to the text) and

© 2024. Published by The Company of Biologists under the terms of the Creative Commons Attribution License (https://creativecommons.org/licenses/by/4.0/).

where you will not be able to do so within the normal timeframe of a revision. We will then provide further guidance. Please also note that we are happy to extend revision timeframes as necessary.

Please attend to all of the reviewers' comments and ensure that you clearly highlight all changes made in the revised manuscript. Please avoid using 'Tracked changes' in Word files as these are lost in PDF conversion. I should be grateful if you would also provide a point-by-point response detailing how you have dealt with the points raised by the reviewers in the 'Response to Reviewers' box. If you do not agree with any of their criticisms or suggestions please explain clearly why this is so.

Reviewer 1

Advance summary and potential significance to field

In this manuscript, the authors reported a novel strategy to establish human induced trophoblast stem cells (iTSCs) from human embryonic stem cells (ESCs). They analyzed transcriptome of the trophectoderm lineage in human pre-implantation embryos and identified transcription factors (TFs) expressing in trophectoderm-lineage specific manner. Then the functions of these TFs, GATA2, GATA3, KLF5, TFAP2C as well as MYC were assessed for their ability to induce iTSCs by inducible overexpression in human ESCs. As the result, they found that the overexpression of these TFs generate human iTSCs. The resulting iTSCs are quite similar to bona fide TSCs in their gene expression patterns and cell biological characters. Examination of the effect of withdrawal of individual factors revealed that the combination of GATA2, GATA3 and MYC is the minimal requirement for generation of human iTSCs. This is the first report for the generation of human iTSCs using defined set of TFs, so has a potential as a candidate for publication in Development after revision.

Comments for the author

1. There is no quantitative data of the reprogramming efficiency. This is especially required in the part of the examination of the effect of withdrawal of individual factors.
2. The authors showed the formation of TSC-like colonies at 20 days after induction of TFs. Why did the differentiation event occur slowly in comparison to the mouse system? It will be good if the authors add the time-course analysis of the reprogramming event.
3. How about the potential of these TFs in reprogramming of somatic cells such as human dermal fibroblasts?
4. How about the potential of these TFs in reprogramming of naïve human ESCs?
5. The authors demonstrated that the minimal set of TFs consists of GATA2, GATA3 and MYC. How do two similar TFs GATA2 and GATA3 differentially act in reprogramming?
6. The role of GATA2, GATA3, TFAP2A and TFAP2C in differentiation of human ESCs to trophectoderm-like cells was reported by Krendl et al (PNAS, 2017).
7. Line 104: The function to induce differentiation of mouse ESCs to TSCs was reported for Tfap2c (Kuckenberg et al, MCB, 2010) and Gata3 (Ralston et al, Development, 2010).

Reviewer 2

Advance summary and potential significance to field

The paper entitled “Direct reprogramming of human embryonic to trophoblast stem cells” by N Fogarty et al. purports to both elucidate important transcription factors in the TE network and establish a novel method for converting primed ESCs into trophoblast stem cells (TSCs). The structure of the paper may be split into two sections, namely the attempts to find a small number of transcription factors which are central to the TSC gene circuitry and the utilization of these factors to convert ESCs to TSC and prove their identity and functionality. Though powerful methods such as RNA-seq and staining of early human embryos are utilized in the first part, they do not prove as informative as they might be in setting the stage for the second section. In the second part, the characterization of the resultant TSC colonies is lacking. In general, given the multiple works which have been recently published which describe generation of iTSCs from ESCs (PMID: 32048992, PMID: 33238118), the lack of an exciting discovery regarding the transgenes utilized here

© 2024. Published by The Company of Biologists under the terms of the Creative Commons Attribution License (https://creativecommons.org/licenses/by/4.0/).

and the superficial characterization of the resultant TSCs and their derivation process, the novelty of the paper is somewhat underwhelming.

Comments for the author

A number of major and minor concerns arise from the work, as follows:
Major concerns:
-At the start of the paper, the authors describe that they performed microdissection of mural TE in order to perform gene expression analysis “to determine which genes overlap in their expression…may be required for the establishment and maintenance of the TE and thereby may facilitate iTSC reprogramming” (137-140). Why was this analysis conducted only on the mural and not on the polar TE? It is known that there are differences in gene expression profiles and functions between the two sub populations (PMID: 31959285), and that there is reason to believe that the human TSC population arises from the polar rather than mural TE, as in the mouse although the implantation process is somehow different between the human and mouse. Furthermore, it is concerning that no experiments were done to assure lack of contamination with other embryonic cell types.

-The classification of the cells into developmental stages looks artificial in the PCA plot (Figure 1B). Embryo 9, which is a day 7 embryo, is much more similar to day 6 embryos than the other day 7 ones (Figure 1B). As such, grouping the embryos in vitro according to day may not reflect the “true” developmental identity of the cells. Perhaps it would be useful to evaluate the developmental stages based on cell surface markers as previously described (PMID: 33831365). This would also address the issue of cell contamination.
-The images of the staining which was performed on cultured embryos (203-223, Figure 2) have a number of issues which need to be addressed. First of all, some panels include NANOG staining and some SOX2. Is there a reason why it is not uniform, as would be expected? Indeed, lines 213-214 read, “Lower levels of nuclear KLF5 expression [...] colocalised with SOX2 expression”. The staining shows NANOG and not SOX2. Furthermore, this and a number of the other written observations are difficult to ascertain in the images, such as cytoplasmic expression of KLF5 at day 5 (211-212, Figure 2C). The quality of the staining and the background signal is dramatically different between different panels (such as with NANOG), even for the same antibody, which impedes the ability to reliably interpret the results.
-Notwithstanding the above comments, it seems that a large portion of the paper is dedicated to describing the search for relevant transcription factors, which eventually does not yield results which would justify taking up half of the manuscript (125-223). Though much information can be derived from work with early human embryos, the experiments presented in this paper largely validate what is already known. Furthermore, the eventual selection of the factors relies only partially on the data generated here, which further highlights the concern that perhaps this section could be significantly minimized and the figures moved to the supplementary section.
-Regarding the derivation of TSCs, there is no quantitative evaluation of TSC formation and therefore it is impossible to evaluate the efficiency of the methods for inducing TSCs. This point becomes especially important in light of the results in figure 6 and S2-S3, as it seems that the main difference between utilizing the 5 factors and GATA2, GATA3 and MYC alone is primarily the efficiency of the process. This might be accomplished by evaluating gene expression in the population in each plate by performing qPCR experiments for TSC markers as well as markers of ESC and their derivatives (endoderm, mesoderm, ectoderm). In a similar vein, in the lentiviral system the representative TSC colony’s morphology is not ideal (Figure 3C), while the staining of the TSC markers seems patchy and inconsistent in multiple panels (Figure 3E,J). It looks as if there may be a mixed population. Therefore, further characterization of the cells in the plate may be informative. This may be accomplished by qPCR experiments as mentioned above, or perhaps flow-cytometry with various characteristic cell-surface markers.
-The authors did not check transgene integration and expression in the lentiviral experiments. As above, the need for this is highlighted by the results in figure 6 and S2-S3. Perhaps not all of the 5 factors integrated in the resultant colonies in these experiments? This would further elucidate the authors’ observations regarding the fact that GATA2, GATA3 and MYC are sufficient in this trans-differentiation process. -Another major issue with this manuscript is the extent of characterization of TSCs. Though some trophoblast markers are examined and differentiation into ST is explored, other characteristics such as miRNA expression and hypomethylation of the ELF5 promotor are not shown (PMID: 26862703), the latter being especially interesting in a transdifferentiation process

© 2024. Published by The Company of Biologists under the terms of the Creative Commons Attribution License (https://creativecommons.org/licenses/by/4.0/).

from ESCs, as this hypomethylation fails to occur in mouse models of ESC to TSC transdifferentiation (PMID: 25423963). Importantly, differentiation into extravillous trophoblast (EVTs) is not mentioned. Since EVT function is central in the development of various placental dysfunctions (PMID: 28369440, PMID: 21388889), the capability to differentiate into these cell types should be assessed. Was there an attempt to differentiate these cells into EVTs? Is the differentiation capacity affected by the factors used? Another potential issue is checking markers of amnion, as recent works have asserted that some confusion is prevalent between characterizing trophoblast versus amnion cells, even in cells with the capacity to form ST-like syncytia (PMID: 33831365, https://doi.org/10.1101/2021.06.28.450118). Regarding ST differentiation, it is odd that in Figure 5B line 1 there is no detectable hCG in the undifferentiated TSCs, while other works show a high level of hCG expression in these cells (PMID: 29249463, table S1). Indeed, some syncytia may be observed in the panel of stained undifferentiated cells in Figure 5A. As such, detection of hCG in the culture medium is not optimally informative and expression of more markers of ST should be examined and compared with undifferentiated cells, such as CSH1, GCM1, SDC1, and PSG1, for example.
-Lines 274-275 purport that PC1 and PC2 in the PCA analysis in Figure 4A account for 95% of the variance between the groups of cells. Although this is true, PC1, which is 85% of the variance, seems to separate only between primary isolated vCTB and the cultured cells (iTSCs and ESCs), so that most of the information in this graph likely reflects the effects of culturing cells in-vitro, which is not helpful in contributing insight to this study. The authors should add PCA plots excluding the vCTBs, so that it does not mask more subtle differences between the iTSCs and ESCs.
-The centrality of GATA proteins for the induction of trophoblast state from human ESCs is not novel, as GATA3 overexpression was previously shown to induce a trophoblast phenotype in primed ESCs (PMID: 29078328). This being said, it becomes especially pertinent to examine specifically the functionality of the colonies as TSCs, rather than simply having a trophoblast identity. As such, it would be useful to isolate the colonies generated by GATA2, GATA3 and MYC and examine their proliferative capacity as well as their ability to differentiate into ST and EVT.
-Given the issue of novelty in this study, the authors should expand on why this method is an important improvement over the methods which already exist for generating TSCs from pluripotent cells. Minor concerns:
-Generation of hTSCs from hESCs is a trans-differentiation process and not reprogramming, as continually referred to in the text. Reprogramming reverts differentiated cells into pluripotency, while in transdifferentiation, cells regress to a point in which it is possible to switch lineages, or are directly converted to a different cell identity (PMID: 21252997).
-The projection image of the staining at day 7 in Figure 2C seems flipped
-The authors should provide an explanation for the difference in the passages of the resulting iTSCs (lines 242, 265)
- Did the cells start to differentiate? Did they become less proliferative?-Line 318 includes a callout to Fig S4:A, the correct callout is S3

Reviewer 3

Advance summary and potential significance to field

Forgaty et al. identified human preimplantation TE transcription factors (GATA2, GATA3, TFAP2C and KLF5) to convert hESCs to iTSCs and revealed that the transient expression of these transcription factors along with MYC can establish human iTSCs using a doxycycline-inducible lentivirus or mRNA transfection. The iTSCs could be maintained stably in Okae's condition and showed similar gene expression as blastocyst- or primary placenta-derived hTSCs (Okae et al., Cell Stem Cell 2018).

I appreciate the authors' efforts to establish the transgene induction system for transdifferentiating primed hESCs to iTSCs. However, Thomson's group first reported that primed hESCs differentiate into trophoblast lineage by BMP4 (Xu et al., Nat Biotechnol, 2002), and others reported such ability of primed hESCs using modified protocols containing BMP4, A83-01, and/or PD173074 (Li et al., Development, 2013; Amita et al., PNAS, 2013; Sarker et al., JBC, 2015; Yang et al., PNAS, 2015) or a two-step protocol of BMP4 and IWP2 (Horii et al., Curr. Protoc. Stem Cell Biol, 2019). These cells can be maintained under A83-03, CHIR99021, and EGF for a long time (Io et al., Cell Stem Cell 2021).

© 2024. Published by The Company of Biologists under the terms of the Creative Commons Attribution License (https://creativecommons.org/licenses/by/4.0/).

Moreover, there is controversy regarding whether primed hESCs induce true trophoblast cells. Indeed, recent reports suggested that primed hESC-derived trophoblast cells deviate from trophoblast cells in vivo (Bernardo et al., Cell Stem Cell 2011; Io et al., Cell Stem Cell 2021; Guo et al., Cell Stem Cell 2021). Therefore, if the authors can show that their transgene-induced trophoblast stem cells are different from current primed PSC-derived trophoblast-like cells and similar to in vivo cytotrophoblast cells, then the novelty and significance will rise significantly.

Comments for the author

There is controversy regarding primed PSC-derived trophoblast-like cells as described in above. Based on such arguments, four criteria for trophoblast were proposed (Lee et al., Cell Reports 2016). For the study to be published in Development, the authors should show 5F-iTSCs satisfy these four trophoblast criteria and are different from primed hESC-derived TS cells induced by chemical compounds. The following points need to be addressed.
1.Trophoblast criteria
Do 5F-iTSCs satisfy the trophoblast criteria reported by Lee et al.?
2.Efficiency of 5F-iTSCs
How efficiently are 5F-iTSCs induced from primed hESCs?
3.5F-iTSC transdifferentiation
As the authors mentioned, trophoblast development starts from trophectoderm (TE) of blastocysts. After implantation, TE differentiates into cytotrophoblast. Human trophoblast stem cells (TSCs) are captured as a counterpart of cytotrophoblast at the post-implantation stage. The authors claimed that their cells have similar gene expression profiles as Okae's hTS cells, which suggests the cells are cytotrophoblast-like cells at post-implantation. Although the authors identified GATA2, GATA3, TFAP2C, and KLF5 as TE markers, they did not compare their cells to TE but to primary cytotrophoblast at post-implantation and to other reported hTS cells. Are 5F-iTSCs similar to TE? Also, were primed hESCs transiently induced to the pre-implantation TE-like state and then differentiated into the post-implantation cytotrophoblast state? Or, did they differentiate into the cytotrophoblast-like state via amnion-like state?
4.The bright field image of Stable iTSC (Fig 3d) looks very heterogeneous. Are all of the cells shown TSCs? The authors should show flow cytometry results for surface markers reported previously (Okae et al., 2018; Io et al., 2021).
5.I think the word “reprogramming” is not an appropriate description of the induction of TS cells from ES cells. I suggest “transdifferentiation” or “induction”.
6.Fig. 4A shows that TSCs and primary CTBs are segregated by PC1. What are the loading genes of PC1?

Response to Reviewers

Reviewer 1 Advance Summary and Potential Significance to Field:
In this manuscript, the authors reported a novel strategy to establish human induced trophoblast stem cells (iTSCs) from human embryonic stem cells (ESCs). They analyzed transcriptome of the trophectoderm lineage in human pre-implantation embryos and identified transcription factors (TFs) expressing in trophectoderm-lineage specific manner. Then the functions of these TFs, GATA2, GATA3, KLF5, TFAP2C as well as MYC were assessed for their ability to induce iTSCs by inducible overexpression in human ESCs. As the result, they found that the overexpression of these TFs generate human iTSCs. The resulting iTSCs are quite similar to bona fide TSCs in their gene expression patterns and cell biological characters. Examination of the effect of withdrawal of individual factors revealed that the combination of GATA2, GATA3 and MYC is the minimal requirement for generation of human iTSCs. This is the first report for the generation of human iTSCs using defined set of TFs, so has a potential as a candidate for publication in Development after revision.

Reviewer 1 Comments for the Author:
1. There is no quantitative data of the reprogramming efficiency. This is especially required in the part of the examination of the effect of withdrawal of individual factors.
We thank the reviewer for this suggestion. A quantitative analysis of iTSC transdifferentiation has been included in the revised manuscript. We repeated the 5-factor transdifferentiation and

© 2024. Published by The Company of Biologists under the terms of the Creative Commons Attribution License (https://creativecommons.org/licenses/by/4.0/).

the transcription factor combinatorial/omission experiments using the mRNA transfection approach. We performed immunofluorescence analysis for the detection of TP63- and KRT18-positive cells, as a read-out of successfully transdifferentiated cells. TP63 is a lineage marker of cytotrophoblast cells and a marker of hTSCs (Haider et al., Okae et al.) and KRT18 is an epithelial marker that distinguishes between hTSCs and hESCs (Naama et al. 2023). To quantify transdifferentiated cells an average of 250 (Figure 8) or 150 (Figure S3) cells from 5 random frames of view were counted from two independent experiments. Quantification of immunofluorescence analyses revealed that approximately 80% of cells were KRT18- and TP63-positive when treated with the 5-factor cocktail of GATA2, GATA3, TFAP2C, KLF5 and MYC (Fig. 8C). Efficiency of transdifferentiation was reduced to about 40% of total cells when the 3-factor cocktail of GATA2, GATA3 and MYC was administered.

2. The authors showed the formation of TSC-like colonies at 20 days after induction of TFs. Why did the differentiation event occur slowly in comparison to the mouse system? It will be good if the authors add the time-course analysis of the reprogramming event.
We followed the strategy by Benchetrit et al. 2015, where in the mouse they induced factors for 20 days in the presence of doxycycline, followed by 10 days of culture in the absence of doxycycline. Consistent with this, in our human system we have shown that 20 days of 5F transcription factor overexpression leads to successful transdifferentiation. In the manuscript we have clarified that this is the approach we used (lines 287-289). Moreover, in response to reviewer 2 and 3 we have performed a time-course RT-qPCR analysis during the 5F transdifferentiation. We assessed expression of trophoblast-associated transcripts in induced cells relative to cells grown in hTSC media alone. Here we demonstrate that while at day 15 we get significant upregulation of endogenous *GATA3*, *TP63* and *ENPEP*, it is at day 20 that we also have significant upregulation of *NR2F2* and trophoblast-associated cell surface markers (lines 289-297, Figure 3C). This suggested to us that 20 days of overexpression is required to induce the downstream trophoblast network. In the mRNA combinatorial transdifferentiation we used the strategy of Kubaczka et al. 2015 and overexpressed for 10 days. The rationale for this was to reduce the total amount of mRNA needed for all experiments as it is labour intensive and expensive to generate these mRNAs. Indeed here our data confirms that 10 days of induction is sufficient to induce the trophoblast programme with 80% of cells showing KRT18- and TP63-upregulation (lines 345, Figure 8). However, we did not derive stable lines from 5F-iTSCs generated after only 10 days of 5F overexpression, but we would hypothesise that this approach would also be successful.

3. How about the potential of these TFs in reprogramming of somatic cells such as human dermal fibroblasts?
We attempted this several times using the mRNA protocol and were unsuccessful. This may suggest we are missing factors which would be interesting to explore in the future. This is included in the discussion (lines 618-620).

4. How about the potential of these TFs in reprogramming of naïve human ESCs?
Indeed, as we cover in the discussion (lines 503-512), studies have shown that naïve hESCs make the transition to hTSCs when cultured in hTSC media without the requirement for forced expression of transcription factors. hTSCs-derived from naïve hESCs were included in our transcriptional analysis. The PCA plot indicates that iTSCs generated in this study cluster with naïve-derived hTSCs, existing hTSCs (Okae et al. 2018), and primary cytotrophoblast cells. We would hypothesise that overexpression of these 5F would also transdifferentiation naïve cells to iTSCs.

5. The authors demonstrated that the minimal set of TFs consists of GATA2, GATA3 and MYC. How do two similar TFs GATA2 and GATA3 differentially act in reprogramming?
The reviewer raises an important question. Interestingly in our time-course analysis of transdifferentiation, the timing of onset of endogenous *GATA2*, as well as the levels to which it is upregulated, appears to lag that of *GATA3* expression. This may indicate that while both are required, GATA3 is upstream of GATA2. We are interested in exploring in the future through using methods to elucidate transcription factor binding and chromatin occupancy sites of GATA2 and GATA3 to distinguish the roles of these factors. This is discussed in lines 561-573.

© 2024. Published by The Company of Biologists under the terms of the Creative Commons Attribution License (https://creativecommons.org/licenses/by/4.0/).

6. The role of GATA2, GATA3, TFAP2A and TFAP2C in differentiation of human ESCs to trophectoderm-like cells was reported by Krendl et al (PNAS, 2017).

This reference has been included in lines 473-475. Indeed as the referee states whilst GATA2, GATA3 and TFAP2C, along with TFAP2A, have been previously reported to differentiate hESCs to trophectoderm-like cells, these studies were not successful in establishing stable lines that could be propagated in culture and subjected to directed differentiation (Krendl et al., 2017).

7. Line 104: The function to induce differentiation of mouse ESCs to TSCs was reported for Tfap2c (Kuckenberg et al, MCB, 2010) and Gata3 (Ralston et al, Development, 2010).

The known roles for these factors in mouse are discussed as part of our criteria for identifying candidate transdifferentiation factors. We have cited these references (Lines 229-231).

***** Reviewer 2 Advance Summary and Potential Significance to Field: The paper entitled “Direct reprogramming of human embryonic to trophoblast stem cells” by N Fogarty et al. purports to both elucidate important transcription factors in the TE network and establish a novel method for converting primed ESCs into trophoblast stem cells (TSCs). The structure of the paper may be split into two sections, namely the attempts to find a small number of transcription factors which are central to the TSC gene circuitry and the utilization of these factors to convert ESCs to TSC and prove their identity and functionality. Though powerful methods such as RNA-seq and staining of early human embryos are utilized in the first part, they do not prove as informative as they might be in setting the stage for the second section. In the second part, the characterization of the resultant TSC colonies is lacking. In general, given the multiple works which have been recently published which describe generation of iTSCs from ESCs (PMID: 32048992, PMID: 33238118), the lack of an exciting discovery regarding the transgenes utilized here and the superficial characterization of the resultant TSCs and their derivation process, the novelty of the paper is somewhat underwhelming.

Reviewer 2 Comments for the Author:
A number of major and minor concerns arise from the work, as follows: Major concerns:

At the start of the paper, the authors describe that they performed microdissection of mural TE in order to perform gene expression analysis “to determine which genes overlap in their expression…may be required for the establishment and maintenance of the TE and thereby may facilitate iTSC reprogramming” (137-140). Why was this analysis conducted only on the mural and not on the polar TE? It is known that there are differences in gene expression profiles and functions between the two sub populations (PMID: 31959285), and that there is reason to believe that the human TSC population arises from the polar rather than mural TE, as in the mouse although the implantation process is somehow different between the human and mouse. Furthermore, it is concerning that no experiments were done to assure lack of contamination with other embryonic cell types.
As the reviewer states, our RNA-seq analysis was performed on mural TE and did not capture the polar TE population. We reasoned that bulk RNA-seq was more appropriate over single cell RNA-seq for our aim of identifying transcription factors because this method is more suited for identifying lowly expressed genes, including transcription factors, which often drop out from single cell RNA-sequencing data (Kharchenko et al., 2014). This approach had the additional and significant benefit of minimising the numbers of embryos used in research. Microdissecting the mural TE also allowed us to visually assess that we did not have contamination from the inner cell mass. We did not observe expression of molecular markers associated with the epiblast or primitive endoderm, such as *NANOG* or *SOX17*, in TE RNA-seq samples confirming we did not have contamination from inner cell mass cells. This is clarified **in line 167-174**

While the literature describes some molecular differences between polar and mural TE, we currently do not know where TSCs reside in the human blastocyst. However, we know that markers that are associated with hTSCs, including GATA3, GATA2, KRT18, TEAD4 and VGLL1, are expressed throughout the TE from the time of TE initiation at the morula stage, and persist throughout the TE (both mural and polar) in the blastocyst (Gerri et al. 2020; VGLL1

© 2024. Published by The Company of Biologists under the terms of the Creative Commons Attribution License (https://creativecommons.org/licenses/by/4.0/).

unpublished). Thus we hypothesised that the mural TE was likely to have a transcription factor network similar to that of the target TSCs. Interesting, existing hTSCs more closely resemble the post-implantation cytotrophoblast cells. In all, this suggests that further work is required to optimise the in vitro signalling environment to more closely recapitulate the preimplantation TE niche. Existing cellular transdifferentiation models could be used to perform high-throughput media screening experiments and resultant cells assessed against primary cytotrophoblast and preimplantation TE. This is discussed in lines 550-558.

The classification of the cells into developmental stages looks artificial in the PCA plot (Figure 1B). Embryo 9, which is a day 7 embryo, is much more similar to day 6 embryos than the other day 7 ones (Figure 1B). As such, grouping the embryos in vitro according to day may not reflect the "true" developmental identity of the cells. Perhaps it would be useful to evaluate the developmental stages based on cell surface markers as previously described (PMID: 33831365). This would also address the issue of cell contamination.

We have removed the grouping circles from the figure (Figure 1B). We kept the groupings based on data from clinic and clarified this in line 649. lo et al. (PMID: 33831365) described *ENPEP* to be increased in TE of Day 7 blastocysts compared with Day 5 and Day 6, however in our datasets *ENPEP* was equally expressed all our samples analysed, and so could not be used to stage TE.

The images of the staining which was performed on cultured embryos (203-223, Figure 2) have a number of issues which need to be addressed. First of all, some panels include NANOG staining and some SOX2. Is there a reason why it is not uniform, as would be expected? Indeed, lines 213-214 read, "Lower levels of nuclear KLF5 expression [...] colocalised with SOX2 expression". The staining shows NANOG and not SOX2. Furthermore, this and a number of the other written observations are difficult to ascertain in the images, such as cytoplasmic expression of KLF5 at day 5 (211-212, Figure 2C). The quality of the staining and the background signal is dramatically different between different panels (such as with NANOG), even for the same antibody, which impedes the ability to reliably interpret the results.

Embryos are all stained in batches with the same antibody master mixes, and imaged with the same laser settings and same exposure. There is variation where the ICM is located and due to embryo rolling we cannot control where it is located within the Z-stack. We identified the ICM using either SOX2 or NANOG, which are standard epiblast molecular markers. SOX2 or NANOG were chosen, depending on the combinations of antibodies being used. We don't believe there is a justification to use more embryos for IF.

Notwithstanding the above comments, it seems that a large portion of the paper is dedicated to describing the search for relevant transcription factors, which eventually does not yield results which would justify taking up half of the manuscript (125-223). Though much information can be derived from work with early human embryos, the experiments presented in this paper largely validate what is already known. Furthermore, the eventual selection of the factors relies only partially on the data generated here, which further highlights the concern that perhaps this section could be significantly minimized and the figures moved to the supplementary section.
Similarities and differences between day 5, 6 and 7 mural TE have not been previously reported. We therefore feel that our data and analysis provides a data-mining resource for the field. If reviewer feels strongly, we are happy to move this into supplementary.

Regarding the derivation of TSCs, there is no quantitative evaluation of TSC formation and therefore it is impossible to evaluate the efficiency of the methods for inducing TSCs. This point becomes especially important in light of the results in figure 6 and S2-S3, as it seems that the main difference between utilizing the 5 factors and GATA2, GATA3 and MYC alone is primarily the efficiency of the process. This might be accomplished by evaluating gene expression in the population in each plate by performing qPCR experiments for TSC markers as well as markers of ESC and their derivatives (endoderm, mesoderm, ectoderm).

© 2024. Published by The Company of Biologists under the terms of the Creative Commons Attribution License (https://creativecommons.org/licenses/by/4.0/).

In a similar vein, in the lentiviral system the representative TSC colony's morphology is not ideal (Figure 3C), while the staining of the TSC markers seems patchy and inconsistent in multiple panels (Figure 3E,J). It looks as if there may be a mixed population. Therefore, further characterization of the cells in the plate may be informative. This may be accomplished by qPCR experiments as mentioned above, or perhaps flow-cytometry with various characteristic cell-surface markers.

We appreciate the reviewer's helpful suggestions. We have addressed this with further experiments and analysis. Quantification of transdifferentiation was performed and data is presented in Fig. 8, Fig S2, and Fig S3. This data is described above in response to reviewer 1. Indeed we see differences in transdifferentiation efficiency between 5F and 3F transdifferentiation (80% v 40%). However our more extensive analysis of 3F programmed iTSCs shows that these cells cannot be maintained long term in culture, which is a significant difference compared with the 5F iTSCs
Indeed it is a mixed population at day 20. Similar to iPS reprogramming, colonies must be isolated and cleaned up over multiple passages. The lentiviral system was used to validate the timing of transcription factor overexpression required for transdifferentiation. We repeated transdifferentiation using mRNA approach and show in Fig 4E widespread expression of TSC-markers. The mRNA-transdifferentiated iTSCs are those that we analysed further in Fig. 4, Fig. 5, Fig. 6 and Fig. 7.

The authors did not check transgene integration and expression in the lentiviral experiments. As above, the need for this is highlighted by the results in figure 6 and S2-S3. Perhaps not all of the 5 factors integrated in the resultant colonies in these experiments? This would further elucidate the authors' observations regarding the fact that GATA2, GATA3 and MYC are sufficient in this trans-differentiation process.

We designed qPCR primers that distinguish between endogenous and exogenous factors. Primer sequences have been included in table S6 and the method used to assess engineered cell lines are clarified in lines 776-781. Primers for the detection of endogenous expression were designed to span the 3'UTR as this is absent in transgenes.

Another major issue with this manuscript is the extent of characterization of TSCs. Though some trophoblast markers are examined and differentiation into ST is explored, other characteristics such as miRNA expression and hypomethylation of the ELF5 promotor are not shown (PMID: 26862703), the latter being especially interesting in a transdifferentiation process from ESCs, as this hypomethylation fails to occur in mouse models of ESC to TSC transdifferentiation (PMID: 25423963).
We appreciate the reviewer's suggestion and this data has now been included in Fig. 4G and H. We show that 5F-iTSCs expressed C19MC miRNAs to levels equivalent to those detected in existing hTSCs. We show that 5F-iTSCs are hypomethylated at the ELF5 promoter, similar to existing hTSCs.
Importantly, differentiation into extravillous trophoblast (EVTs) is not mentioned. Since EVT function is central in the development of various placental dysfunctions (PMID: 28369440, PMID: 21388889), the capability to differentiate into these cell types should be assessed. Was there an attempt to differentiate these cells into EVTs? Is the differentiation capacity affected by the factors used?
The reviewer makes a good point and this data has now been included in Fig. 7. We confirm that 5F-iTSCs differentiate to EVTs equivalently to existing hTSCs based on morphology. We also performed qRT-PCR analysis for the detection of markers of EVTs and show significant upregulation compared to undifferentiated 5F-iTSCs. We were unable to stabilise the 3F- iTSC line nor subject it to EVT differentiation. This is described in lines 483-441.
Another potential issue is checking markers of amnion, as recent works have asserted that some confusion is prevalent between characterizing trophoblast versus amnion cells, even in cells with the capacity to form ST-like syncytia (PMID: 33831365, https://doi.org/10.1101/2021.06.28.450118). Regarding ST differentiation, it is odd that in Figure 5B line 1 there is no detectable hCG in the undifferentiated TSCs, while other works show a high level of hCG expression in these cells (PMID: 29249463, table S1). Indeed, some syncytia may be observed in the panel of stained undifferentiated cells in Figure

© 2024. Published by The Company of Biologists under the terms of the Creative Commons Attribution License (https://creativecommons.org/licenses/by/4.0/).

5A. As such, detection of hCG in the culture medium is not optimally informative and expression of more markers of ST should be examined and compared with undifferentiated cells, such as CSH1, GCM1, SDC1, and PSG1, for example.

Indeed there are differences in distinguishing amnion from trophoblast as the cell types show some shared gene expression. We performed RT-qPCR analysis for the detection of markers of amnion and we do not detect a difference in the expression between 5F-iTSCs and existing hTSCs (Fig 4F). This is discussed in lines 343-345. We have more fully characterised the syncytiotrophoblast (STBs) generated from 5F-iTSCs and performed RT- qPCR analysis for the detection of a panel of STB markers. We confirm significant upregulation in STB-differentiated 5F-iTSCs compared with undifferentiated 5F-iTSCs (Fig 6C). We have updated the hCG secretion assay to show the comparison between our 5F- iTSCs and existing hTSCs. hCG is the standard assay used to detect successful syncytialisation. It is unclear, biologically, why secreted hCG is detected in undifferentiated hTSCs.

Lines 274-275 purport that PC1 and PC2 in the PCA analysis in Figure 4A account for 95% of the variance between the groups of cells. Although this is true, PC1, which is 85% of the variance, seems to separate only between primary isolated vCTB and the cultured cells (iTSCs and ESCs), so that most of the information in this graph likely reflects the effects of culturing cells in-vitro, which is not helpful in contributing insight to this study. The authors should add PCA plots excluding the vCTBs, so that it does not mask more subtle differences between the iTSCs and ESCs.

We thank the reviewer for this comment. We have re-analysed the data by including the TE samples. We now see that 3 clusters are formed: 1. 5F-iTSCs, primary cytotrophoblast cells, all existing hTSC, 2. Primary TE and 3. hESCs. Accordingly we no longer see this separation on the basis of in vitro/in vivo differences.

The centrality of GATA proteins for the induction of trophoblast state from human ESCs is not novel, as GATA3 overexpression was previously shown to induce a trophoblast phenotype in primed ESCs (PMID: 29078328). This being said, it becomes especially pertinent to examine specifically the functionality of the colonies as TSCs, rather than simply having a trophoblast identity. As such, it would be useful to isolate the colonies generated by GATA2, GATA3 and MYC and examine their proliferative capacity as well as their ability to differentiate into ST and EVT.

We repeated the 3F transdifferentiation experiments and attempted to derive stable lines. Interestingly we were unable to grow 3F-iTSCs beyond 5 passages and we observed cell vaculoation, loss of colony integrity, loss of proliferation, and cells eventually lifted off. Thus we could not test their differentiation potential. We have expanded on this in figures 8 D, E and F and in the manuscript on lines 438-441.

Given the issue of novelty in this study, the authors should expand on why this method is an important improvement over the methods which already exist for generating TSCs from pluripotent cells.
We have re-worded the manuscript to state more clearly how this method is an improvement (lines 491-501). This study is the first to show that primed hESCs can be used to generate TSCs that can be maintained in culture and fulfil the criteria set out by Lee et al. for assessing trophoblast identity. It had been previously thought that primed hESCs have lost the potential to generate trophectoderm-like cells, when compared with naïve hESCs (Gou et al. 2021). The method presented here may be beneficial over strategies using naïve cells if it is found that epigenetic imprints are lost during iTSC generation depending on the starting cell type or if there are persistent issues of karyotypic instability in the starting cell type.

Minor concerns:
Generation of hTSCs from hESCs is a trans-differentiation process and not reprogramming, as continually referred to in the text. Reprogramming reverts differentiated cells into pluripotency, while in transdifferentiation, cells regress to a point in which it is possible to switch lineages, or are directly converted to a different cell identity (PMID: 21252997).

© 2024. Published by The Company of Biologists under the terms of the Creative Commons Attribution License (https://creativecommons.org/licenses/by/4.0/).

The reviewer makes a good point and we have revised the manuscript to refer to transdifferentiation.

-The projection image of the staining at day 7 in Figure 2C seems flipped
We thank the reviewer for point this out. This error has been amended.
-The authors should provide an explanation for the difference in the passages of the resulting iTSCs (lines 242, 265) - Did the cells start to differentiate? Did they become less proliferative?
5F-iTSCs could be maintained for more than 20 passages in culture. However, 3F-iTSCs could not be maintained beyond passage 5. Instead, we observed cell vaculoation, loss of colony integrity, and a loss of proliferation with cells eventually lifting off.

-Line 318 includes a callout to Fig S4:A, the correct callout is S3
We thank the reviewer for pointing this out and have updated the figure callouts throughout.
***** Reviewer 3 Advance Summary and Potential Significance to Field:

Forgaty et al. identified human preimplantation TE transcription factors (GATA2, GATA3, TFAP2C and KLF5) to convert hESCs to iTSCs and revealed that the transient expression of these transcription factors along with MYC can establish human iTSCs using a doxycycline-inducible lentivirus or mRNA transfection. The iTSCs could be maintained stably in Okae's condition and showed similar gene expression as blastocyst- or primary placenta-derived hTSCs (Okae et al., Cell Stem Cell 2018).

I appreciate the authors' efforts to establish the transgene induction system for transdifferentiating primed hESCs to iTSCs. However, Thomson's group first reported that primed hESCs differentiate into trophoblast lineage by BMP4 (Xu et al., Nat Biotechnol, 2002), and others reported such ability of primed hESCs using modified protocols containing BMP4, A83-01, and/or PD173074 (Li et al., Development, 2013; Amita et al., PNAS, 2013; Sarker et al., JBC, 2015; Yang et al., PNAS, 2015) or a two- step protocol of BMP4 and IWP2 (Horii et al., Curr. Protoc. Stem Cell Biol, 2019). These cells can be maintained under A83-03, CHIR99021, and EGF for a long time (Io et al., Cell Stem Cell 2021). Moreover, there is controversy regarding whether primed hESCs induce true trophoblast cells. Indeed, recent reports suggested that primed hESC- derived trophoblast cells deviate from trophoblast cells in vivo (Bernardo et al., Cell Stem Cell 2011; Io et al., Cell Stem Cell 2021; Guo et al., Cell Stem Cell 2021). Therefore, if the authors can show that their transgene-induced trophoblast stem cells are different from current primed PSC-derived trophoblast-like cells and similar to in vivo cytotrophoblast cells, then the novelty and significance will rise significantly.

The reviewer is correct in that the identity of trophoblast-like cells generated from primed hESCs by modulating the signalling environment has been under investigation for the last two decades. Many studies, including those detailed above, have applied different culture conditions to primed hESCs. While different markers of trophoblast have been upregulated in different conditions, no study has comprehensively demonstrated that the cells fulfil the criteria of trophoblast as set out by Lee et al. including GATA2, GATA3, TFAP2C expression, *ELF5* promoter methylation and C19MC miRNA expression, and in the cases that differentiation has been demonstrated, the resultant STBs and extravillous trophoblast cells (EVTs) have not been robustly characterised.

<u>Primed hESC + BMP4</u>
Xu et al. did not assess hallmarks of trophoblast including ELF5 promoter methylation, C19MC miRNA expression, nor ability to differentiate. More recently it was shown that a 48 h pretreatment of primed hESCs with BMP4 before culturing in hTSC media generates hTSC-like cells however these cells cannot be maintained in culture, nor be directed to differentiate efficiently to STB or EVTs (Kobayashi et al. 2022). Indeed it has also been shown that BMP4 treatment of hESCs generates cells that also express markers of mesoderm (Bernardo et al. 2011).

<u>Primed hESCs + BMP4, A83-01, and/or PD173074</u>
Amita et al. and Li et al. did not assess hallmarks of trophoblast including *ELF5* promoter methylation, C19MC miRNA expression, and they did not fully characterise differentiation attempts. Thus it is inconclusive whether the cells generated are TSCs. Sarker et al. did not

© 2024. Published by The Company of Biologists under the terms of the Creative Commons Attribution License (https://creativecommons.org/licenses/by/4.0/).

assess GATA2, GATA3 or TFAP2C expression. While they did assess *ELF5* methylation, they found only a reduction to 30% methylation. They also observed increased BRACHYURY expression. The study by Sarkar et al. has been suggested by others to have generated a cell type more closer to amnion rather than trophoblast. Indeed, in the study by Io et al. they found that although primed PSCs differentiated into trophoblast-like cells, the cells were distinct from hTSCs and instead exhibiting properties consistent with the amnion. This was in contrast to the naïve cells which under the same conditions generated TSCs more closely recapitulated existing hTSCs and primary cytotrophoblast.

<u>Primed hESCs + BMP4 + IWP2</u>

Horii et al. did not assess TSC-derivation attempts using trophoblast-specific markers set out by Lee et al. (i.e. GATA2, GATA3 and TFAP2C). They did not assess the other hallmarks of trophoblast including *ELF5* promoter methylation and C19MC miRNA expression. They also noted that the cells generated were "not highly proliferative and could not be propagated under these culture conditions."

In all, our study is the first to show that primed hESC can be used to generate hTSCs that fulfil the criteria set out by Lee et al., can be maintained long-term in culture, and can be directed to differentiate into both STB and EVT. We also show that on the basis of global transcriptional analysis that our 5F-iTSCs closely resemble the primary cytotrophoblast and previously published datasets of existing hTSCs generated from primary cells or from naïve hESCs.

Reviewer 3 Comments for the Author:

There is controversy regarding primed PSC-derived trophoblast-like cells as described in above. Based on such arguments, four criteria for trophoblast were proposed (Lee et al., Cell Reports 2016). For the study to be published in Development, the authors should show 5F- iTSCs satisfy these four trophoblast criteria and are different from primed hESC-derived TS cells induced by chemical compounds. The following points need to be addressed.

1. Trophoblast criteria

Do 5F-iTSCs satisfy the trophoblast criteria reported by Lee et al.? **The trophoblast criteria reported by Lee et al. include expression of trophoblast-specific markers including TFAP2C, GATA2 and GATA3, *ELF5* promoter hypomethylation, and expression of C19MC miRNAs. We appreciate the reviewer's question and have now addressed this by evaluating and showing that the 5F-iTSCs are hypomethylated at the *ELF5* promoter, express the 4 trophoblast-specific miRNAs from the C19MC cluster, and can be differentiated into both extravillous trophoblast cells and syncytiotrophoblast, all equivalently to existing hTSCs from Okae et al. This data is presented in Fig. 4F, G, Fig. 6 and Fig. 7.**

2. Efficiency of 5F-iTSCs

How efficiently are 5F-iTSCs induced?

A quantitative analysis of TSC transdifferentiation has been included in the revised manuscript. We repeated the 5-factor transdifferentiation using the mRNA transfection approach, as well as the transcription factor combinatorial/omission experiments. We performed immunofluorescence analysis for the detection of TP63 and KRT18-positive cells, as a read-out of successfully transdifferentiation cells. An average of 250 (Figure 8) or 150 (Figure S3) cells from 5 random frames of view were counted from two independent experiments. Quantification of immunofluorescence analyses revealed that approximately 80% of cells were KRT18- and TP63-positive when treated with the 5-factor cocktail of GATA2, GATA3, TFAP2C, KLF5 and MYC (Fig. 8C). Efficiency of transdifferentiation was reduced to about 40% of total cells when the 3-factor cocktail of GATA2, GATA3 and MYC was administered.

3. 5F-iTSC differentiation

As the authors mentioned, trophoblast development starts from trophectoderm (TE) of blastocysts. After implantation, TE differentiates into cytotrophoblast. Human trophoblast stem cells (TSCs) are captured as a counterpart of cytotrophoblast at the post-implantation stage. The authors claimed that their cells have similar gene expression profiles as Okae's hTS cells, which suggests the cells are cytotrophoblast-like cells at post-implantation. Although the authors identified GATA2, GATA3, TFAP2C, and KLF5 as TE markers, they did not compare their cells to TE but to primary cytotrophoblast at post-implantation and to other reported hTS cells. Are 5F-iTSCs similar to TE?

© 2024. Published by The Company of Biologists under the terms of the Creative Commons Attribution License (https://creativecommons.org/licenses/by/4.0/).

We thank the reviewer for these comments. We have re-analysed the transcriptional data and now include the TE samples. Using Principal Component Analysis we see that 3 clusters are formed: 1. 5F-iTSCs, primary cytotrophoblast cells, existing hTSCs from previously published datasets 2. Primary TE, and 3. hESCs. Thus our 5F-iTSCs, as well as all hTSC lines analysed, while being transcriptionally indistinguishable from the cytotrophoblast remain distinct from the TE. This suggests that further optimisation is needed to established in vitro stem cell models of pre-implantation TE cells. We have expanded on this point in the discussion in lines 550-558.

Also, were primed hESCs transiently induced to the pre-implantation TE-like state and then differentiated into the post-implantation cytotrophoblast state? Or, did they differentiate into the cytotrophoblast-like state via amnion-like state?

This is an interesting question that would require further detailed characterisation of cells undergoing transdifferentiation to iTSCs to determine whether primed hESCs are programmed directly to iTSCs or alternatively transit through an amnion-like state or via a reversion to naïve hESCs before committing to a TSC state. We feel that the experiments are beyond the scope of our study, but we have included a discussion of this interest point in the discussion in Lines 545-548.

4. The bright field image of Stable iTSC (Fig 3d) looks very heterogeneous. Are all of the cells shown TSCs? The authors should show flow cytometry results for surface markers reported previously (Okae et al., 2018; Io et al., 2021).

We have reimaged these lines at a later passage and included an updated image representative of the cell line in Fig. 4C.

5. I think the word “reprogramming” is not an appropriate description of the induction of TS cells from ES cells. I suggest “transdifferentiation” or “induction”.

We appreciate the reviewer’s helpful suggestion. This has been corrected throughout the manuscript and we use “transdifferentiation” instead.

6. Fig. 4A shows that TSCs and primary CTBs are segregated by PC1. What are the loading genes of PC1?

We have re-analysed the transcriptional data by including the TE samples. We now see that 3 clusters are formed in the Principal Component Analysis: 1. 5F-iTSCs, primary cytotrophoblast cells, existing hTSCs from previously published datasets, 2. Primary TE and 3. primed hESCs.

Resubmission

MS ID#: DEVELOP/2024/202778

MS TITLE: Transcription factor-based transdifferentiation of human embryonic to trophoblast stem cells

AUTHORS: Paula A Balestrini, Ahmed Abdelbaki, Afshan McCarthy, Liani Devito, Claire E Senner, Alice E Chen, Prabhakaran Munusamy, Paul Blakeley, Kay Elder, Phil Snell, Leila Christie, Paul Serhal, Rabi A Odia, Mahesh Sangrithi, Kathy K Niakan, and Norah ME Fogarty

I have now received all the referees reports on the above manuscript, and have reached a decision. The referees' comments are appended below, or you can access them online: please go to BenchPress and click on the 'Manuscripts with Decisions' queue in the Author Area.

The overall evaluation is positive and we would like to publish a revised manuscript in Development, provided that the referees' comments can be satisfactorily addressed. Please attend to all of the reviewers' comments in your revised manuscript and detail them in your point-by-point

© 2024. Published by The Company of Biologists under the terms of the Creative Commons Attribution License (https://creativecommons.org/licenses/by/4.0/).

response. If you do not agree with any of their criticisms or suggestions explain clearly why this is so. If it would be helpful, you are welcome to contact us to discuss your revision in greater detail. Please send us a point-by-point response indicating your plans for addressing the referees' comments, and we will look over this and provide further guidance.

Reviewer 1

Advance summary and potential significance to field

In the manuscript entitled "Transcription factor-based transdifferentiation of human embryonic to trophoblast stem cells" Balestrini et al. report a transcription factor (TF) -based strategy to convert human ESCs to iTSCs. Based on analysis of TFs expressed in trophectoderm (TE) layer of preimplantation human embryos the authors selected and either inducibly expressed or delivered mRNA of 5 TFs: GATA2, GATA3, KLF5 TFAP2C and MYC into hESCs. In culture conditions supporting hTSCs, after manual selection, they established and expanded self-renewing, bipotent iTSC lines. Their identity was highly similar to the established hTSCs and they showed both ST and EVT differentiation potential. In addition, systematic omission, or combination the 5 TFs revealed critical importance of GATA2 and GATA3 for the ESC to iTSC transdifferentiation process.
While different approaches (both media-based and TF-based) to establish human iTSCs from naÃ¯ve ESCs and MEFs have been reported, this study demonstrates successful TF-based ESCs-to-iTSCs transdifferentiation.
It offers an alternative way to establish patient-derived iTSCs. I believe this manuscript, after revisions, may fulfil the publication criteria of the Development journal.

Comments for the author

Comments:

Line 30: Affiliation no 11 is not linked to a name.

Line 101: 2x cultured Line 112: In addition to Fgf4, mTSCs culture conditions require TGFb/Activin.
Line 115-116: The study by Niwa et al. has indeed demonstrated that overexpression of Cdx2 or (to a much lesser degree) of Eomes in mESCs results in TSC-like cells. Other TFs were tested using similar approach (e.g., Cambuli et al., 2014), however the reprogramming/transdifferentiation was incomplete. For a better overview, it would be useful to mention these studies as well.
Line 124-144: This paragraph is too long and should be substantially shortened.
Line 178-181: Please rephrase; the word "observed" is used three times in three consecutive sentences.
Line 205: Studies by Home et al., 2009 PMID:19700764 and Home et al., 2017 PMID:28232602 should also be referenced here.
Line 433-436: The authors only show DNA methylation of the ELF5 promoter, thus their claim "we demonstrate…that exhibit transcriptional and epigenetic properties consistent with hTSCs" should be toned down.
Figure 5A: What is the % of variance along PC1 and PC2?
Figure 6: The STB differentiation potential of iTSCs should be better characterized. Specifically, the authors are requested (i) to check expression of additional STB markers on the protein level, (ii) to include hTSCs and hTSC-STBs controls in the RTqPCR analysis in panel B, (iii) to calculate the fusion index of iTSCs-STBs and hTSCs-STBs, (iv) to include comparison of iTSC-STBs, hTSC-STBs, and STBs by RNA-seq.
Figure 7: Also, the EVT differentiation potential of iTSCs should be better characterized. The authors are requested (i) to check expression of additional EVT markers on the protein level, (ii) to include hTSCs and hTSC-EVTs controls in the RTqPCR analysis in panel B, (iii) to add HLA-G FACS quantification (iv) to include comparison of iTSC-EVTs, hTSC-EVTs and EVTs by RNA-seq.
Discussion: It would be worth to mention that while reprogramming of MEFs to iTSC using the 4 TFs was successful in the murine system, attempts to reprogram mESCs using the same approach failed (Kubaczka et al., 2015, PMID:26412560).
Discussion: The studies by Liu et al, 2020 and Naama et al., 2023, where the authors used OCT4, KLF4 SOX2, MYC and GATA3, OCT4, KLF4, MYC, respectively, to convert human MEFs into hiTSCs should be discussed in greater detail.

© 2024. Published by The Company of Biologists under the terms of the Creative Commons Attribution License (https://creativecommons.org/licenses/by/4.0/).

Reviewer 2

Advance summary and potential significance to field

This manuscript from Balestrini et al. reports that overexpression of five transcription factors (GATA2, GATA3, TFAP2C, KLF5, and MYC) supports the transdifferentiation of primed human embryonic stem cells (hESCs) into induced trophoblast stem cells (iTSCs). The resulting iTSCs satisfy multiple criteria for bona fide hTSCs. Thus, this work defines a minimal genetic circuitry sufficient for induction of the hTSC state. The authors have responded carefully to questions that came up during the previous round of review. While this work will be of interest to the readers of Development, I have several suggestions for clarification and a few minor experimental suggestions.

Comments for the author

1. The qRT-PCR data in Fig. 3C show strong upregulation of various trophoblast-associated genes in +DOX/-DOX, but the more relevant question is to what extent these genes are induced compared to the starting hESC. Can the authors include this information in the same or a supplemental panel?

2. Syncytiotrophoblasts generated from iTSCs showed detectable hCG expression (Fig. 6C). However, hTSCs sometimes secrete a baseline level of hCG. Did the authors check whether hCG secretion is enhanced during syncytiotrophoblast differentiation? A more quantitative technique, like ELISA, may be needed.

3. In response to Reviewer 2, the authors wrote that they checked expression of the lentiviral transgenes using qPCR primers that distinguish between endogenous and exogenous factors. However, these data are not shown in the manuscript.
Since the 5 factors were expressed from plasmids carrying similar selectable markers, there remains a legitimate question whether all factors are expressed to a similar extent.

4. Line 438-441: “Other groups have reported that resetting hESCs from primed to naïve pluripotency generates a small side-population of hTSCs that can be propagated in culture and recapitulate established hTSCs…” Please revise this statement. As currently written, this statement suggests that hTSCs were generated during the process of primed-to-naïve resetting, but the cited papers reported that fully established naïve hESCs directly and efficiently give rise to hTSCs in Okae media.

5. Line 448-450: “Human naïve ESCs represent an earlier, less-fixed developmental state that reflect gene expression and epigenetic profiles similar to those of the early epiblast or late morula (Gafni et al., 2013; Takashima et al., 2014; Theunissen et al., 2014).” It has been widely reported by multiple independent labs, including in this journal, that Gafni et al. failed to generate naïve ESCs that resemble the early epiblast or late morula. For example, see PMID: 27556940, PMID: 38496581, PMID: 29361568. Therefore, I suggest removing this citation.

First revision

Author response to reviewers' comments

Reviewer 1 Advance Summary and Potential Significance to Field:
In the manuscript entitled “Transcription factor-based transdifferentiation of human embryonic to trophoblast stem cells” Balestrini et al. report a transcription factor (TF) -based strategy to convert human ESCs to iTSCs. Based on analysis of TFs expressed in trophectoderm (TE) layer of preimplantation human embryos, the authors selected and either inducibly expressed or delivered mRNA of 5 TFs: GATA2, GATA3, KLF5, TFAP2C and MYC into hESCs. In culture conditions supporting hTSCs, after manual selection, theyestablished and expanded self- renewing, bipotent iTSC lines. Their identity was highly similar to the established hTSCs and they showed both ST and EVT

© 2024. Published by The Company of Biologists under the terms of the Creative Commons Attribution License (https://creativecommons.org/licenses/by/4.0/).

differentiation potential. In addition, systematic omission, or combination the 5 TFs revealed critical importance of GATA2 and GATA3 for the ESC to iTSC transdifferentiation process. While different approaches (both media-based and TF-based) to establish human iTSCs from naïve ESCs and MEFs have been reported, this study demonstrates successful TF-based ESCs-to-iTSCs transdifferentiation. It offers an alternative way to establish patient-derived iTSCs. I believe this manuscript, after revisions, may fulfil the publication criteria of the Development journal.

Reviewer 1 Comments for the Author:
Comments:

Line 30: Affiliation no 11 is not linked to a name.

Line 101: 2x cultured.
We thank for the reviewer highlighting this error which we have now amended. (Line 94)

Line 112: In addition to Fgf4, mTSCs culture conditions require TGFb/Activin.
Thank you, we have updated the text to include this detail (line 110)

Line 115-116: The study by Niwa et al. has indeed demonstrated that overexpression of Cdx2 or (to a much lesser degree) of Eomes in mESCs results in TSC-like cells. Other TFs were tested using similar approach (e.g., Cambuli et al., 2014), however the reprogramming/transdifferentiation was incomplete. For a better overview, it would be useful to mention these studies as well.

We agree with the reviewer's comment and have expanded on this section and included additional relevant references (lines 114-119).

Line 124-144: This paragraph is too long and should be substantially shortened.

We appreciate the reviewer's comment and we have edited this paragraph as advised. (Lines 126-137)

Line 178-181: Please rephrase; the word "observed" is used three times in three consecutive sentences.

Thank you to the reviewer for noting this. We have edited the text as advised.

Line 205: Studies by Home et al., 2009 PMID:19700764 and Home et al., 2017 PMID:28232602 should also be referenced here. https://pubmed.ncbi.nlm.nih.gov/28232602/
https://pubmed.ncbi.nlm.nih.gov/19700764/

Thank you for the reviewer for bringing the omission of these references to our attention. We have added these as suggested (Line 210).

Line 433-436: The authors only show DNA methylation of the ELF5 promoter, thus their claim "we demonstrate…that exhibit transcriptional and epigenetic properties consistent with hTSCs" should be toned down.

We appreciate this comment and we have rephrased thus: "Significantly here we demonstrate the first successful generation of iTSCs from primed hESCs that exhibit transcriptional properties consistent with hTSCs, and can be maintained long-term in culture and have bipotent differentiation capabilities." (Lines 456-458).

Figure 5A: What is the % of variance along PC1 and PC2?
We have updated the figure 5A to include the % variances of PC1 and PC2. We have added to the text in results "A principal component analysis plotting PC1 against PC2 separated samples into three groups representing TE, hESCs and iTSCs together with all in vitro hTSCs and primary CTBs with PC1 accounting for 49.22% of the variance (Fig. 5A)." (Lines 309-311)

Figure 6: The STB differentiation potential of iTSCs should be better characterized. Specifically, the authors are requested to check expression of additional STB markers on the

© 2024. Published by The Company of Biologists under the terms of the Creative Commons Attribution License (https://creativecommons.org/licenses/by/4.0/).

protein level, to include hTSCs and hTSC-STB controls in the RTqPCR analysis in panel B, to calculate the fusion index of iTSCs-STBs and hTSCs-STBs and to include comparison of iTSC-STBs, hTSC-STBs, and STBs by RNA-seq.

We have performed additional characterisations of STBs generated from 5F-iTSCs and include the following data in Fig. 6:

Fig 6B. We have performed RT-qPCR analysis for STB markers in hTSCs and control STBs. We have normalised expression levels of STBs and 5F-STBs to the starting cell population from which they were derived. Studies of iPSC lines shows that genetic variation imparts donor-specific gene expression, thus normalising to the donor starting cell population removes this effect and allows gene expression in response to differentiation to be quantified (line 331).

Fig 6C. In the interest of space, we have removed 2 of the biological replicates and now show one representative image confirming hCG secretion detected in 5F-STBs.

Fig 6D. We have performed immunofluorescence analysis for the detection of CGB (beta-subunit of hCG) in 5F-STB and undifferentiated 5F-iTSCs. We confirm expression of CGB in 5F-STB and no expression detected in undifferentiated 5F-iTSCs (lines 335- 337).

Fig. 6E. We have performed immunofluorescence analysis for the detection of an additional protein marker of STB, SDC1, and confirm its expression in 56F-STBs (lines 337-338).

Fig. 6F. We have calculated the fusion indices for control STBs and 5F-STBs and confirm that for both, approximately 80% of nuclei are held within syncytia (line 339).

We appreciate the suggestion of additional characterisation of 5F-STBs by RNA-seq and comparative analysis with hTSCs, 5F-iTSCs, hTSC-STBs and primary STBs. However, the focus of this manuscript is the development of a new strategy to transdifferentiate hESC to hTSCs. The generation of STBs from 5F-iTSCs was performed to confirm the differentiation capacity of this new model, hence we did not perform this analysis on these cells. In this regard, to acknowledge that we have not benchmarked on the basis of global transcriptional identity, and to be consistent with the literature, we will refer to the differentiated cell types as "STB-like" and "EVT-like" throughout the manuscript (Dong et al., 2020; Okae et al., 2018).

Figure 7: Also, the EVT differentiation potential of iTSCs should be better characterized. The authors are requested to check expression of additional EVT markers on the protein level to include hTSCs and hTSC-EVTs controls in the RTqPCR analysis in panel B, to add HLA-G FACS quantification and to include comparison of iTSC-EVTs, hTSC-EVTs and EVTs by RNA- seq.

We appreciate the suggestion of additional validation of the EVTs generated from 5F- iTSCs. We have added the following additional data:

Fig 7B. We have performed RT-qPCR analysis for EVT markers in hTSCs and control EVTs. We have normalised expression levels of EVTs and 5F-EVTs to the starting cell population from which they were derived. Studies of iPSC lines shows that genetic variation imparts donor-specific gene expression, thus normalising to the donor starting cell population removes this effect and allows gene expression in response to differentiation to be quantified and remove background genetic effects (line 347).

Fig 7C. We have performed immunofluorescence analysis for the detection of HLA-G expression in 5F-EVTs and confirm specific expression in differentiated EVTs (line 348).

We have not assessed the 5F-EVTs by flow cytometry. We differentiated 5F-iTSCs to EVT-like using the previously published protocol. However, in Okae et al., they passage the EVTs at day 6 and perform flow cytometry. Instead, we fixed the cells at day 6 and performed the molecular analyses on the entire well of resultant cells. It is unclear why cells require passaging at day 6, but it is likely that any cells that have not successfully undergone differentiation to EVT will be lost during the process of passaging. We hypothesis that the percentage efficiencies of EVT differentiation as calculated by flow cytometry are likely

© 2024. Published by The Company of Biologists under the terms of the Creative Commons Attribution License (https://creativecommons.org/licenses/by/4.0/).

overestimates.

Following the same reasoning as for the STBs mentioned above, we have not performed global transcriptional analysis of these cells. We have performed qRT-PCR analysis and validation of protein expression of HLAG. We did not perform flow cytometry analysis to quantify the efficiency of EVT differentiation as the objective of the experiment was to assess their ability to generate EVT-like cells. Okae and collaborators acknowledge that there is line-to-line variability in the ability to generate EVT. We have updated the nomenclature in the manuscript to refer to these as EVT-like cells, to be consistent with literature infield and to acknowledge that they have not been benchmarked against the primary cell type.

Discussion: It would be worth to mention that while reprogramming of MEFs to iTSC using the 4 TFs was successful in the murine system, attempts to reprogram mESCs using the same approach failed (Kubaczka et al., 2015, PMID:26412560).

We thank the reviewer for the suggestion of adding this detail, which we have (Lines 413-415).

Discussion: The studies by Liu et al, 2020 and Naama et al., 2023, where the authors used OCT4, KLF4,SOX2, MYC and GATA3, OCT4, KLF4, MYC, respectively, to convert human MEFs into hiTSCs should be discussed in greater detail.

We thank the reviewer for this comment, and we have expanded on these important studies in the discussion. (Lines 494-507 and 550-557)

***** Reviewer 2 Advance Summary and Potential Significance to Field:
This manuscript from Balestrini et al. reports that overexpression of five transcription factors (GATA2, GATA3, TFAP2C, KLF5, and MYC) supports the transdifferentiation of primed human embryonic stem cells (hESCs) into induced trophoblast stem cells (iTSCs). The resulting iTSCs satisfy multiple criteria for bona fide hTSCs. Thus, this work defines a minimal genetic circuitry sufficient for induction of the hTSC state. The authors have responded carefully to questions that came up during the previous round of review. While this work will be of interest to the readers of Development, I have several suggestions for clarification and a few minor experimental suggestions.

Reviewer 2 Comments for the Author:

1. The qRT-PCR data in **Fig. 3C** show strong upregulation of various trophoblast- associated genes in +DOX/-DOX, but the more relevant question is to what extent these genes are induced compared to the starting hESC. Can the authors include this information in the same or a supplemental panel?

We thank the reviewers for this comment. We have re-analysed the time-course qRT- PCR data normalising the gene expression to 5F-hESCs in pluripotency media (day 0). Here we observe upregulation of endogenous GATA2 and GATA3 from day 5. However, we observed by brightfield microscopy widespread differentiation when hESCs are transferred into hTSC media after 48 hr. Differentiation continues, plates get very overgrown, cell death and then they cannot be passaged. If the reviewer feels strongly, we can put this into the supplementary figure.

© 2024. Published by The Company of Biologists under the terms of the Creative Commons Attribution License (https://creativecommons.org/licenses/by/4.0/).

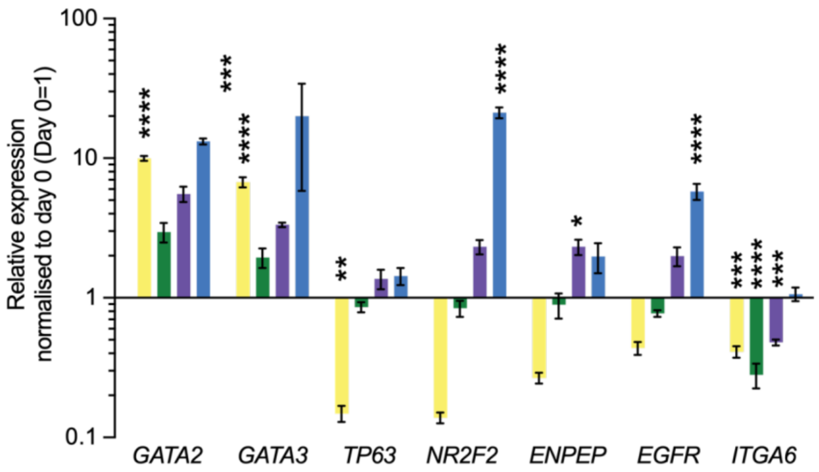

Fig: Time-course qRT-PCR analysis for the detection of endogenous GATA2, GATA3, TP63, NR2F2, ENPEP, EGFR and ITGA6 in 5F-hESCs across 20 days of dox-induction in hTSC media. Relative expression is reflected as fold change over 5F-hESCs cultured in mTSeR pluripotency media normalized to GAPDH.

2. Syncytiotrophoblasts generated from iTSCs showed detectable hCG expression (**Fig. 6C**). However, hTSCs sometimes secrete a baseline level of hCG. Did the authors check whether hCG secretion is enhanced during syncytiotrophoblast differentiation? A more quantitative technique, like ELISA, may be needed.

The reviewer raises an important point. Dogma had held that hCG was produced by the syncytiotrophoblast, and hCG production was a defining characteristic of syncytilisation (Hoshina et al., 1985). However, it has been since that hCG is secreted by both cytotrophoblast cells and syncytiotrophoblast (Snegovskikh et al., 2997) as well as some tumours (Sisinni and Landriscina, 2015). Here, we have performed immunofluorescence analysis for the detection of CGB, which is the protein encoding the beta-subunit of secreted hCG. We confirmed that differentiated STB from 5F-iTSCs robustly express this protein, and we do not detect its expression in undifferentiated 5F-iTSCs (Fig. 6D). (Lines 335-337)

3. In response to Reviewer 2, the authors wrote that they checked **expression** of the **lentiviral transgenes** using qPCR primers that distinguish between endogenous and exogenous factors. However, these data are not shown in the manuscript. Since the 5 factors were expressed from plasmids carrying similar selectable markers, there remains a legitimate question whether all factors are expressed to a similar extent.

We thank the reviewers for this question. Indeed, it was imperative to confirm that all 5 transgenes were integrated and expressed. As we described in the manuscript, and according to the manufacturer's protocol, we generate clonal 5F-hESCs and cultured them in the presence of doxycycline for 48 hrs. Transgene expression was quantified using qRT-PCR distinguishing exogenous expression for KLF5, GATA2, GATA3 and MYC. It was difficult to design primers that could specifically distinguish exogenous expression from the endogenous. Instead for TFAP2C we performed immunofluorescence analysis for the detection of TFAP2C expression in hESCs. We present below the qRT-PCR and IF analysis of the clone that we used for generating 5F- iTSCs. While we had different levels of transgene expression, as it is impossible to control the amount of lentivirus integration, we had 400 - 20,000 fold increase expression when compared with uninduced 5F-hESCs, confirming no "leaky" transgene expression. We have added this figure to supplementary figure 1B and C. (Line 236-237).

© 2024. Published by The Company of Biologists under the terms of the Creative Commons Attribution License (https://creativecommons.org/licenses/by/4.0/).

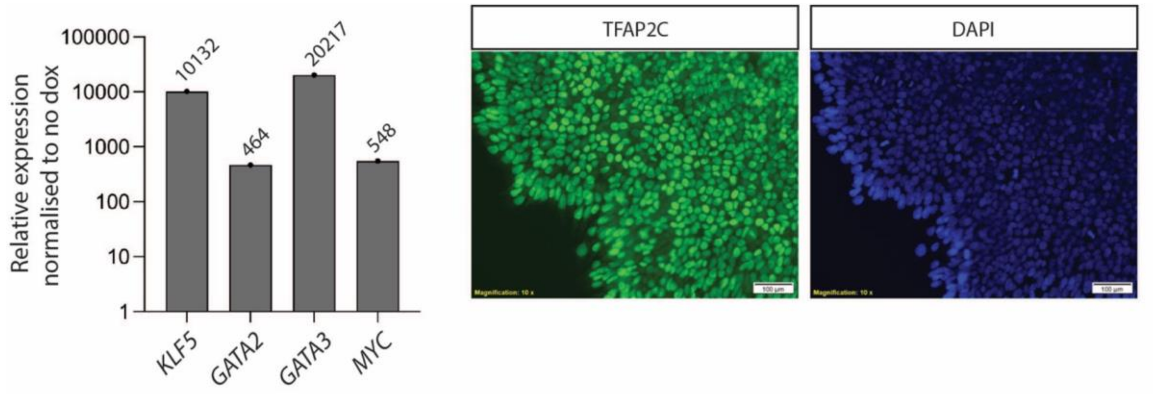

Figure: hESCs were first transduced with only the LVX-Tet3G lentivirus, followed by selection with G418. Resistant cells were then transduced with a pool of LVX-TRE3G- GOI encoding GATA2, GATA3, TFAP2C, MYC and KLF5. Doubly transduced cells were selected using puromycin and the resulting puromycin-resistant clones were screened for GOI inducibility. Clones were cultured in the presence of dox for 48 hr before analysis by either qRT-PCR or immunofluorescence for the detection of endogenous transcription factor expression. qRT-PCR data was analysed thus, relative expression is reflected as fold change over the uninduced clone cultured in hESCs media normalized to *GAPDH*. *TFAP2C* expression was detected by immunofluorescence due to difficulties in designing primers that can distinguish between endogenous and exogenous TFAP2C.

4. **Line 438-441**: “Other groups have reported that resetting hESCs from primed to naïve pluripotency generates a small side-population of hTSCs that can be propagated in culture and recapitulate established hTSCs…” Please revise this statement. As currently written, this statement suggests that hTSCs were generated during the process of primed-to-naïve resetting, but the cited papers reported that fully established naïve hESCs directly and efficiently give rise to hTSCs in Okae media.

We thank the reviewer for this comment and have edited the wording as follows: “Previously studies have shown that while naïve hESCs cultured in hTSC media could be converted into hTSCs, primed hESCs had elevated cell death and lost proliferative capacity similar to what we observed in this study (Castel et al., 2020; Cinkornpumin et al., 2020; Dong et al., 2020; Liu et al., 2020).” Lines (454-456)

5. **Line 448-450**: “Human naïve ESCs represent an earlier, less-fixed developmental state that reflect gene expression and epigenetic profiles similar to those of the early epiblast or late morula (Gafni et al., 2013; Takashima et al., 2014; Theunissen et al., 2014).” It has been widely reported by multiple independent labs, including in this journal, that Gafni et al. failed to generate naïve ESCs that resemble the early epiblast or late morula. For example, see PMID: 27556940, PMID: 38496581, PMID: 29361568. Therefore, I suggest removing this citation.

We thank the reviewer for this comment and have removed this reference, as suggested.

References:

Castel, G., Meistermann, D., Bre2n, B., Firmin, J., Blin, J., Loubersac, S., Bruneau, A., Chevolleau, S., Kilens, S., Chariau, C., Gaignerie, A., Francheteau, Q., Kagawa, H., Charpen2er, E., Flippe, L., Francois-Campion, V., Haider, S., Dietrich, B., Knofler, M., Arima, T., Bourdon, J., Rivron, N., Masson, D., Fournier, T., Okae, H., Freour, T., David, L., 2020. Induc2on of Human Trophoblast Stem Cells from Soma2c Cells and Pluripotent Stem Cells. Cell Rep 33, 108419.

Cinkornpumin, J.K., Kwon, S.Y., Guo, Y., Hossain, I., Sirois, J., RusseY, C.S., Tseng, H.W., Okae, H., Arima, T., Duchaine, T.F., Liu, W., Pastor, W.A., 2020. Naive Human Embryonic Stem Cells Can Give Rise to Cells with a Trophoblast-like Transcriptome and Methylome. Stem Cell Reports 15, 198-213.

© 2024. Published by The Company of Biologists under the terms of the Creative Commons Attribution License (https://creativecommons.org/licenses/by/4.0/).

Dong, C., Beltcheva, M., Gontarz, P., Zhang, B., Popli, P., Fischer, L.A., Khan, S.A., Park, K.M., Yoon, E.J., Xing, X., Kommagani, R., Wang, T., Solnica-Krezel, L., Theunissen, T.W., 2020. Deriva2on of trophoblast stem cells from naive human pluripotent stem cells. Elife 9.

Hoshina, M., Boothby, M., Hussa, R., Pa`llo, R., Camel, H.M., Boime, I., 1985. Linkage of human chorionic gonadotrophin and placental lactogen biosynthesis to trophoblast differen2a2on and tumorigenesis. Placenta 6, 163-172.

Liu, X., Ouyang, J.F., Rossello, F.J., Tan, J.P., Davidson, K.C., Valdes, D.S., Schroder, J., Sun, Y.B.Y., Chen, J., Knaupp, A.S., Sun, G., Chy, H.S., Huang, Z., Pflueger, J., Firas, J., Tano, V., Buckberry, S., Paynter, J.M., Larcombe, M.R., Poppe, D., Choo, X.Y., O'Brien, C.M., Pastor, W.A., Chen, D., Leichter, A.L., Naeem, H., Tripathi, P., Das, P.P., Grubman, A., Powell, D.R., LasleY, A.L., David, L., Nilsson, S.K., Clark, A.T., Lister, R., Nefzger, C.M., MarteloYo, L.G., Rackham, O.J.L., Polo, J.M., 2020. Reprogramming roadmap reveals route to human induced trophoblast stem cells. Nature 586, 101-107.

Okae, H., Toh, H., Sato, T., Hiura, H., Takahashi, S., Shirane, K., Kabayama, Y., Suyama, M., Sasaki, H., Arima, T., 2018. Deriva2on of Human Trophoblast Stem Cells. Cell Stem Cell 22, 50- 63 e56.

Sisinni, L., Landriscina, M., 2015. The Role of Human Chorionic Gonadotropin as Tumor Marker: Biochemical and Clinical Aspects. Adv Exp Med Biol 867, 159-176.

Snegovskikh, V., Hodgson, E., Sfakianaki, A., Funai, E., Ma, Y., Guller, S., Rahman, M., Buhimschi, C., Buhimschi, I., Norwitz, E., 2997. Thrombin suppresses hCG produc2on by human syncy2otrophoblast: A mechanism by which first trimester bleeding causes abor2on. American Journal of Obstetrics and Gynaecology 197.

Second decision letter

MS ID#: DEVELOP/2024/202778

MS TITLE: Transcription factor-based transdifferentiation of human embryonic to trophoblast stem cells

AUTHORS: Paula A Balestrini, Ahmed Abdelbaki, Afshan McCarthy, Liani Devito, Claire E Senner, Alice E Chen, Prabhakaran Munusamy, Paul Blakeley, Kay Elder, Phil Snell, Leila Christie, Paul Serhal, Rabi A Odia, Mahesh Sangrithi, Kathy K Niakan, and Norah ME Fogarty

I have now received all the referees reports on the above manuscript, and have reached a decision. The referees' comments are appended below, or you can access them online: please go to BenchPress and click on the 'Manuscripts with Decisions' queue in the Author Area.

The overall evaluation is positive and we would like to publish a revised manuscript in Development. As you will see the Reviewer has identified some very minor points that I hope you can address before uploading the final version of your manuscript. The manscript will not require re-review, rather I will look it over myself prior to acceptance.

Reviewer 2

Advance summary and potential significance to field

See my prior review

Comments for the author

The authors have carefully revised their manuscript in response to the reviewers’ comments and the paper is now ready for publication in Development. I only have a few minor suggestions remaining:

© 2024. Published by The Company of Biologists under the terms of the Creative Commons Attribution License (https://creativecommons.org/licenses/by/4.0/).

1. In response to my question #1, the authors offer to include normalization of their time course qRT-PCR data relative to the d0 time point. I agree these data would be helpful to include in the Supplement. Given the confusion in the field, it would also be useful to include brightfield images demonstrating the fate of uninduced primed hESCs in hTSC media (e.g. differentiation, overgrowth, and cell death).

2. Line 95-99: “induced trophoblast stem cells (iTSCs) which resemble primary tissue-derived hTSCs have been captured in the process of reprogramming fibroblasts to human embryonic stem cells (hESCs) in naïve cell culture conditions (Castel et al., 2020; Cinkornpumin et al., 2020; Dong et al., 2020; Liu et al., 2020; Naama et al., 2023). In addition, established naïve ES cells cultured in conditions which inhibit ERK and Nodal signaling also yield iTSCs (Guo et al., 2021).”

This paragraph should be corrected. Cinkornpumin et al. and Dong et al. did not generate iTSCs in the process of reprogramming, but directly derived hTSCs from naïve hESCs. The authors described this correctly in the Discussion on line 458-460.

3. Please check that all cited papers are included in the list of references. For example, Takashima et al., 2014 and Theunissen et al., 2014 are cited on line 442, but not included among the references.

Second revision

<u>Author response to reviewers' comments</u>

***** Reviewer 2 Advance Summary and Potential Significance to Field: See my prior review

Reviewer 2 Comments for the Author:

The authors have carefully revised their manuscript in response to the reviewers’ comments and the paper is now ready for publication in Development. I only have a few minor suggestions remaining:

1. In response to my question #1, the authors offer to include normalization of their time course qRT-PCR data relative to the d0 time point. I agree these data would be helpful to include in the Supplement.

We have included this data in Fig S1D. The final figure is as presented here:

© 2024. Published by The Company of Biologists under the terms of the Creative Commons Attribution License (https://creativecommons.org/licenses/by/4.0/).

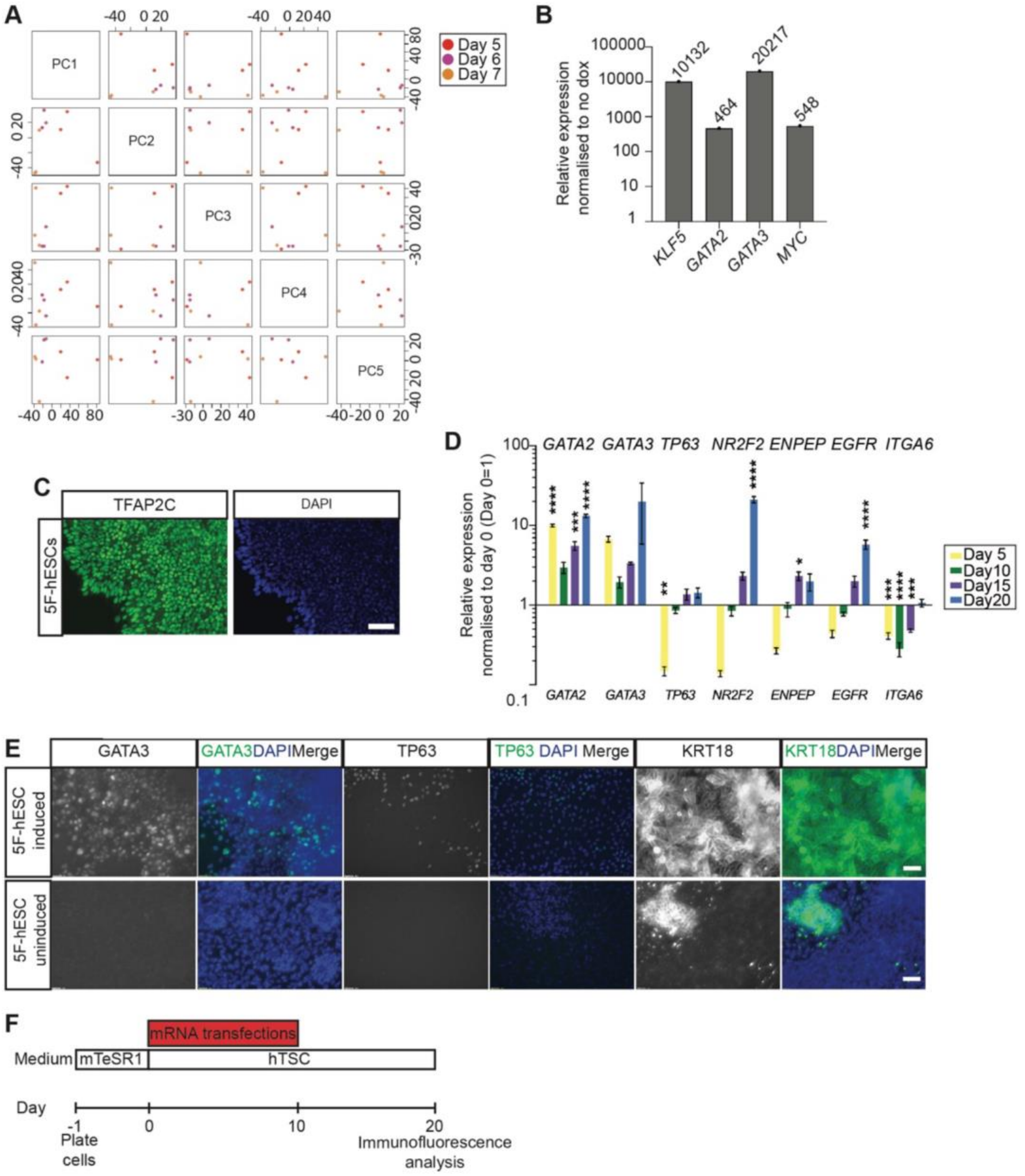

Figure S1
(A) Principal component analysis matrix using the first five principal components for the top 12,000 most variable expressed genes in day 5, 6 and 7 TE samples. (B) RT-qPCR analysis for the detection of exogenous *KLF5*, *GATA2*, *GATA3* and *MYC* in 5F-hESCs after 48 h of doxycycline treatment. Relative expression is reflected as fold change over uninduced 5F-hESCs cultured normalized to *GAPDH*. (C) Immunofluorescence analysis for the detection of TFAP2C in 5F-hESCs after 48 h of doxycycline. (D) Time-course qRT-PCR analysis for the detection of endogenous *GATA2*, *GATA3*, *TP63*, *NR2F2*, *ENPEP*, *EGFR* and *ITGA6* in 5F-hESCs across 20 days of dox-induction in hTSC media. Relative expression is reflected as fold change over 5F-hESCs at day 0 plated in mTSeR pluripotency media, normalized to *GAPDH*. (E) Immunofluorescence analysis for the detection of GATA3, TP63 and KRT18 (green) and DAPI nuclear expression (blue) in doxycycline-induced and uninduced 5F-hESCs on day 20. Scale bars: 50 µm. (F) Schematic representation of the strategy for combinatorial transdifferentiation experiments using modified mRNAs.

Given the confusion in the field, it would also be useful to include brightfield images demonstrating the fate of uninduced primed hESCs in hTSC media (e.g. differentiation, overgrowth, and cell death).
Brightfield imaging of uninduced hESCs in hTSC media has already been included in the main figures in Fig 3D "Day 20 - dox." We have updated the labelling of the panelling in this figure to

© 2024. Published by The Company of Biologists under the terms of the Creative Commons Attribution License (https://creativecommons.org/licenses/by/4.0/).

make it clear that these panels are independent experiments. The final figure is as presented here:

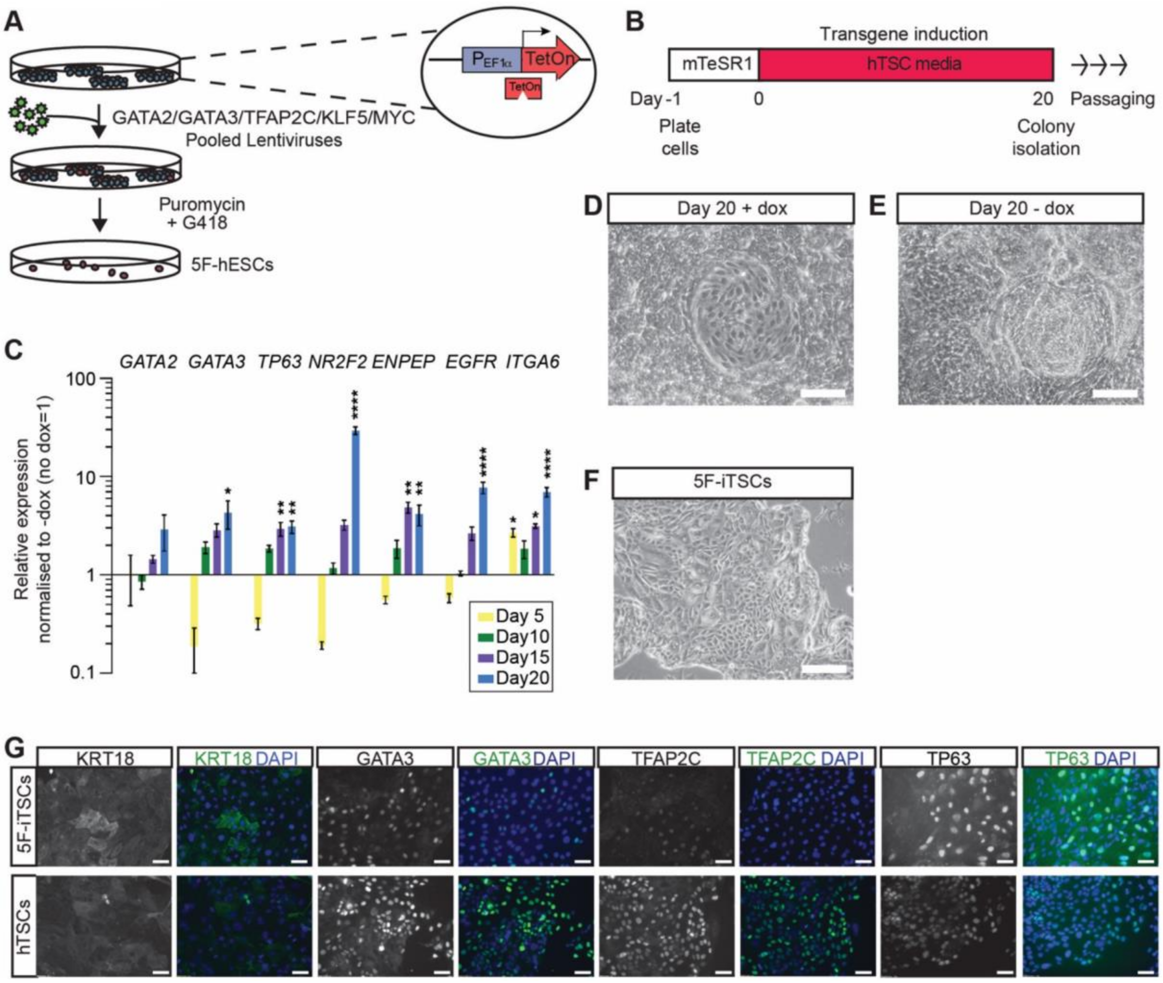

Figure 3 - Induced of expression of GATA2, GATA3, TFAP2C and KLF5 for 20 days programs hESCs to hTSC-like cells
(A) Schematic diagram of the generation of GATA2, GATA3, TFAP2C, KLF5 and MYC inducible hESCs by lentiviral transduction. (B) Schematic representation of the strategy for transdifferentiation of 5F-hESCs to hTSCs. 5F-hESCs were plated in mTeSR1 media for 24 h after which media was replaced with hTSC media. Doxycycline was administered daily for 20 days in hTSC media for transgene induction. (C) Time-course RT-qPCR analysis for the detection of endogenous *GATA2*, *GATA3*, *TP63*, *NR2F2*, *ENPEP*, *EGFR* and *ITGA6* in 5F-hESCs across 20 days of dox-induction in hTSC media. Relative expression is reflected as fold change over uninduced 5F-hESCs cultured in hTSC media normalized to *GAPDH*. Data are mean ± SEM of n=3 biological replicates analysed with a one-way ANOVA with Dunnett's post hoc test (*p<0.05, **p<0.01, ****p<0.001). (D) Brightfield images of 5F-hESCs cultured in hESCs cultured in hTSC media in the presence of doxycycline for 20 days (+dox) (E) Brightfield images of 5F-hESCs cultured in hTSC media in the absence of dox for 20 days (- dox). (F) Brightfield images of stable 5F-iTSC lines derived from transgene overexpression grown for 15 passages. Scale bar: 200 µm. (G) Immunofluorescence analysis for the detection of KRT18, GATA3, TFAP2C and TP63 (green) and DAPI nuclear staining (blue) in stable 5F-iTSCs and previously established control hTSCs. Scale bars: 50 µm.

2. Line 95-99: "induced trophoblast stem cells (iTSCs) which resemble primary tissue-derived hTSCs have been captured in the process of reprogramming fibroblasts to human embryonic stem cells (hESCs) in naïve cell culture conditions (Castel et al., 2020; Cinkornpumin et al., 2020; Dong et al., 2020; Liu et al., 2020; Naama et al., 2023). In addition, established naïve ES cells cultured

© 2024. Published by The Company of Biologists under the terms of the Creative Commons Attribution License (https://creativecommons.org/licenses/by/4.0/).

in conditions which inhibit ERK and Nodal signaling also yield iTSCs (Guo et al., 2021)."

This paragraph should be corrected. Cinkornpumin et al. and Dong et al. did not generate iTSCs in the process of reprogramming, but directly derived hTSCs from naïve hESCs. The authors described this correctly in the Discussion on line 458-460.
We have corrected this paragraph. Lines 98-99 is highlighted to show these edits.

3. Please check that all cited papers are included in the list of references. For example, Takashima et al., 2014 and Theunissen et al., 2014 are cited on line 442, but not included among the references.
We thank the reviewer for picking up on this. We have checked all citations are present and correct in the bibliography.

Third decision letter

MS ID#: DEVELOP/2024/202778

MS TITLE: Transcription factor-based transdifferentiation of human embryonic to trophoblast stem cells

AUTHORS: Paula A Balestrini, Ahmed Abdelbaki, Afshan McCarthy, Liani Devito, Claire E Senner, Alice E Chen, Prabhakaran Munusamy, Paul Blakeley, Kay Elder, Phil Snell, Leila Christie, Paul Serhal, Rabi A Odia, Mahesh Sangrithi, Kathy K Niakan, and Norah ME Fogarty

I am happy to tell you that your manuscript has been accepted for publication in Development, pending our standard publication integrity checks.

© 2024. Published by The Company of Biologists under the terms of the Creative Commons Attribution License (https://creativecommons.org/licenses/by/4.0/).
